# Dissecting the loci underlying maturation timing in Atlantic salmon using haplotype and multi-SNP based association methods

**DOI:** 10.1038/s41437-022-00570-w

**Published:** 2022-11-10

**Authors:** Marion Sinclair-Waters, Torfinn Nome, Jing Wang, Sigbjørn Lien, Matthew P. Kent, Harald Sægrov, Bjørn Florø-Larsen, Geir H. Bolstad, Craig R. Primmer, Nicola J. Barson

**Affiliations:** 1 Organismal and Evolutionary Biology Research Programme, Faculty of Biological and Environmental Sciences University of Helsinki, Helsinki, Finland; 2 Institute of Biotechnology, Helsinki Institute of Life Science (HiLIFE), University of Helsinki, Helsinki, Finland; 3 Centre for Integrative Genetics, Department of Animal and Aquacultural Sciences, Faculty of Biosciences, Norwegian University of Life Sciences, Ås, Norway; 4 Key laboratory for Bio-Resources and Eco-Environment, College of Life Science, Sichuan University, Chengdu, China; 5 Rådgivende Biologer, Bergen, Norway; 6 Norwegian Veterinary Institute, Trondheim, Norway; 7 Norwegian Institute for Nature Research (NINA), Trondheim, Norway

**Keywords:** Evolutionary genetics, Genetic association study

## Abstract

Characterizing the role of different mutational effect sizes in the evolution of fitness-related traits has been a major goal in evolutionary biology for a century. Such characterization in a diversity of systems, both model and non-model, will help to understand the genetic processes underlying fitness variation. However, well-characterized genetic architectures of such traits in wild populations remain uncommon. In this study, we used haplotype-based and multi-SNP Bayesian association methods with sequencing data for 313 individuals from wild populations to test the mutational composition of known candidate regions for sea age at maturation in Atlantic salmon (*Salmo salar*). We detected an association at five loci out of 116 candidates previously identified in an aquaculture strain with maturation timing in wild Atlantic salmon. We found that at four of these five loci, variation explained by the locus was predominantly driven by a single SNP suggesting the genetic architecture of this trait includes multiple loci with simple, non-clustered alleles and a locus with potentially more complex alleles. This highlights the diversity of genetic architectures that can exist for fitness-related traits. Furthermore, this study provides a useful multi-SNP framework for future work using sequencing data to characterize genetic variation underlying phenotypes in wild populations.

## Introduction

The debate over the role of different mutational effect sizes in the evolution of fitness has been ongoing for a century (Orr 1998; Orr 1999; Fisher 1930; Rockman 2012; Remington 2015; Kimura 1983). Our ability to now sequence large numbers of individuals has greatly informed this debate. Additionally, an explosion in the number of association studies has revealed the architecture of important traits. These have revealed examples of large effect loci (Hoekstra 2006; Linnen et al. 2013; Tishkoff et al. 2007; Daborn et al. 2002), but also many cases of polygenicity (Purcell et al. 2009; Loh et al. 2015; Pritchard and Di Rienzo 2010). However, even where large effect alleles are known, these can have been generated over time via sequential mutations of smaller effect that allow a slow walk to the optimum as predicted by Orr (1998), or a single mutation of large effect. The difference between these scenarios is important for our understanding of the process of adaptation, the adaptability of organisms and their resilience to rapid environmental change (Oomen et al. 2020; Kardos and Luikart 2021; Yeaman et al. 2018).

Among genome-wide association studies published to date, many complex traits appear to be polygenic (Visscher et al. 2017; Fisher 1918; Pritchard and Di Rienzo 2010) or omnigenic (i.e. affected by a large proportion of genes through a network of core genes and many peripheral genes that modify the effects of core genes, whereby a large proportion of all genes expressed in trait-related tissues has some effect on the trait) (Boyle et al. 2017; Liu et al. 2019). Although polygenicity is widespread, an increasing number of examples of major effect loci exist, whereby one locus explains a large proportion of the phenotypic variation (Barson et al. 2015; Linnen et al. 2013). In some cases, major effect loci can contain multiple tightly linked genes, coined “supergenes”, where localized reduction in recombination is often caused by larger chromosomal rearrangements. For example, this phenomenon is known to underlie phenotypic variation observed among ruff (*Philomachus pugnax*) mating morphs (Lamichhaney et al. 2015; Küpper et al. 2015), Atlantic cod (*Gadus morhua*) (Kirubakaran et al. 2016; Sinclair-Waters et al. 2018) and rainbow trout (*Oncorhynchus mykiss*) migratory ecotypes (Pearse et al. 2018), and *Heliconius* butterfly wing-pattern morphs (Joron et al. 2011). More recent work has found that major effect loci can exist alongside a polygenic background where loci with a variety of effect sizes underlie trait variation (Sinnott-Armstrong et al. 2020; Sinclair-Waters et al. 2020). Such mixed genetic architectures may be pervasive, but currently remain undetected due to the large sample sizes required for detecting loci with smaller effects (Sinclair-Waters et al. 2020) and it is possible that additional examples are to be found with future higher-powered studies. Although studies aimed at resolving genotype-phenotype links are mounting, well-characterized genetic architectures of fitness-related traits, particularly in natural populations, are still uncommon.

While some trait-associated loci have been identified, such findings lead to other crucial questions: How have trait-locus associations arisen? Has the locus arisen through a single or multiple new mutation(s)? Or alternatively, did the locus emerge via recombination that gave rise to new combinations of existing variants? Numerous studies from the past decade have shown that major effect loci involve the cumulative effects of multiple mutations, rather than a single mutation, thus highlighting the relevance of considering the latter scenarios. For example, Bickel et al. (2011) found that ~60% of variation in female abdominal pigmentation in *Drosophila melanogaster* can be explained by sequence variation at the *bab* locus, but a GWAS (genome-wide association study) analyzing the same trait did not identify a single SNP in *bab* that passed the genome-wide significance threshold. Alleles consisting of multiple SNPs were associated with high proportions of the variation, whereas single SNPs had only small effects and were therefore missed in the single-SNP GWAS. Additionally, Linnen at al. (2013) and Kerdaffrec et al. (2016) also identify multiple mutations within a confined region that have cumulative effects on colour traits in deer mice and seed dormancy in *Arabidopsis thaliana*, respectively. In natural populations with gene flow such as in Linnen et al. (2013) and Kerdaffrec et al. (2016), this is perhaps not unexpected as theory predicts that clustered and major effect loci will evolve under such scenarios (Yeaman and Whitlock 2011; Yeaman 2013). Given these findings, examining extended sequence haplotypes containing multiple SNPs, rather than each SNP independently, is important (Remington 2015). This can be achieved by using alternative strategies that look at combined effects of variants, rather than single-SNP methods typically used in GWAS.

Here we investigate the genetic basis of Atlantic salmon (*Salmo salar*) sea age at maturity—the number of years spent in the marine environment before reaching maturity and returning to the natal river (freshwater) to reproduce. Atlantic salmon individuals can spend anywhere from one to five years in the marine environment before maturation occurs. Prior to this marine phase, individuals spend one to seven years in their natal river before migrating to the sea. Moreover, some individuals will reach maturity in freshwater without ever having migrated to the sea, known as mature parr (Fleming 1996; Mobley et al. 2021). This variation in maturation timing contributes substantially to the diversity of life history strategies among Atlantic salmon (Erkinaro et al. 2019). Age at maturity varies both within and among Atlantic salmon populations, with multiple maturation age classes commonly occurring within single populations (Barson et al. 2015; Jonsson et al. 1991; Hutchings and Jones 1998). Furthermore, age at maturity is an important life history trait affecting fitness traits such as survival, size at maturity and reproductive success (Stearns 2000; Mobley et al. 2021). Substantial variation in Atlantic salmon sea age at maturity is maintained due to a trade-off between mating success at spawning grounds and survival, whereby individuals that mature later are larger and have higher reproductive success on the spawning grounds, but have a lower chance of surviving until reproductive age due to a high mortality in the marine environment (Chaput 2012). In contrast individuals that mature early are smaller and have lower reproductive success, but higher survival and thus higher chance of reaching reproductive age (Fleming and Einum 2011; Mobley et al. 2020).

Variation in maturation timing in Atlantic salmon is highly heritable (Gjerde 1984; Sinclair-Waters et al. 2020; Reed et al. 2018) and consequently there is substantial interest in understanding the underlying genetic architecture. A large-effect locus on chromosome 25 explaining up to 39% of the variation in sea age at maturity was found in wild European populations (Barson et al. 2015) and domesticated salmon (Ayllon et al. 2015). The primary candidate gene underlying the association of this locus is *vgll3* due to its close proximity to the associated SNP variation (Sinclair-Waters et al. 2022; Ayllon et al. 2015; Barson et al. 2015) and its known function in other species. The *vgll3* gene encodes a transcription cofactor that, amongst other things, regulates adipogenesis (Halperin et al. 2013) and is associated with variation in puberty timing in humans (Day et al. 2017; Perry et al. 2014). In addition to *vgll3*, Sinclair-Waters et al. (2020) identified 119 other candidate genes for male maturation in a GWAS using SNP-array data and including >11,000 males from an Atlantic salmon aquaculture strain originating since the 1970s and derived of founder individuals from 41 wild Norwegian rivers (Gjedrem et al. 1991). Two particularly strong associations between maturation timing were found on chromosome 9 in close proximity to *six6* and chromosome 25, *vgll3*. The association of *six6* was also found by Barson et al. (2015) in wild Atlantic salmon, but the signal disappeared after correction for the confounding effects of population structure that can increase the false positive rate of association tests (Price et al. 2010). Barson et al. (2015), however, focused solely on single-SNP associations via GWAS without considering the possible influence of combined variant effects. Interestingly, the *six6* gene is also associated with age at maturity in two Pacific salmon species (Waters et al. 2021; Willis et al. 2020), humans (Perry et al. 2014) and cattle (Cánovas et al. 2014).

Characterization of genetic architecture for fitness-related traits in a number of organisms, including both model and non-model systems, and for a variety of traits, will help gain a clearer understanding of the processes underlying fitness variation (Stinchcombe and Hoekstra 2008). However, studies using sequencing data to examine variation associated with important fitness-related traits in wild populations are limited. Fortunately, due to developments in sequencing technologies and bioinformatics, studies using this approach are likely to rise in number. We therefore aim to provide a useful and timely framework for characterizing genetic variation underlying phenotypes in wild populations in the future. Here, we focus on characterizing the mutational composition of known candidate regions for sea age at maturity in wild Atlantic salmon found in the previous large sample GWAS, Sinclair-Waters et al. (2020), by testing for combined effects of variants to determine number and location of associated variants. We integrate re-sequencing data and phenotype information for 313 individuals from 53 wild population of Atlantic salmon with alternative GWAS strategies that consider the combined effects of variants, rather than single-SNP effects. This approach can provide better resolution of the variants underlying fitness-related traits when combined effects are involved, while also effective for detecting single SNP effects.

## Materials and methods

### Study material

Whole genome sequencing data were obtained for 313 wild individuals collected from 53 Norwegian and Finnish populations spanning the Norwegian coast and to the Barents sea in the north (59°N–71°N) (Supplementary Table S1, S2, Fig. S1) previously reported in Bertolotti et al. (2020). The 313-individual dataset includes individuals for which the sea age at maturity phenotype has been recorded, and spans populations belonging to both the Atlantic and Barents/White sea phylogeographic groups. Additionally, sampling locations were chosen to represent populations exhibiting both within and among population variation in sea-age at maturity (Barson et al. 2015). These geographic regions were studied in Barson et al. (2015) using a single SNP approach based on SNP-array data. Whole genome sequencing data allows variants not present on the SNP-array and combined SNP effects to be tested. Scales were collected from individuals and sea age was determined by examining scale growth rings as described in Mobley et al. (2021) and ICES (2011). Individuals were categorized into three maturation categories based on the number of years spent at sea prior to their first return migration to rivers for spawning: 1 (one year spent at sea), 2 (two years spent at sea), or 3 (three or more years spent at sea). Only five individuals had spent four years and were therefore combined with three-year fish for all analyses.

### SNP calling and filtering

Variant calling and the first round of filtering was done in a larger set of individuals described in Bertolotti et al. (2020). Raw Illumina reads were mapped to the Atlantic salmon genome (ICSASG_v2) (Lien et al. 2016) using *bcbio-nextgen v.1.1* (Chapman et al. 2020) with the *bwa-mem aligner v.0.7.17* (Heng Li 2013). Genomic variation was identified using the Genome Analysis Toolkit (*GATK*) *v4.0.3.0*., following *GATK*’s best practice recommendations. *Picard v2.18.7* (Picard Toolkit 2019) was used to mark duplicates and *GATK* was used for joint calling (Depristo et al. 2011). Variants were annotated using *SNPeff v. 4.3* (Cingolani et al. 2012). Variant call were further filtered with GATK’s variant filtration according to the following –*filterExpression*: “MQRankSum < −12.5 || ReadPosRankSum < −8.0 || QD < 2.0 || FS > 60.0 || (QD < 10.0 && AD[0:1] / (AD[0:1] + AD[0:0]) < 0.25 && ReadPosRankSum < 0.0) || MQ < 30.0”. SNPs were then filtered using *SNPable* procedure (Li 2009), where 100 bp kmers are mapped to reference genome (ICSASG_v2) using Burrows-Wheeler Aligner (*bwa aln*) (Li and Durbin 2009), and only SNPs within regions with reads that uniquely map are retained. We then removed additional SNPs with *vcftools* using the following criteria: –*min-alleles 2*, –*max-alleles 2*, –*maf 0.0000000001*, –*max-missing 0.7*, –*remove-indels*, –*minGQ 10*, and –*minDP 4*. A subset 313 individuals from wild populations was then extracted from this larger dataset using *vcftools* (Danecek et al. 2011). This reduced dataset was used for all subsequent analyses.

### Analysis of principal components used for population structure correction and genetic differentiation

We produced a reduced SNP dataset by pruning one SNP from each SNP pair with a correlation coefficient (*r*^*2*^) greater than 0.2 within a 50 kb block using the –*indep-pairwise 50 10 0.2* function implemented in *PLINK v1.9* (Purcell et al. 2007). This yielded 403,540 SNPs to examine population structure using a principal component analysis, *smartpca*, implemented in the EIGENSOFT *v5* software (Patterson et al. 2006). Principal components were then used to correct for the confounding effects of population structure during association testing (see below). Additionally, this reduced dataset was used to estimate *F*_ST_ (following Weir and Cockerham (1984)) between the Atlantic and Barents/White sea phylogeographic groups with *SNPRelate* (Zheng et al. 2012).

### Data preparation

In this study, we focus on genomic regions containing the 116 candidate loci for age at maturity identified in Sinclair-Waters et al. (2020) using an Atlantic salmon aquaculture strain. This strain is derived from founder individuals originating from 41 wild Norwegian rivers, some of which were the same rivers sampled for this study, and all belonging to the same phylogeographic lineage as the sequenced individuals used in this study (Gjedrem et al. 1991). We extracted SNP genotype data from 500 kb regions surrounding the 116 trait-associated SNPs identified in Sinclair-Waters et al. (2020) using *vcftools* (Danecek et al. 2011) position filtering functions –*from-bp* and –*to-bp*, as well as allele filtering function –*mac 1* to keep only polymorphic sites. Trait-associated SNPs that were within 250 kb of another trait-associated SNP were combined into a single candidate region that extends 250 kb upstream of the more upstream SNP to 250 kb downstream of the more downstream SNP.

The current Atlantic salmon genome (ICSASG_v2) contains a known assembly error within the 500 kb region surrounding the known candidate loci *vgll3* (Ayllon et al. 2015). A misplaced and misoriented scaffold currently placed downstream of *vgll3* belongs within a gap in the assembly just upstream of *vgll3* on ssa25. For this reason, we constructed a revised assembly for this chromosome. SNP calling was performed as described above. We then retained SNPs that had met the filtering criteria. A total of 8 candidate SNPs are located within regions of the genome that were moved. To find the position of these SNPs in the revised chromosome 25 sequence, we extracted 200 bp surrounding each of these SNPs from the current genome assembly (ICSASG_v2) using the *getfasta* function in *BEDTools* (Quinlan and Hall 2010). The 200 bp sequence was then blasted to the fixed assembly to determine the new position of each SNP using Blast’s *blastn* function (Camacho et al. 2009). Using the new SNP positions, SNP genotypes within a 500 kb region surrounding the moved candidate SNPs were extracted from the fixed dataset using *vcftools*.

### Association testing at candidate regions

We applied three association mapping methods to describe the genetic architecture underlying sea age at maturity at each of the candidate regions identified in Sinclair-Waters et al. (2020). First, a multi-SNP approach examining associations between phenotype and haplotypes was conducted using Bayesian linear regression implemented in *hapQTLv1.00* (Xu and Guan 2014). In this approach, a hidden Markov model is used to characterize haplotype structure and ancestry (Guan 2014). Haplotype sharing at each marker is then used to quantify genetic similarity among individuals. Haplotype associations are identified by testing for an association between genetic similarity at each marker and the phenotype (Xu and Guan 2014). Each of the extracted *vcf* files was converted to *bimbam* format using *PLINK 1.9* (Chang et al. 2015). The resulting *bimbam* files were used as input for *hapQTL*. Second, single SNP associations were also identified using a Bayesian linear regression method implemented in *hapQTL* (Guan and Stephens 2011). For all *hapQTL* association tests, sex and the six most significant principal components (see above) were included as covariates in the models. Each *hapQTL* run consisted of 2 EM runs (-e 2) with 40 steps (-w 40), 2 upper clusters (-C 2), 10 lower clusters (-c 10). Three replicate *hapQTL* runs were performed for each of the 116 selected regions. Based on recommendations from Jeffreys (1961), Bayes factors greater than three were considered evidence for an association of either SNPs or haplotype with sea age at maturity phenotype.

Third, a multi-SNP approach aimed to estimate the number and identity of SNPs underlying trait variation at each candidate region using Bayesian Variable Selection regression implemented in *PiMASS* (Guan and Stephens 2011). Due to computational restrictions, the *PiMASS* analysis was performed for only candidate regions that had a SNP or haplotype association with Bayes factor greater than 3. Prior to the *PiMASS* analysis, all missing genotypes were imputed in BIMBAM (Guan and Stephens 2011) as mean genotypes (-wmg) using default settings. Additionally, our phenotype values for sea age at maturity were adjusted to correct for confounding effects of sex and population structure by regressing the phenotype on sex and the six most significant principal components (see above) using the *lm* function in *R*. *PiMASS* was run with the residual phenotype values. We placed priors on the proportion of variance explained by SNP(s) (hmin = 0.001 and hmax = 0.999) and the number of SNPs in the model (pmin = log$\frac{1}{N}$ and pmax = log$\frac{{300}}{N}$, where N is the total number of SNPs). Each run consisted of a burn-in of 1000000 steps, followed by 2500000 steps where parameter values were recorded every 1000 steps. For each analysis, we examined the posterior inclusion probability for each SNP, the distribution of the number of included SNPs and the distribution of the proportions of variance explained per model. We also examined the path of estimated Bayes factors and parameter values (h, p, s) across all recorded iterations to check for convergence of runs.

To further assess whether more than one SNP in a candidate region was significantly associated with sea age at maturity, we regressed out the top-associated SNP from the residual phenotype values described above and reran *PiMASS* using the previously-used priors and settings. We then examined the posterior inclusion probability for each SNP, the distribution of the number of included SNPs, and the distribution of proportion of variance explained to determine whether there was evidence for multiple SNP associations within a given candidate region.

## Results

### Analysis of principal components and genetic differentiation

The first six principal components (PCs) calculated with the pruned SNP dataset explained 1.96%, 0.68%, 0.63%, 0.59%, 0.56% and 0.51% of the genetic variance, respectively (Supplementary Fig. S2). These six PCs were included in subsequent association analyses to reflect population structure among samples including structure between phylogeographic groups, structuring occurring along a north-south gradient and other finer scale structuring (Supplementary Fig. S2). We do not include any PCs explaining 0.5% or less of the genetic variance. *F*_ST_ between the Atlantic and Barents/White sea phylogeographic groups was 0.02.

### Associations identified with hapQTL

**Fig. 1:**
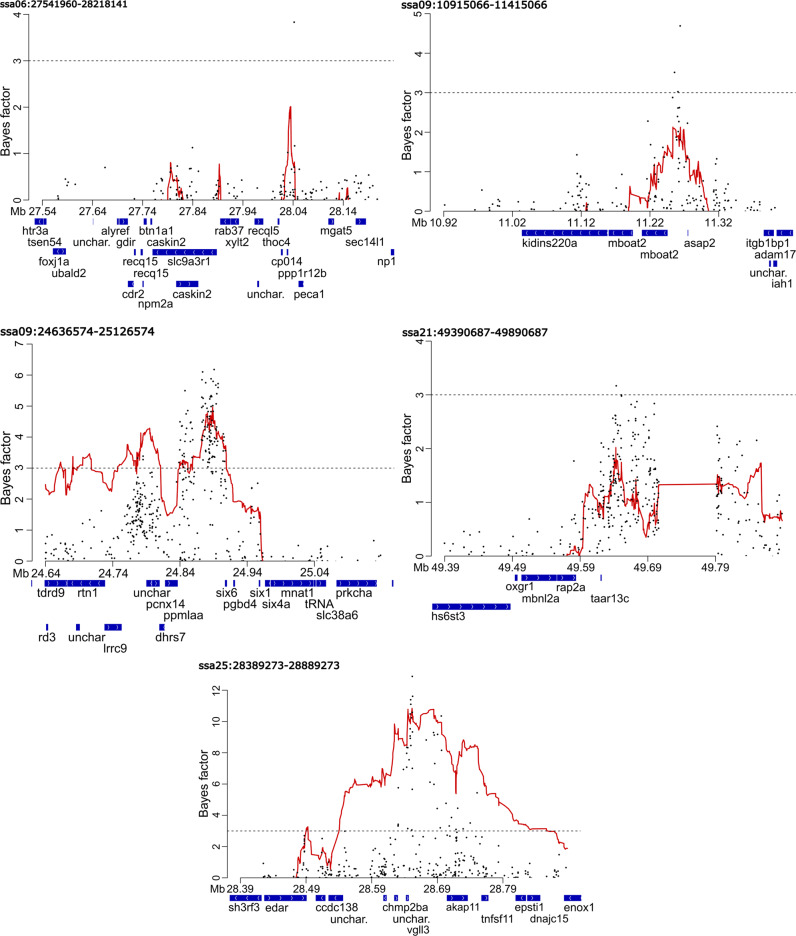
Plots displaying single SNP associations (black points) and haplotype associations (red line) scores from *hapQTL* for the five candidate regions with Bayes factors greater than 3. Y-axis shows the Bayes factor indicating the association strength. X-axis shows the position on the respective chromosomes.

Single-SNP and haplotype association analyses with *hapQTL* revealed strong (Bayes factor > 3) association signals at 5 of the 116 candidate regions (Fig. 1, Supplementary Fig. S3). The strongest association observed within each region was with a single SNP, rather than an extended haplotype, suggesting a single mutation underlies the effect of each of these regions on maturation timing. However, exceptions occurred in the ssa09:24636574-25136574 and ssa25:28389273-28889273 regions, where second association signals were found upstream of the primary association signal and were most strongly linked to an extended haplotype. For instance, strong haplotype association scores (Bayes factor > 3) spanned a 26971 bp region (ssa09:24781742-24808713) containing an uncharacterized gene (LOC106610978) and *pcnx4*. In the ssa25:28389273-28889273 region, a strong haplotype signal was found within *edar* (Fig. 1).

We find differences in the location of the top-associated SNPs found here and those identified in Sinclair-Waters et al. (2020). For regions ssa06:27541960-28218141, ssa09:10915066-11415066 and ssa25:28389273-28889273, the top-associated SNP was located further upstream than in Sinclair-Waters et al. (2020). Contrastingly, the strongest associated SNPs within the regions ssa09:24636574-25136574 and ssa21:49390687-49890687 differed only slightly (<5000 bp) between studies (Table 1).

**Table 1:** Strongest association signals for each candidate region showing evidence of an association with sea age at maturity, the genes in closest proximity and association values from *hapQTL*.

Candidate region	Top signal	Closest gene	Bayes Factor	-log_10_(*P*-value)	Allele frequency	Top SNP(s)^a^	Candidate gene(s)^a^
ssa06:27541960-28218141	6:28045390 (SNP)	*pecam1* (intron)	3.835	5.107	0.320	6:27791960 6:27968141	*slc9a3r1 recql5* LOC106606978
ssa09:10915066-11415066	9:11266848 (SNP)	*asap2a* (upstream)	4.696	5.434	0.074	9:11165066	*mboat2*
ssa09:24636574-25136574	9:24888841 (SNP)	*six6* (upstream)	6.184	4.242	0.425	9:24886574	*six6*
ssa21:49390687-49890687	21:49645222 (SNP)	*taar13c* (upstream)	3.172	4.649	0.464	21:49640687	*taar13c*
ssa25:28389273-28889273	25: 28651640 (SNP) [ICSASG_v2: 25:28669350]	*vgll3* (downstream)	12.893	6.406	0.358	25:28910202	*vgll3*

Top SNPs for each region from previous SNP-array study (Sinclair-Waters et al. 2020).

^a^From Sinclair-Waters et al. (2020).

### Multi-SNP associations identified using PiMASS

**Fig. 2:**
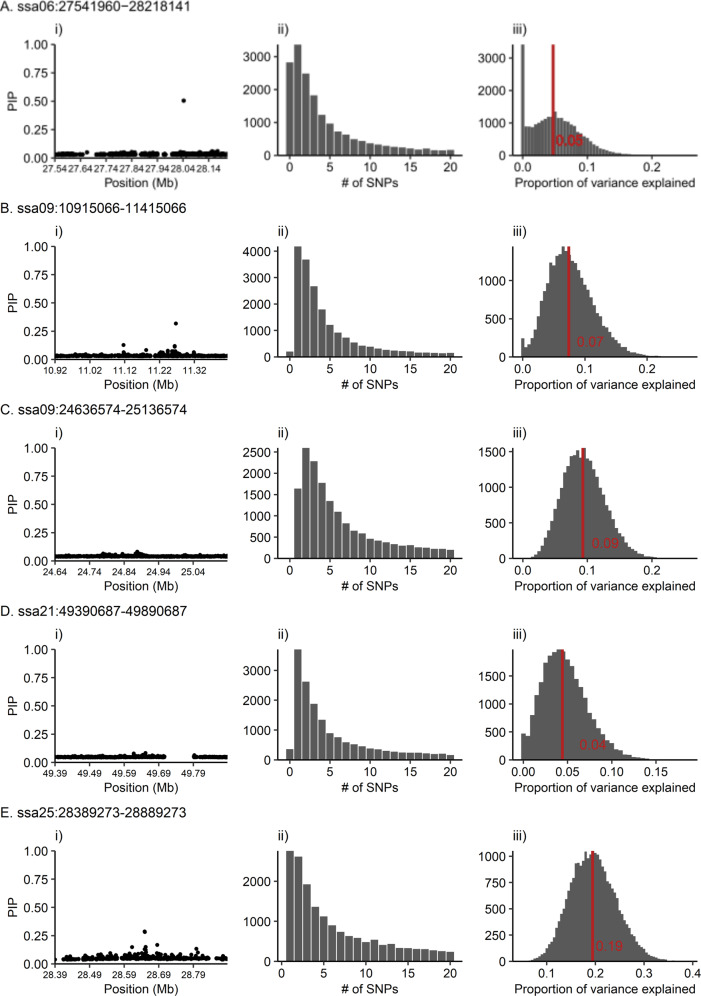
*PiMASS* results for each of the tested candidate regions. **A** ssa06:27541960-28218141, **B** ssa09:10915066-11415066, **C** ssa09:24636574-25136574, **D** ssa21:49390687-49890687, and **E** ssa25:28389273-28889273. Plots display the following results for each candidate region: (i) posterior inclusion probability (PIP) indicating the probability of a SNP being included in a model explaining sea age at maturity variation, (ii) truncated distribution of the number of SNPs included in a model explaining sea age at maturity variation, and (iii) distribution of proportion of variance explained per recorded iteration (2500). Red line indicates the median proportion of variance explained.

Multi-SNP association analysis with *PiMASS* showed that at four of five candidate regions, a single-SNP model was most commonly used to explain variation in sea age at maturity. At one candidate region, ssa09:24636574-25136574, a multi-SNP model including two SNPs was most commonly used to explain variation in sea age at maturity. Median proportion of variance explained by each candidate region ranged between 4% and 19% (Fig. 2, Table 2). Additionally, mean sea age at maturity differed substantially among genotypes at all six SNPs selected by the multi-SNP models (Supplementary Fig. S4). However, when the top-associated SNP was regressed out from the phenotype values, no SNPs were selected to explain sea age at maturity for all five candidate regions. Additionally, post-regression median proportion of variance was substantially lower—ranging between 0% and 1% (Supplementary Fig. S5, Table 2). This would suggest that sea age variation explained by each of these regions is largely explained by a single mutation. Pairwise LD among SNPs (*R*^2^) in these regions are reported in Supplementary Fig. S6. We observe no obvious trends in parameter values or Bayes factors, suggesting models converged and burn-in period was adequate (Supplementary Figs. S7 and S8).

**Table 2:** *PiMASS* results prior to and after regression of top-associated SNP identified in the initial *PiMASS* analysis.

Candidate region	Mode # of SNPs	Median PVE	Mode # of SNPs (post-regression)	Median PVE (post-regression)
ssa06:27541960-28218141	1	0.05	0	0
ssa09:10915066-11415066	1	0.07	0	0.01
ssa09:24636574-25136574	2	0.09	0	0.01
ssa21:49390687-49890687	1	0.04	0	0
ssa25:28389273-28889273	1	0.19	0	0.01

These include the mode of the distribution of the number of SNPs and the median of the distribution of proportion of variance explained (PVE) for a model explaining sea age at maturity.

## Discussion

Despite that combined effects of multiple variants at trait-associated loci are playing an important role in controlling fitness traits across a variety of species (Linnen et al. 2013; Bickel et al. 2011; Kerdaffrec et al. 2016), our results indicate that sea age at maturation in Atlantic salmon is predominantly associated with single SNP variation at candidate regions. Using resequencing data to analyse 116 candidate loci and an analytical framework aimed at detecting multi-SNP associations, we find that single SNPs explain the variation in sea age at maturity in almost all cases. This work targeting candidate genes identified in aquaculture salmon strains suggests a mixed genetic architecture where a combination large-effect loci and smaller-effect loci also underlies age at maturity in wild Atlantic salmon populations. Two core loci, *vgll3* and *six6*, likely play a key role in determining age at maturity and additional smaller effect loci may be important for fine-tuning the trait across heterogeneous environments.

Theoretical modelling predicts that clustering of tightly linked adaptive mutations will occur under gene flow and selection in populations inhabiting spatially and/or temporally heterogeneous environments (Yeaman and Whitlock 2011; Yeaman 2013). In Atlantic salmon, mean sea age at maturity varies among populations. Furthermore, spatially varying selection at the *vgll3* locus displays homozygosity patterns consistent with selection towards local optima for sea age at maturity (Barson et al. 2015). Although these theoretical predictions seem to be a plausible scenario under which the genetic architecture of age at maturity has evolved in Atlantic salmon, our work suggests that the association at four of the five candidate regions is driven by a single mutation. We cannot rule out, however, the possibility that the examined regions have pleiotropic effects and contain SNPs controlling other adaptive traits that have weak or no correlation with maturation timing. It is also possible that we did not have sufficient power to detect additional SNPs in these regions with small effects or with rare alleles. However, previous empirical studies have found few, but complex, loci with clusters of adaptive mutations (Kerdaffrec et al. 2016; Linnen et al. 2013; Bickel et al. 2011), thus motivating our investigation of multi-SNP and haplotypic effects. Remington (2015) also highlights the importance of distinguishing between allelic effects and single mutational effects when examining the genetic architecture of adaptive variation and its evolution. Our findings, however, suggest that alternative genetic architectures are feasible. One possible explanation could relate to the multiple whole genome duplication events that have occurred in Atlantic salmon and other salmonids (Allendorf and Thorgaard 1984). The presence of multiple gene copies may impact the evolution of genetic architecture for traits such as age at maturity in Atlantic salmon. It is also possible that gene flow among Atlantic salmon populations is too restricted to neighbouring populations and/or strength of selection is insufficient for the establishment of linked mutations, as there is a rather specific balance of gene flow and selection required for clustered loci to arise (Yeaman et al. 2016). Both an extension of models predicting genetic architecture and additional empirical studies—on a wider variety organisms and traits—are needed to evaluate the generality of particular architectures and to further understand the conditions under which they evolve.

We find additional evidence that a large-effect locus on ssa25, *vgll3*, largely underlies age at maturity in Atlantic salmon corroborating findings from a number of association studies on Atlantic salmon maturation (Barson et al. 2015; Ayllon et al. 2015; Ayllon et al. 2019; Sinclair-Waters et al. 2020; Sinclair-Waters et al. 2022). The second strongest associated locus in this study is located in close proximity to *six6* on ssa09. This locus was previously found to be associated with early maturation in male farmed Atlantic salmon (Sinclair-Waters et al. 2020), with sea age at maturity in wild Atlantic salmon prior to population structure correction (Barson et al. 2015) and two species of Pacific salmon (Sockeye salmon and Steelhead trout). Although *six6* is associated with maturation in both Atlantic and several Pacific salmon species, an association between *vgll3* and maturation timing has not been found in Pacific salmon species (Waters et al. 2021; Willis et al. 2020). Additionally, we found another three loci associated with sea age at maturity: *pecam1, asap2aa* and *taar13c*. The handful of loci found here suggests that wild Atlantic salmon have a mixed genetic architecture where multiple loci, with a variety of effect sizes, control maturation timing—similar to what has been found in male farmed Atlantic salmon (Sinclair-Waters et al. 2020). Knowledge of this mixed genetic architecture is highly relevant for how we predict the evolution of maturation timing in wild Atlantic salmon populations. A large body of work has shown the relevance of genetic architecture in determining evolutionary responses (Barton and Turelli 1991; Turelli 1984; Turelli and Barton 2004; Turelli and Barton 1990; Lande 1975; Bulmer 1972; Débarre et al. 2015; Fisher 1930; Yeaman 2015). Recent works highlight the relevance of the genetic architecture underlying fitness traits when predicting a population’s response to environmental changes (Kardos and Luikart 2021) and selective pressures such a fishing (Oomen et al. 2020). Future work elucidating how such mixed genetic architectures affect predicted evolution of traits, compared to that of omnigenic or polygenic architectures, will be valuable.

We find differences in locations of top-associated SNPs identified here and in Sinclair-Waters et al. (2020). This is not surprising given that we are examining sequence data that captures additional SNP variation in regions surrounding SNPs included in the SNP-array used in Sinclair-Waters et al. (2020). Furthermore, we failed to find associations between sea age at maturity and many of the candidate regions identified in Sinclair-Waters et al. (2020). For example, several candidate regions on ssa03 and ssa04 displayed particularly strong association signals in aquaculture salmon, however, no signals at these regions were found here. Additionally, only one association peak at ssa06:27541960-28218141 was found here, whereas two independent associations within this region were found in aquaculture salmon (Sinclair-Waters et al. 2020). Such differences may reflect changes in the genetic architecture of the trait evolving since the domestication of Atlantic salmon. Although, we would not expect large changes to occur given the domestication is relatively recent, just 10 to 15 generations ago (Gjerde and Gjedrem 1984). Furthermore, this study is likely under-powered to detect all previously identified loci, particularly those with smaller effect sizes or rare alleles, due to the smaller sample size of 313 individuals. Additionally, there could be differences in genetic architecture among environments (Yan et al. 2021) and/or genotype by environment interactions giving rise to distinct genetic architectures in wild populations versus aquaculture strains.

We do not find strong evidence of multi-SNP associations at candidate loci examined in this study, however, we cannot yet disregard the utility of multi-SNP association methods for further resolving the genetic architecture of Atlantic salmon maturation. First, we do not examine the entire genome due to computational restrictions, rather, we focussed on 116 previously identified candidate regions. Second, the Atlantic salmon genome is highly complex (Lien et al. 2016) and therefore errors in the assembly that may be disruptive for haplotype-based analysis could exist. As new and improved versions of the Atlantic salmon genome are published, our ability to test for haplotypic associations will improve. Furthermore, in a few cases (ssa09:10915066-11415066, ssa09:24636574-25136574, ssa25:28389273-28889273) the *PiMASS* analyses post-regression of the top SNP selected no SNPs for a model explaining sea age at maturity variation, however, the median proportion of variance explained across all iterations was greater than zero. This may suggest that a weak signal was present, but was being missed due to insufficient power. Although this is largely speculative, it suggests that ruling out the possibility of multi-SNP associations at these particular candidate regions may be premature. Higher-powered studies (i.e. more individuals per population) may help to resolve this in the future. Additionally, if an additional SNP is in high LD with the top-associated SNP, disentangling its effects from the top-associated SNP is challenging and its association signal could be undetectable post-regression of the top SNP. In such cases, study designs that take advantage of recombination events between highly linked SNPs are useful for characterizing genetic architectures at finer-scales (Sinclair-Waters et al. 2022).

Our analytical framework, combining both single and multi-SNP association methods, reveals that single SNP variation is sufficient for explaining the association at multiple previously identified candidate loci for Atlantic salmon maturation timing. Previous empirical and theoretical work have described trait-associated loci that have complex alleles with multiple variants, our findings therefore demonstrate the diversity of genetic architectures for fitness-related traits. Additional data, and a greater diversity of species and traits, will serve to better understand why this diversity of genetic architectures exists and how these particular genetic architectures evolve. The analytical framework used here will be a valuable resource for accomplishing this as individual-level resequencing data for wild species with phenotyped individuals becomes increasingly available.

## Supplementary information

Supplementary Table S1

Supplementary Table S2

Supplementary Figures

## Data Availability

Genome re-sequencing data for individuals used in this study are available in the European Nucleotide Archive (ENA) or NCBI with the project accession code PRJEB38061 (Bertolotti et al. 2020).
