# Supplementary material for: Dissecting the loci underlying maturation timing in Atlantic salmon using haplotype and multi-SNP based association methods
Source: Heredity (Edinb). 2022 Nov 10;129(6):356–65. doi: 10.1038/s41437-022-00570-w (PMC9709158; doi:10.1038/s41437-022-00570-w)
Supplement: Supplementary file 1 — Supplementary Table S1 [file 41437_2022_570_MOESM1_ESM.pdf]

Supplementary Table S1. List of river names, river ID, latitude and longitude coordinates, and number of individuals (N) collected at each location.

River	River ID	Latitude	Longitude	N
Alta	212.Z	69.968	23.375	10
Årgårdsvassdraget	138.Z	64.312	11.223	10
Årøy	077.Z	61.268	7.1671	2
Beiarvassdraget	161.Z	67.028	14.579	2
Børselva in Porsanger	225.Z	70.312	25.539	2
Daleelva (Høyangervassdraget)	079.Z	61.219	6.0748	2
Driva	109.Z	62.67606	8.550612	8
Eidfjordvassdraget	050.Z	60.466	7.0718	2
Eira	104.Z	62.678	8.1196	2
Elvegårdselva (Bjerkvik)	175.Z	68.546	17.562	1
Enningdalselva	001.1Z	58.981	11.474	10
Ervikelva	091.3Z	68.8218	16.48854	10
Etneelva	041.Z	59.67	5.9416	2
Flåmselva	072.2Z	60.8651	7.1186	10
Flekkeelva	082.Z	61.31	5.3445	2
Forsåvassdraget	172.Z	68.151	16.116	2
Gaula in Sør-Trøndelag	122.Z	63.341	10.236	2
Gloppenelva	087.Z	61.768	6.2	2
Homla	123.4Z	63.413	10.804	10
Jølstra	084.Z	61.455	5.8434	3
Komagelva	239.Z	70.242	30.522	10
Lærdalselva	073.Z	61.102	7.4725	2
Tana (Laksjohka)	234.Z	70.059	27.562	3
Lakselva in Porsanger	224.Z	70.078	24.927	3
Langfjordelva	233.Z	70.66914	27.83905	10
Laukhellevassdraget (Lakselva from Trollbuvatnet)	194.Z	69.227	17.849	10
Loneelva in Osterøy	060.4Z	60.52	5.5011	2
Målselvvassdraget	196.Z	69.264	18.51	9
Tana (Maskejohka)	234.Z	70.285	28.163	3
Namsen (hele vassdraget)	139.Z	64.464	11.682	5
Namsen (Fiskumfoss/Tørris)	139Z	64.60584	12.54821	5
Nausta	084.7Z	61.506	5.7197	10
Neiden (Näätämöjoki in Finnish)	244.Z	69.70143	29.52523	10
Numedalslågen	015.Z	59.06	10.071	10
Orkla	121.Z	63.30663	9.82683	10
Oselva in Os	085.Z	60.186	5.4723	2
Oselva in Osen	055.7Z	61.55063	5.413458	10
Reipåga	160.43Z	66.908	13.632	10
Repparfjordelva	213.Z	70.445	24.328	10
Risfjordvassdraget	231.8Z	70.978	28.171	3
Roksdalsvassdraget	186.2Z	69.05	15.869	2
Ryggelva	087.1Z	61.779	6.1249	10
Saltdalsvassdraget	163.Z	67.098	15.419	10
Sandfjordelva in Gamvik	231.7Z	71.049	28.057	3

Skienselva	016.Z	59.135	9.6301	2
Skipsfjordvassdraget	202.11Z	70.158	19.797	2
Suldalslågen	036.Z	59.48	6.2506	10
Surna	112.Z	62.971	8.6624	2
Sylteelva in Fræna	107.3Z	62.838	7.2096	2
Teno (lower-Utsjoki)	234.Z	69.909	27.0285	14
Vestre Jakobselv	240.Z	70.108	29.327	3
Vigda	122.2Z	63.312	10.182	10
Vikedalselva in Vindafjord	038.Z	59.496	5.8972	10
Vorma	035.3Z	59.271	6.3322	2
