# Supplementary material for: Dissecting the loci underlying maturation timing in Atlantic salmon using haplotype and multi-SNP based association methods
Source: Heredity (Edinb). 2022 Nov 10;129(6):356–65. doi: 10.1038/s41437-022-00570-w (PMC9709158; doi:10.1038/s41437-022-00570-w)
Supplement: Supplementary file 2 — Supplementary Table S2 [file 41437_2022_570_MOESM2_ESM.pdf]

Supplementary Table S2. List of individual sample names and their accession numbers (ENA).

| Sample name | Accession |
|---|---|
| Alta_12_0001 | ERS840243 |
| Alta_12_0038 | ERS4601683 |
| Alta_12_0111 | ERS4601684 |
| Alta_12_0124 | ERS840244 |
| Alta_12_0135 | ERS4601685 |
| Alta_12_0179 | ERS4601686 |
| Alta_12_0190 | ERS4601687 |
| Alta_12_0201 | ERS4601688 |
| Alta_12_0217 | ERS4601689 |
| Alta_12_0228 | ERS840245 |
| Arga_12_0015 | ERS4601690 |
| Arga_12_0075 | ERS840258 |
| Arga_12_0082 | ERS840259 |
| Arga_12_0089 | ERS840260 |
| Arga_12_0121 | ERS4601691 |
| Arga_15_0031 | ERS4601692 |
| Arga_15_0033 | ERS4601693 |
| Arga_15_0049 | ERS4601694 |
| Arga_15_0062 | ERS4601695 |
| Arga_15_0201 | ERS4601696 |
| Aroy_12_0073 | ERS4601697 |
| Aroy_12_0084 | ERS4601698 |
| Beia_12_0009 | ERS4601707 |
| Beia_12_0281 | ERS4601708 |
| Bors_11_0008 | ERS4601719 |
| Bors_11_0027 | ERS4601720 |
| Dale_12_0053 | ERS4601731 |
| Dale_12_0065 | ERS4601732 |
| Driv_12_0056 | ERS4601733 |
| Driv_12_0085 | ERS4601734 |
| Driv_12_0089 | ERS4601735 |
| Driv_12_0100 | ERS4601736 |
| Driv_12_0131 | ERS4601737 |
| Driv_12_0144 | ERS4601738 |
| Driv_12_0146 | ERS4601739 |
| Driv_12_0302 | ERS4601740 |
| Eidf_12_0071 | ERS4601741 |

| | |
|---|---|
| Eidf_12_0076 | ERS4601742 |
| Eira_12_0091 | ERS4601743 |
| Eira_12_0110 | ERS4601744 |
| Elve_12_0011 | ERS4601753 |
| Enni_12_0008 | ERS4601754 |
| Enni_12_0032 | ERS4601755 |
| Enni_12_0051 | ERS4601756 |
| Enni_12_0064 | ERS4601757 |
| Enni_12_0085 | ERS4601758 |
| Enni_12_0091 | ERS4601759 |
| Enni_12_0092 | ERS4601760 |
| Enni_12_0094 | ERS4601761 |
| Enni_12_0102 | ERS4601762 |
| Enni_12_0105 | ERS4601763 |
| Ervi_03_0002 | ERS4601764 |
| Ervi_03_0003 | ERS4601765 |
| Ervi_03_0004 | ERS4601766 |
| Ervi_03_0006 | ERS4601767 |
| Ervi_03_0008 | ERS4601768 |
| Ervi_03_0010 | ERS4601769 |
| Ervi_03_0016 | ERS4601770 |
| Ervi_03_0017 | ERS4601771 |
| Ervi_03_0018 | ERS4601772 |
| Ervi_03_0021 | ERS4601773 |
| Etne_12_0023 | ERS4601774 |
| Etne_12_0371 | ERS4601775 |
| Flam-15_0002 | ERS4601776 |
| Flam-15_0007 | ERS4601777 |
| Flam-15_0008 | ERS4601778 |
| Flam-15_0009 | ERS4601779 |
| Flam-15_0010 | ERS4601780 |
| Flam-15_0014 | ERS4601781 |
| Flam-15_0015 | ERS4601782 |
| Flam-15_0017 | ERS4601783 |
| Flam-15_0027 | ERS4601784 |
| Flam-15_0031 | ERS4601785 |
| Flek_11_0132 | ERS4601786 |
| Flek_11_0244 | ERS4601787 |
| Fors_12_0025 | ERS4601788 |

| | |
|---|---|
| Fors_12_0038 | ERS4601789 |
| GauST_13_0058 | ERS4601790 |
| GauST_13_0075 | ERS4601791 |
| Glop_11_0001 | ERS4601800 |
| Glop_11_0216 | ERS4601801 |
| Homl_11_0001 | ERS4601802 |
| Homl_12_0001 | ERS4601803 |
| Homl_12_0002 | ERS4601804 |
| Homl_12_0003 | ERS4601805 |
| Homl_12_0004 | ERS4601806 |
| Homl_12_0008 | ERS4601807 |
| Homl_14_0001 | ERS4601808 |
| Homl_14_0002 | ERS4601809 |
| Homl_14_0005 | ERS4601810 |
| Homl_14_0006 | ERS4601811 |
| Jols_13_0001 | ERS840250 |
| Jols_13_0009 | ERS840249 |
| Jols_13_0027 | ERS840251 |
| Koma_06_0007 | ERS4601833 |
| Koma_06_0009 | ERS4601834 |
| Koma_06_0010 | ERS4601835 |
| Koma_06_0012 | ERS4601836 |
| Koma_07_0001 | ERS4601837 |
| Koma_07_0002 | ERS4601838 |
| Koma_07_0010 | ERS4601839 |
| Koma_07_0015 | ERS4601840 |
| Koma_12_0013 | ERS4601841 |
| Koma_12_0110 | ERS4601842 |
| Laer_07_0001 | ERS4601853 |
| Laer_07_0016 | ERS4601854 |
| Lakj_03_0009 | ERS4601855 |
| Lakj_10_0312 | ERS4601856 |
| Lakj_10_0318 | ERS4601857 |
| Laks_12_0006 | ERS4601858 |
| Laks_12_0021 | ERS4601859 |
| Laks_12_0106 | ERS4601860 |
| LanG_12_0007 | ERS4601861 |
| LanG_12_0012 | ERS4601862 |
| LanG_12_0013 | ERS4601863 |

| | |
|---|---|
| LanG_12_0020 | ERS4601864 |
| LanG_12_0025 | ERS4601865 |
| LanG_12_0028 | ERS4601866 |
| LanG_12_0031 | ERS4601867 |
| LanG_12_0032 | ERS4601868 |
| LanG_12_0036 | ERS4601869 |
| LanG_12_0042 | ERS4601870 |
| Lauk_12_0011 | ERS4601871 |
| Lauk_12_0014 | ERS4601872 |
| Lauk_13_0013 | ERS4601873 |
| Lauk_13_0016 | ERS4601874 |
| Lauk_13_0017 | ERS4601875 |
| Lauk_13_0019 | ERS4601876 |
| Lauk_13_0025 | ERS4601877 |
| Lauk_13_0031 | ERS4601878 |
| Lauk_13_0039 | ERS4601879 |
| Lauk_13_0045 | ERS4601880 |
| Lone_12_0018 | ERS4601881 |
| Lone_12_0023 | ERS4601882 |
| Mals_11_0025 | ERS4601893 |
| Mals_12_0020 | ERS4601894 |
| Mals_12_0022 | ERS4601895 |
| Mals_13_S_0004 | ERS4601896 |
| Mals_13_S_0007 | ERS4601897 |
| Mals_13_S_0054 | ERS4601898 |
| Mals_13_S_0059 | ERS4601899 |
| Mals_13_S_0063 | ERS4601900 |
| Mals_13_S_0067 | ERS4601901 |
| Mals_13_S_0105 | ERS4601902 |
| Mask_06_0199 | ERS4601903 |
| Mask_06_0200 | ERS4601904 |
| Mask_06_0212 | ERS4601905 |
| Nams_12_0024 | ERS840256 |
| Nams_12_0049 | ERS4601906 |
| Nams_12_0071 | ERS840257 |
| Nams_12_0201 | ERS840255 |
| Nams_12_0267 | ERS4601907 |
| NamsF_14_H_0286 | ERS4601923 |
| NamsF_14_H_0287 | ERS4601924 |

| | |
|---|---|
| NamsF_14_H_0291 | ERS4601925 |
| NamsF_14_H_0302 | ERS4601926 |
| NamsF_14_H_0314 | ERS4601927 |
| Naus_12_0014 | ERS840253 |
| Naus_12_0016 | ERS4601928 |
| Naus_12_0037 | ERS840252 |
| Naus_12_0038 | ERS4601929 |
| Naus_12_0041 | ERS4601930 |
| Naus_12_0059 | ERS840254 |
| Naus_12_0063 | ERS4601931 |
| Naus_12_0066 | ERS4601932 |
| Naus_12_1501 | ERS4601933 |
| Naus_12_1506 | ERS4601934 |
| Neid_13_1181 | ERS4601935 |
| Neid_13_2888 | ERS4601936 |
| Neid_13_2894 | ERS4601937 |
| Neid_13_2899 | ERS4601938 |
| Neid_13_3063 | ERS4601939 |
| Neid_13_3084 | ERS4601940 |
| Neid_13_3170 | ERS4601941 |
| Neid_13_3178 | ERS4601942 |
| Neid_13_3181 | ERS4601943 |
| Neid_13_7799 | ERS4601944 |
| Nume_12_0015 | ERS4601945 |
| Nume_12_0043 | ERS4601946 |
| Nume_12_0044 | ERS4601947 |
| Nume_12_0051 | ERS4601948 |
| Nume_12_0085 | ERS4601949 |
| Nume_12_0123 | ERS4601950 |
| Nume_12_0232 | ERS4601951 |
| Nume_12_0245 | ERS4601952 |
| Nume_12_0253 | ERS4601953 |
| Nume_12_0267 | ERS4601954 |
| Orkl_12_0804 | ERS4601955 |
| Orkl_12_0850 | ERS4601956 |
| Orkl_12_0885 | ERS4601957 |
| Orkl_12_0899 | ERS4601958 |
| Orkl_12_0916 | ERS4601959 |
| Orkl_12_0941 | ERS4601960 |

| | |
|---|---|
| Orkl_12_0950 | ERS4601961 |
| Orkl_13_0001 | ERS4601962 |
| Orkl_13_0113 | ERS4601963 |
| Orkl_13_0171 | ERS4601964 |
| Osel_11_0044 | ERS4601965 |
| Osel_11_0055 | ERS4601966 |
| Osen_10_0003 | ERS4601967 |
| Osen_10_0015 | ERS4601968 |
| Osen_10_0017 | ERS4601969 |
| Osen_10_0019 | ERS4601970 |
| Osen_10_0023 | ERS4601971 |
| Osen_10_0024 | ERS4601972 |
| Osen_10_0029 | ERS4601973 |
| Osen_10_0030 | ERS4601974 |
| Osen_10_0032 | ERS4601975 |
| Osen_10_0034 | ERS4601976 |
| Reip_11_0005 | ERS4601987 |
| Reip_11_0009 | ERS4601988 |
| Reip_11_0010 | ERS4601989 |
| Reip_11_0011 | ERS4601990 |
| Reip_11_0012 | ERS4601991 |
| Reip_11_0015 | ERS4601992 |
| Reip_12_0005 | ERS4601993 |
| Reip_14_0007 | ERS4601994 |
| Reip_14_0008 | ERS4601995 |
| Reip_14_0011 | ERS4601996 |
| Repp_12_0007 | ERS840246 |
| Repp_12_0008 | ERS4601997 |
| Repp_12_0010 | ERS4601998 |
| Repp_12_0011 | ERS4601999 |
| Repp_12_0019 | ERS4602000 |
| Repp_12_0023 | ERS840247 |
| Repp_12_0029 | ERS4602001 |
| Repp_12_0030 | ERS4602002 |
| Repp_12_0032 | ERS4602003 |
| Repp_12_0034 | ERS840248 |
| Risf_11_0006 | ERS4602004 |
| Risf_11_0011 | ERS4602005 |
| Risf_11_0015 | ERS4602006 |

| | |
|---|---|
| Roks_12_0111 | ERS4602007 |
| Roks_12_0113 | ERS4602008 |
| Rygg_11_0001 | ERS4602009 |
| Rygg_11_0022 | ERS4602010 |
| Rygg_12_0002 | ERS4602011 |
| Rygg_12_0007 | ERS4602012 |
| Rygg_12_0008 | ERS4602013 |
| Rygg_12_0009 | ERS4602014 |
| Rygg_12_0010 | ERS4602015 |
| Rygg_12_0024 | ERS4602016 |
| Rygg_12_0025 | ERS4602017 |
| Rygg_12_0026 | ERS4602018 |
| Salt_12_0036 | ERS4602019 |
| Salt_12_0039 | ERS4602020 |
| Salt_12_0041 | ERS4602021 |
| Salt_12_0067 | ERS4602022 |
| Salt_12_0073 | ERS4602023 |
| Salt_12_0074 | ERS4602024 |
| Salt_12_0119 | ERS4602025 |
| Salt_12_0131 | ERS4602026 |
| Salt_12_0132 | ERS4602027 |
| Salt_12_0174 | ERS4602028 |
| Sand_12_0004 | ERS4602029 |
| Sand_12_0006 | ERS4602030 |
| Sand_12_0038 | ERS4602031 |
| Skie_12_0091 | ERS4602032 |
| Skie_12_0144 | ERS4602033 |
| Skip_12_0014 | ERS4602034 |
| Skip_12_0027 | ERS4602035 |
| Suld_11_0118 | ERS4602054 |
| Suld_11_0169 | ERS4602055 |
| Suld_11_0270 | ERS4602056 |
| Suld_11_0303 | ERS4602057 |
| Suld_11_0356 | ERS4602058 |
| Suld_11_0390 | ERS4602059 |
| Suld_11_0553 | ERS4602060 |
| Suld_11_0593 | ERS4602061 |
| Suld_11_0733 | ERS4602062 |
| Suld_11_0829 | ERS4602063 |

| | |
|---|---|
| Surn_13_0028 | ERS4602064 |
| Surn_13_0280 | ERS4602065 |
| Sylt_12_0055 | ERS4602066 |
| Sylt_12_0103 | ERS4602067 |
| Uts_11_15 | ERS840261 |
| Uts_11_17 | ERS840262 |
| Uts_11_18 | ERS840263 |
| Uts_11_24 | ERS840264 |
| Uts_11_26 | ERS840265 |
| Uts_11_27 | ERS840266 |
| Uts_11_28 | ERS840267 |
| Uts_11_29 | ERS840268 |
| Uts_11_30 | ERS840269 |
| Uts_11_31 | ERS840270 |
| Uts_11_39 | ERS840271 |
| Uts_11_46 | ERS840272 |
| Uts_11_52 | ERS840273 |
| Uts_11_53 | ERS840274 |
| VeJa_13_0112 | ERS4602068 |
| VeJa_13_0132 | ERS4602069 |
| Vigd_09_0001 | ERS4602080 |
| Vigd_09_0004 | ERS4602081 |
| Vigd_09_0006 | ERS4602082 |
| Vigd_09_0007 | ERS4602083 |
| Vigd_09_0013 | ERS4602084 |
| Vigd_09_0018 | ERS4602085 |
| Vigd_09_0020 | ERS4602086 |
| Vigd_09_0021 | ERS4602087 |
| Vigd_09_0022 | ERS4602088 |
| Vigd_09_0027 | ERS4602089 |
| Vike_12_0013 | ERS4602090 |
| Vike_12_0014 | ERS4602091 |
| Vike_13_0005 | ERS4602092 |
| Vike_13_0013 | ERS4602093 |
| Vike_13_0016 | ERS4602094 |
| Vike_13_0038 | ERS4602095 |
| Vike_13_0041 | ERS4602096 |
| Vike_13_0042 | ERS4602097 |
| Vike_14_00011 | ERS4602098 |

| | |
|---|---|
| Vike_14_00020 | ERS4602099 |
| Vorm_11_0035 | ERS4602100 |
| Vorm_11_0039 | ERS4602101 |
