# Supplementary material for: Dissecting the loci underlying maturation timing in Atlantic salmon using haplotype and multi-SNP based association methods
Source: Heredity (Edinb). 2022 Nov 10;129(6):356–65. doi: 10.1038/s41437-022-00570-w (PMC9709158; doi:10.1038/s41437-022-00570-w)
Supplement: Supplementary file 3 — Supplementary Figures [file 41437_2022_570_MOESM3_ESM.docx]

Supplementary Figures

**Supplementary Figure S1. Number of male and female individuals per sea age class within each river.**

**
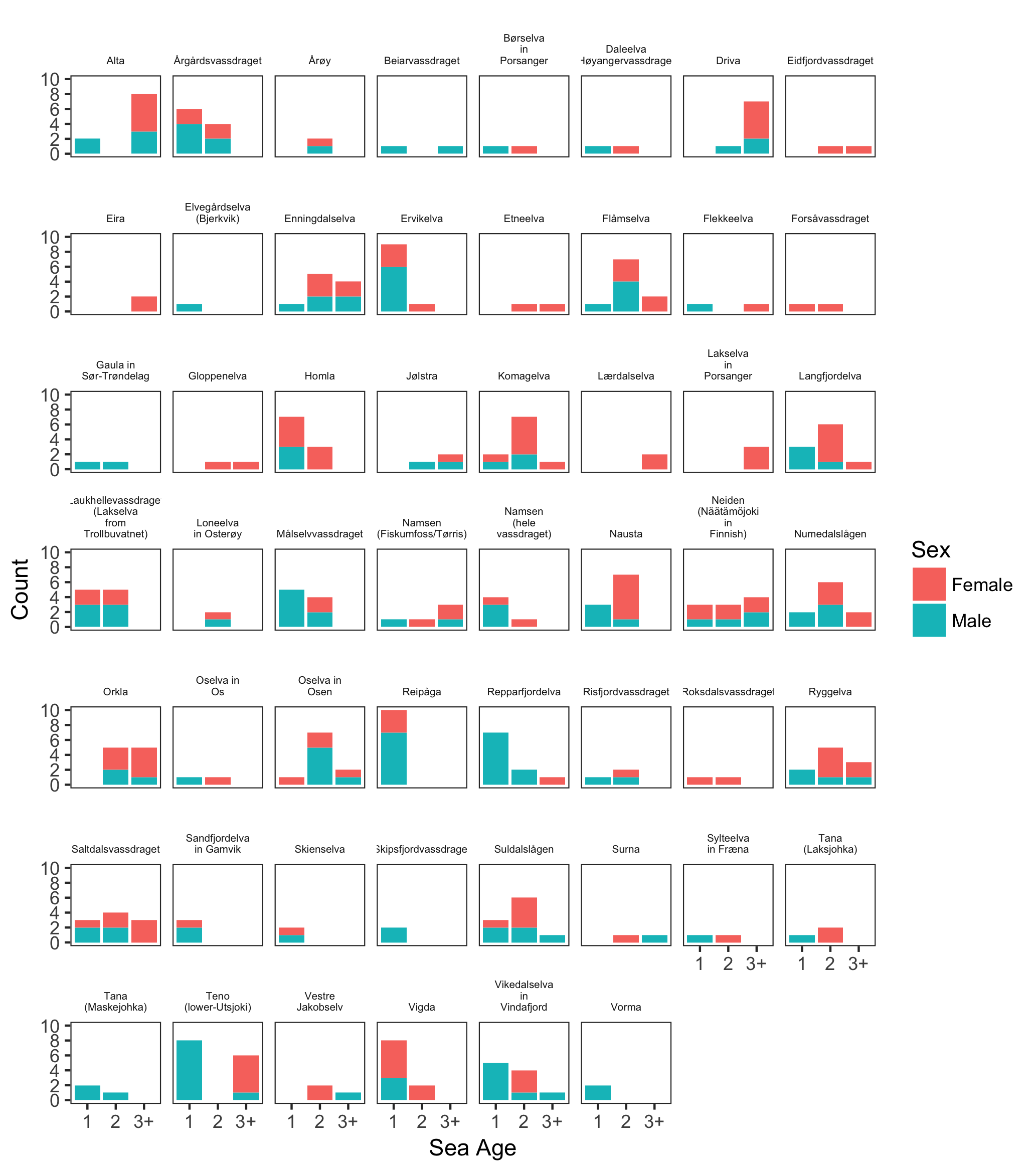
**


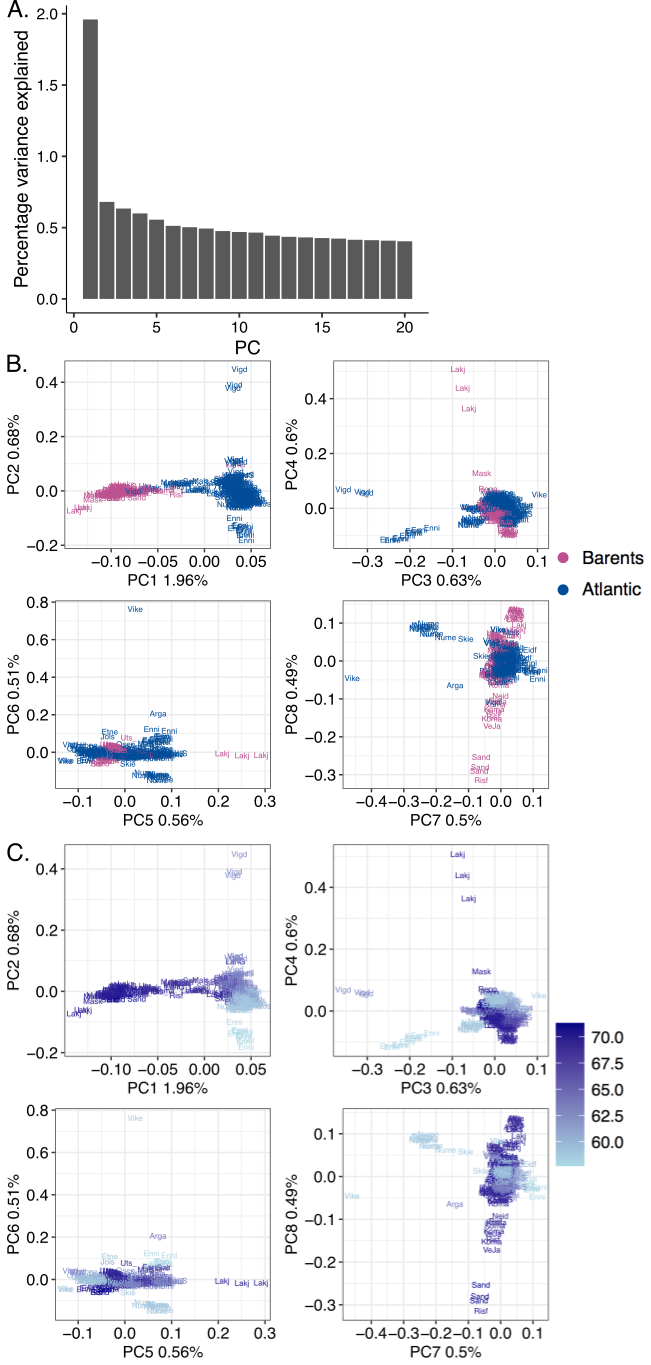


Supplementary Figure S2. A) Percentage of variance explained by the first 20 principal components. B) PCA plots displaying the first eight principal components where samples are coloured according to phylogeographic group. C) PCA plots displaying the first eight principal components where samples are coloured according to latitude.

Supplementary Figure S3. Plots displaying single SNP associations (black points) and haplotype associations (red line) scores from *hapQTL* for each candidate region with Bayes factors less than 3. Y-axis shows the Bayes Factor indicating the degree of association strength. X-axis shows the position on the respective chromosomes.


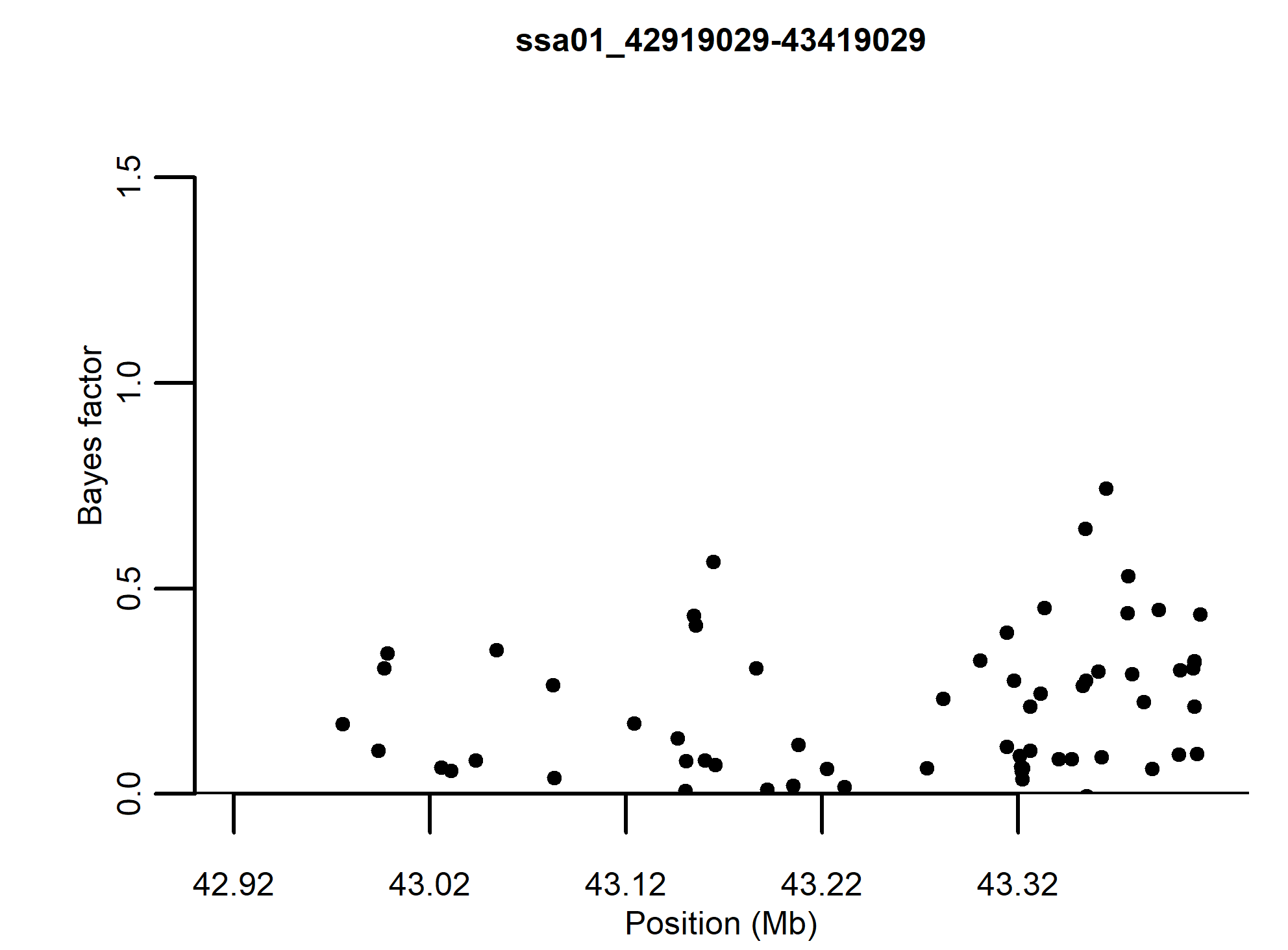

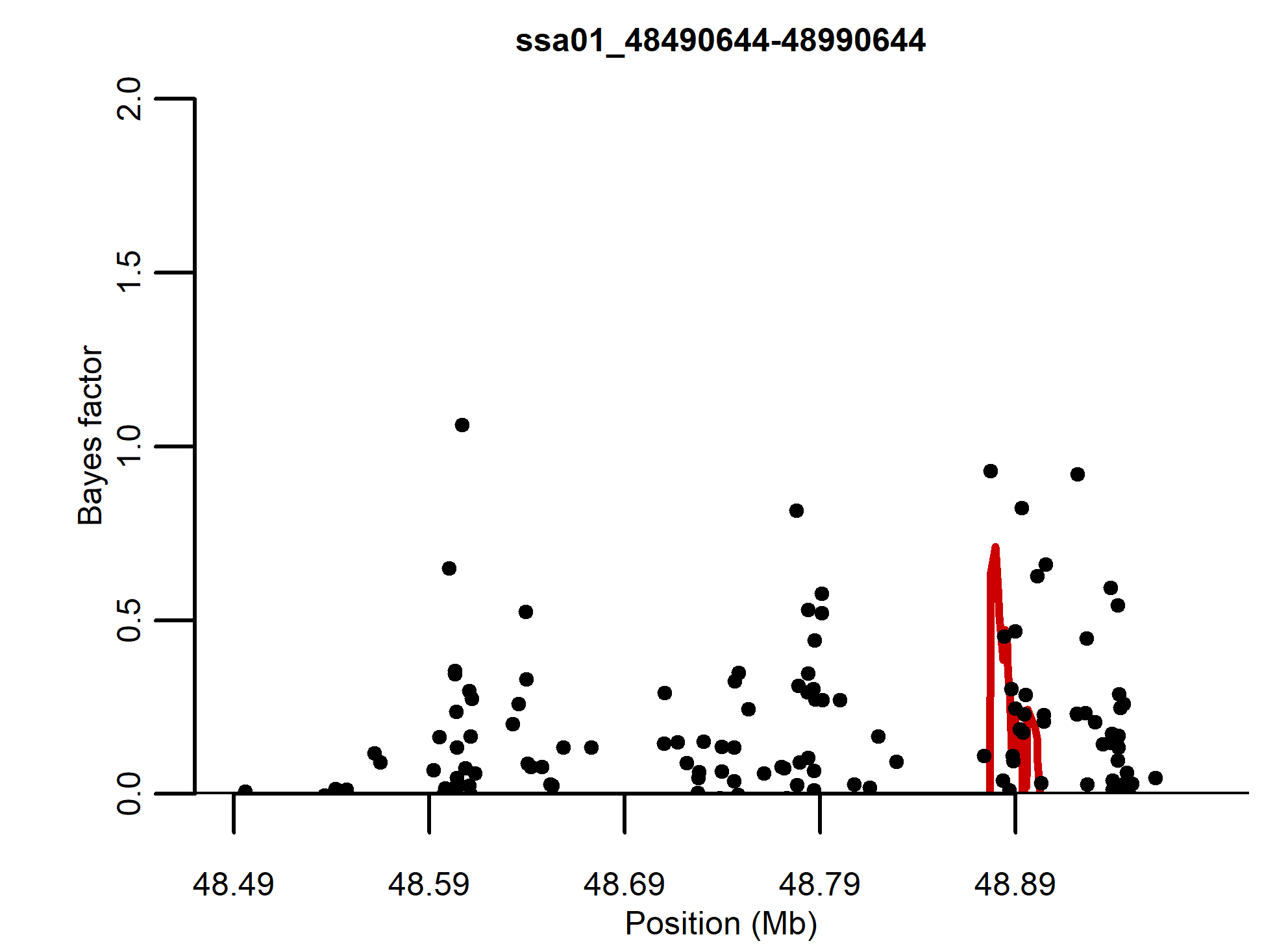

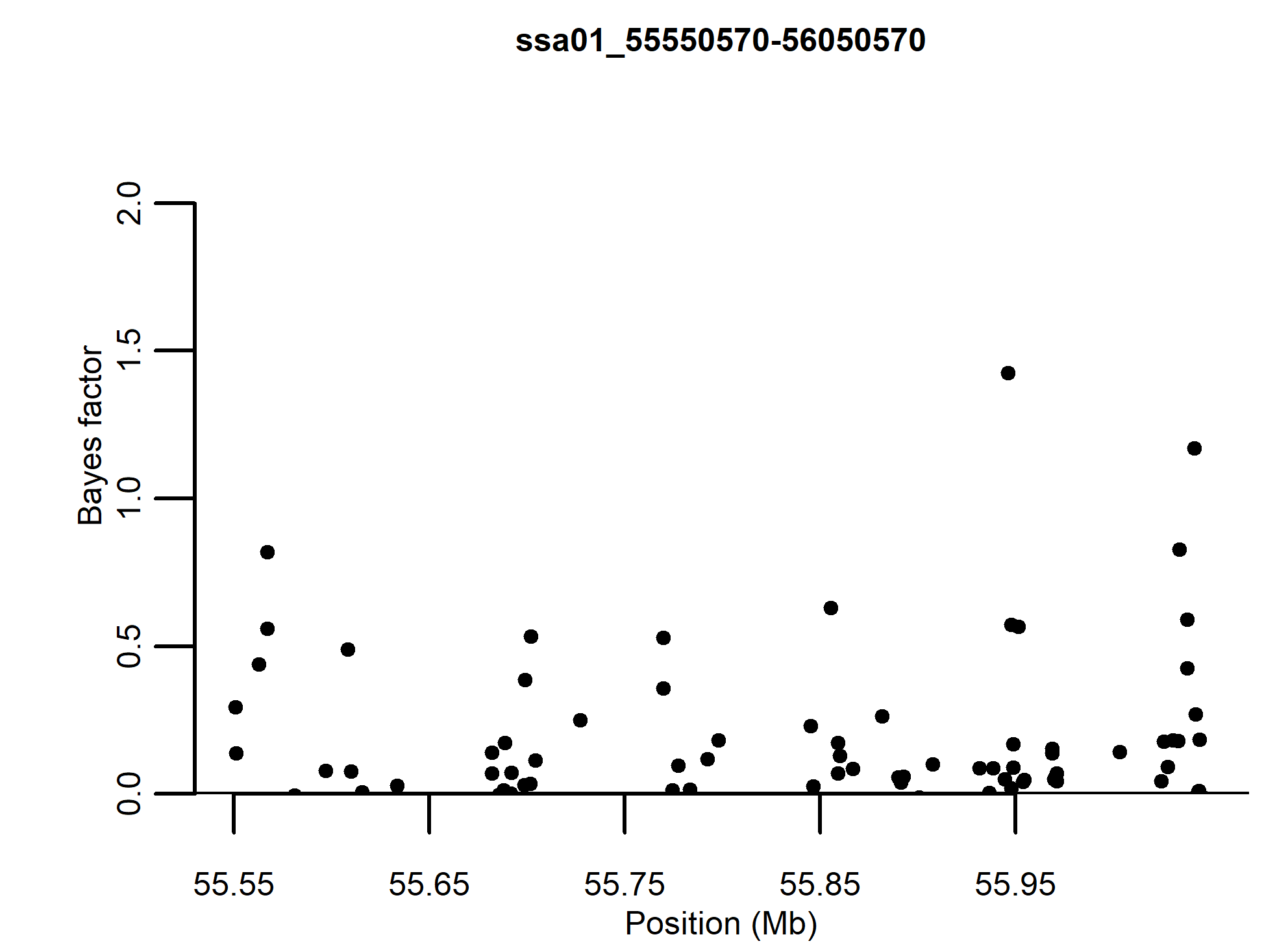

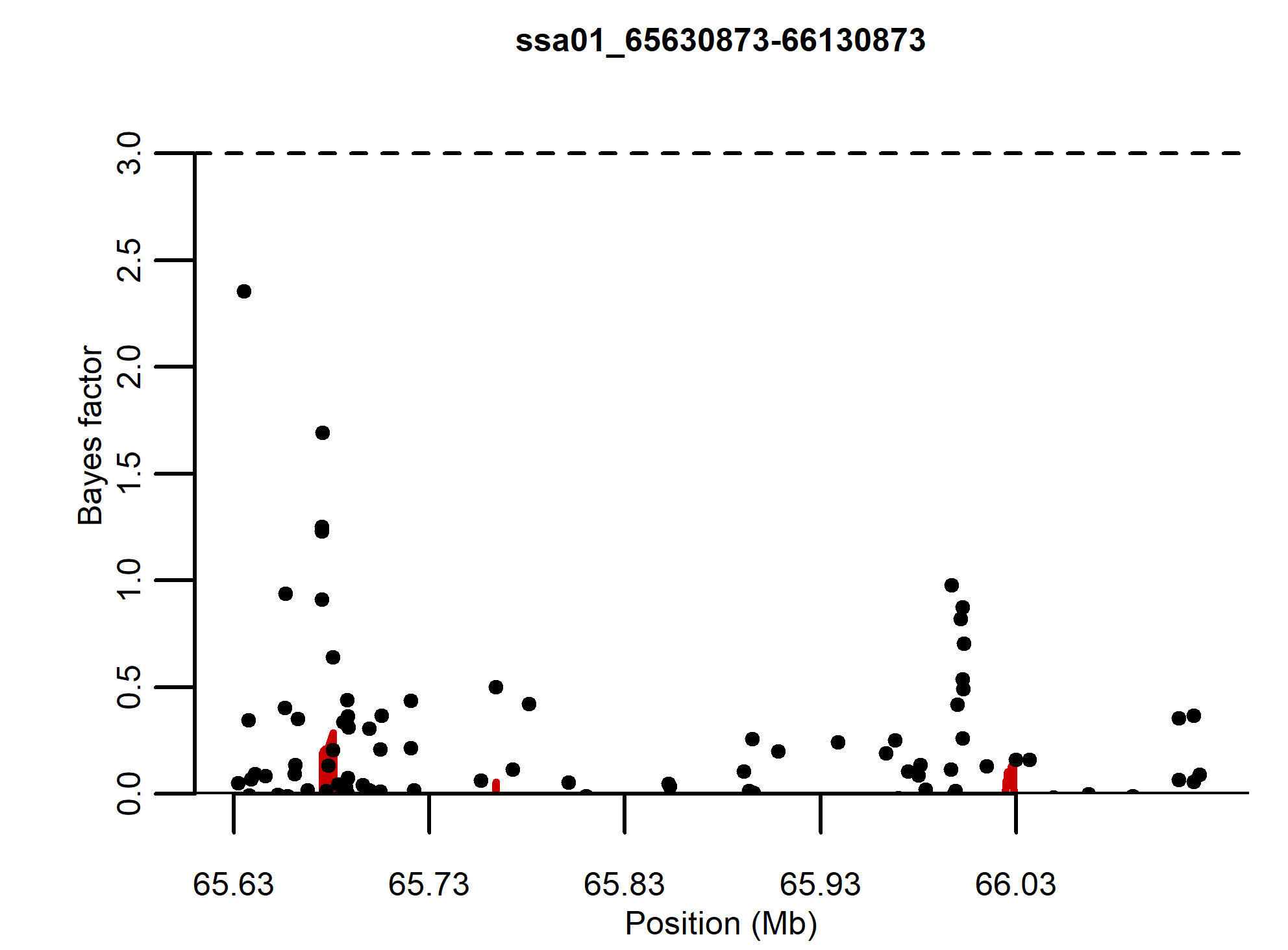

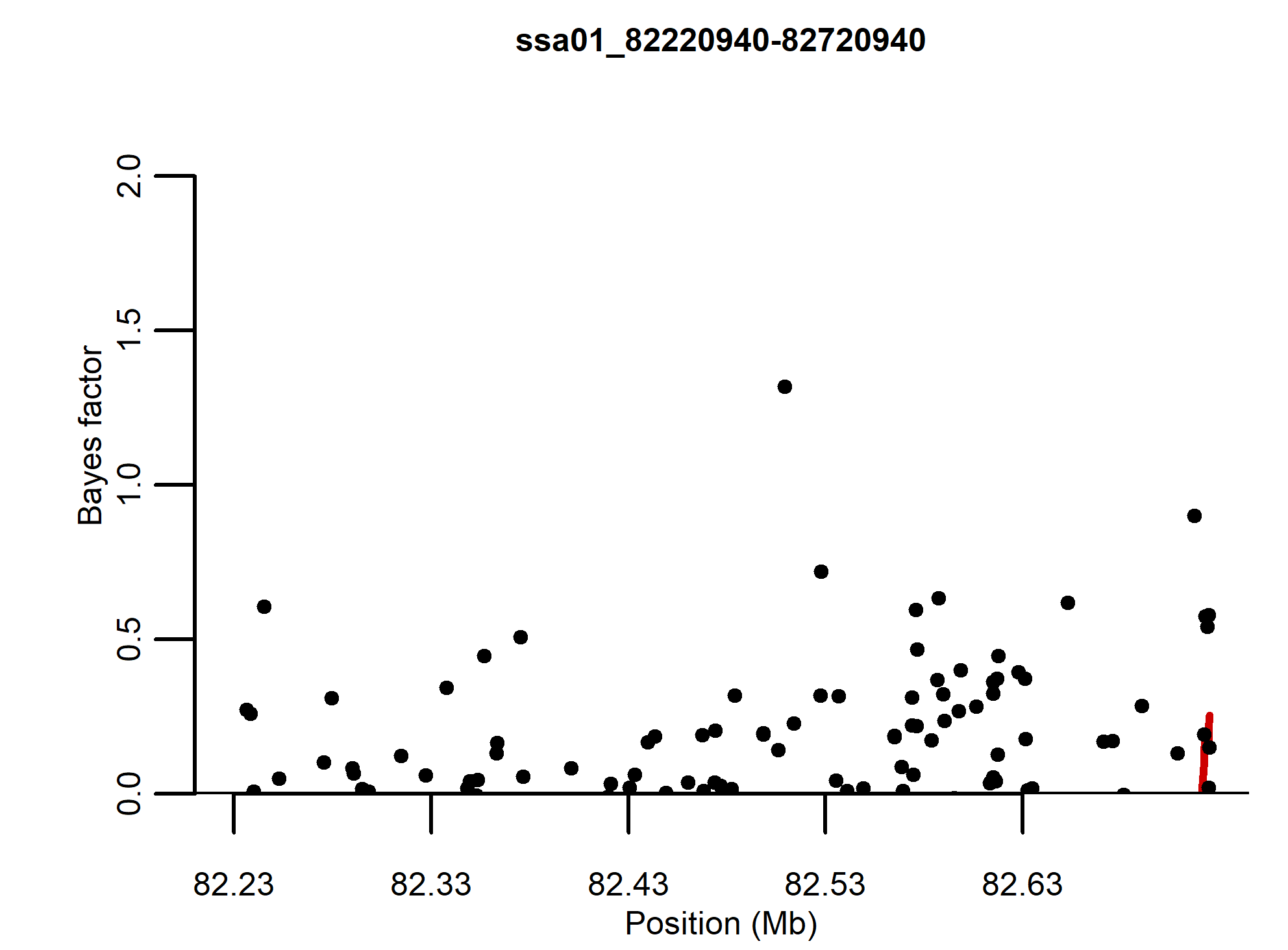

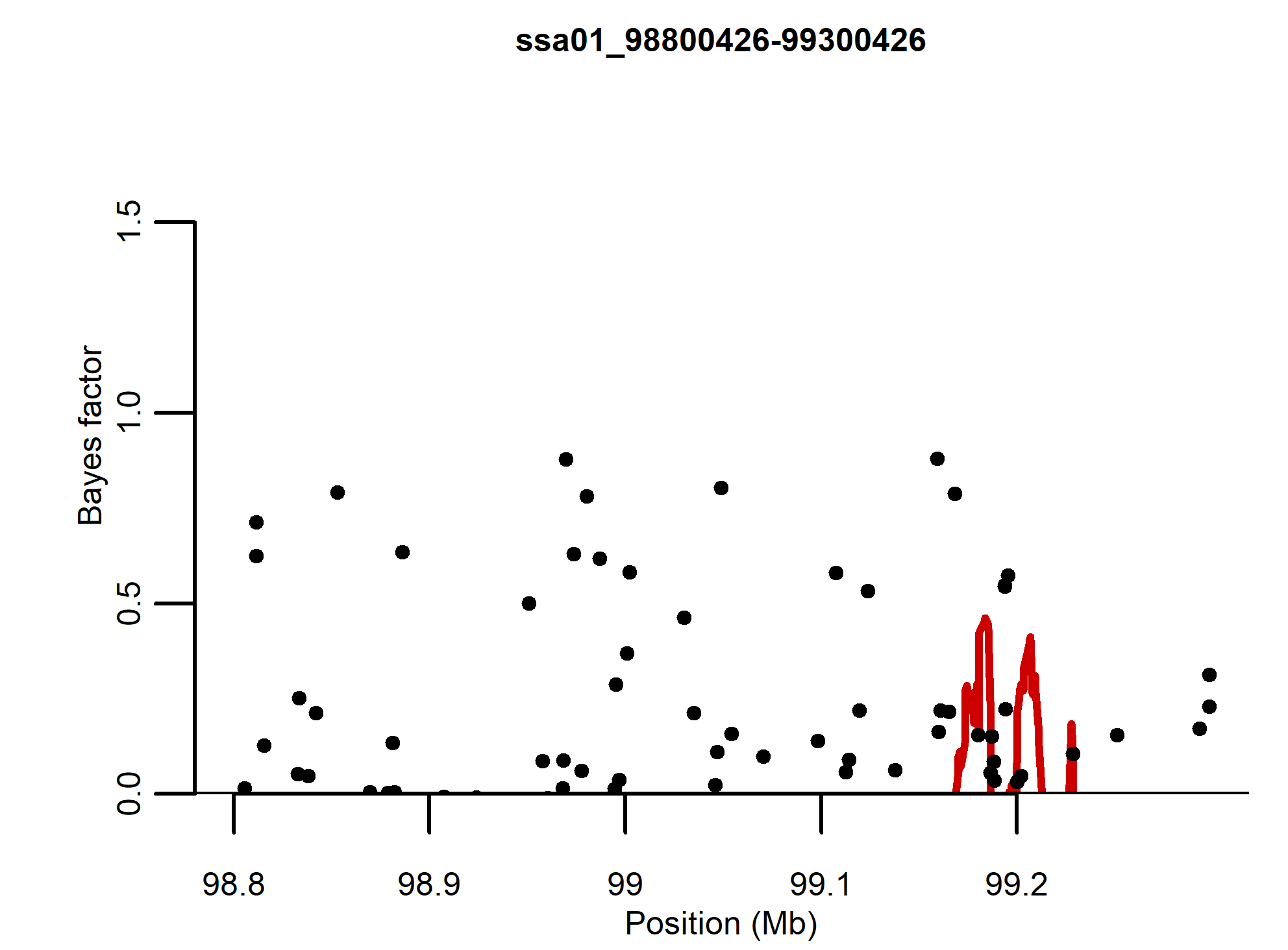

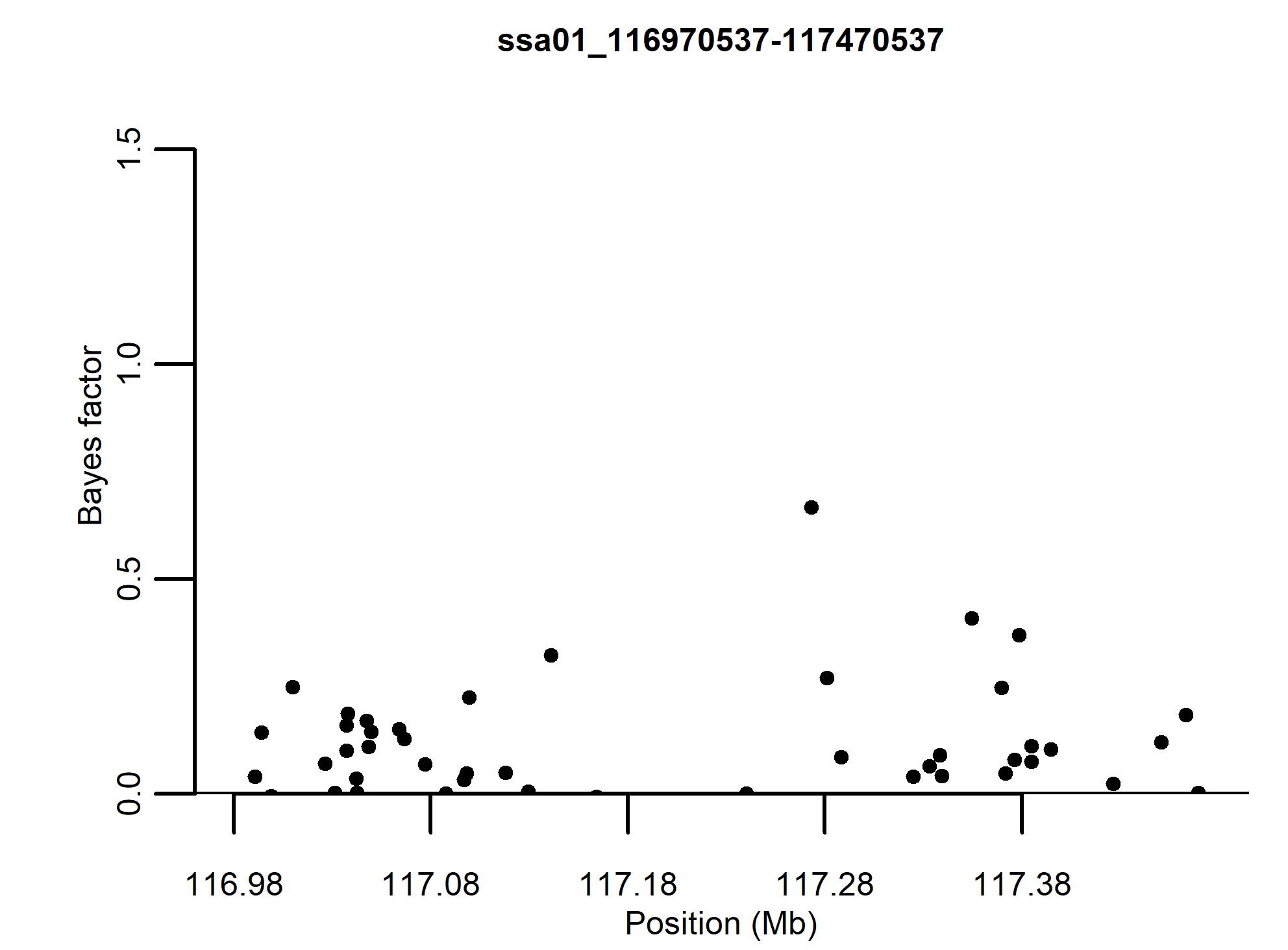

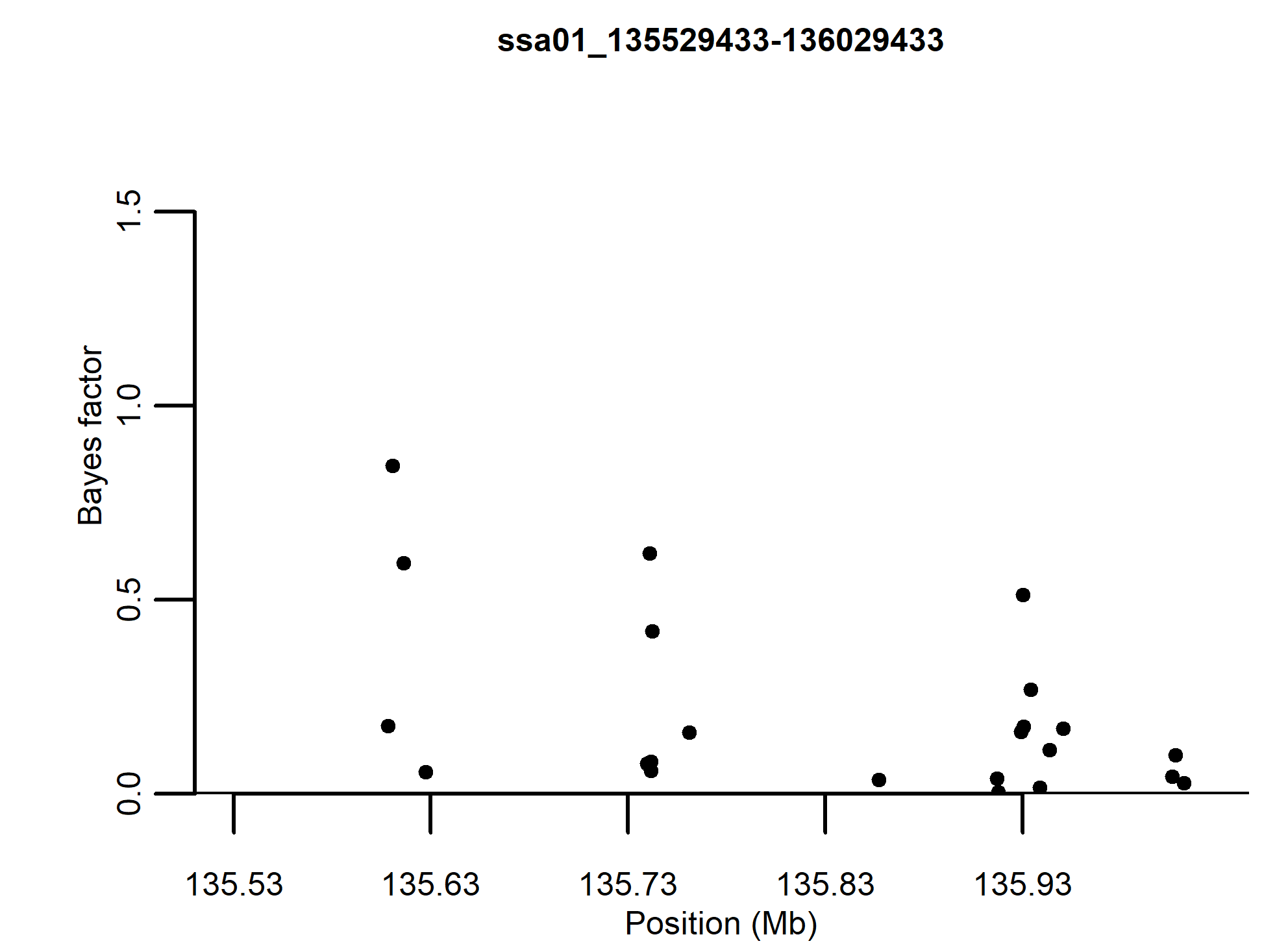

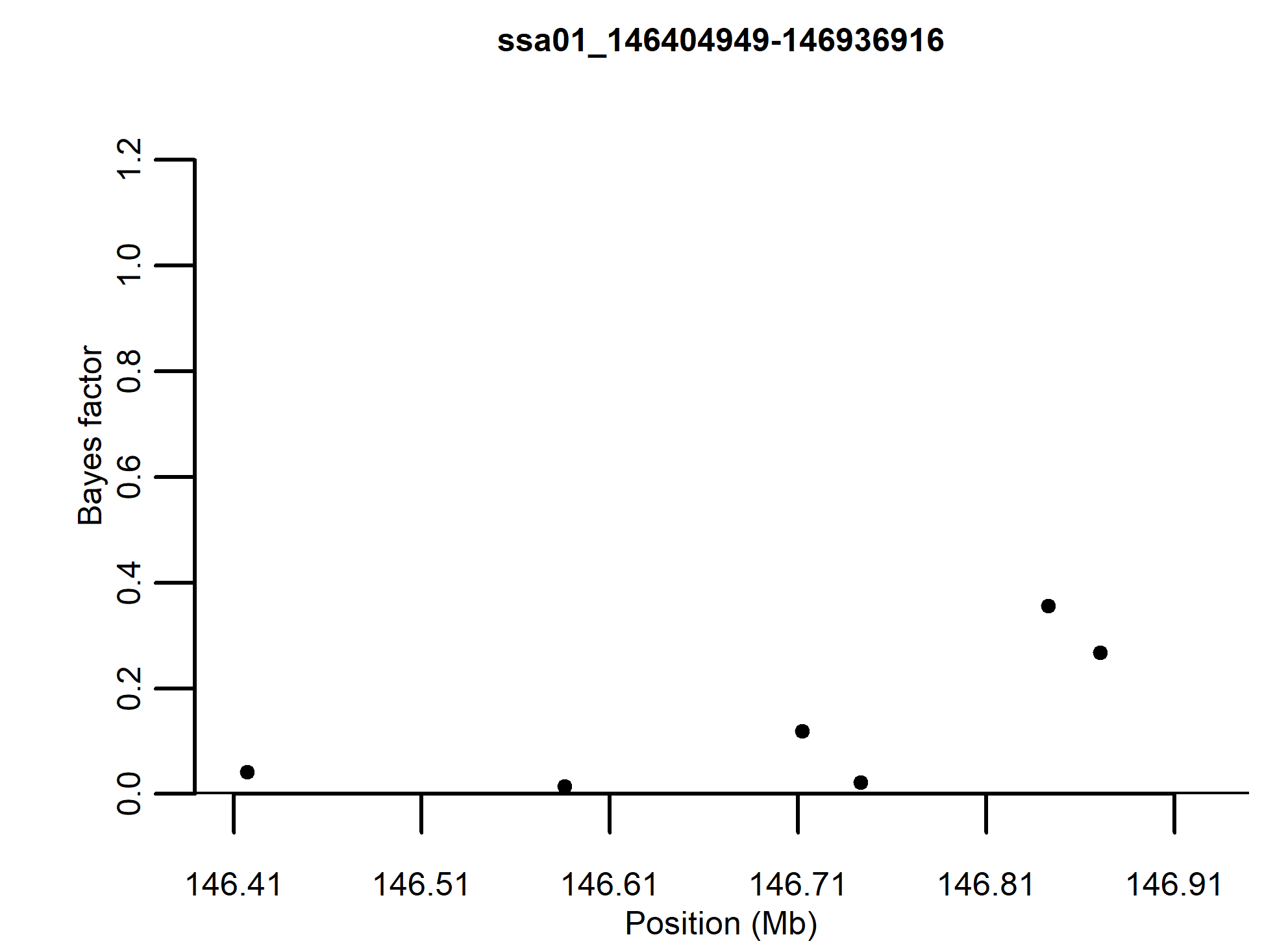

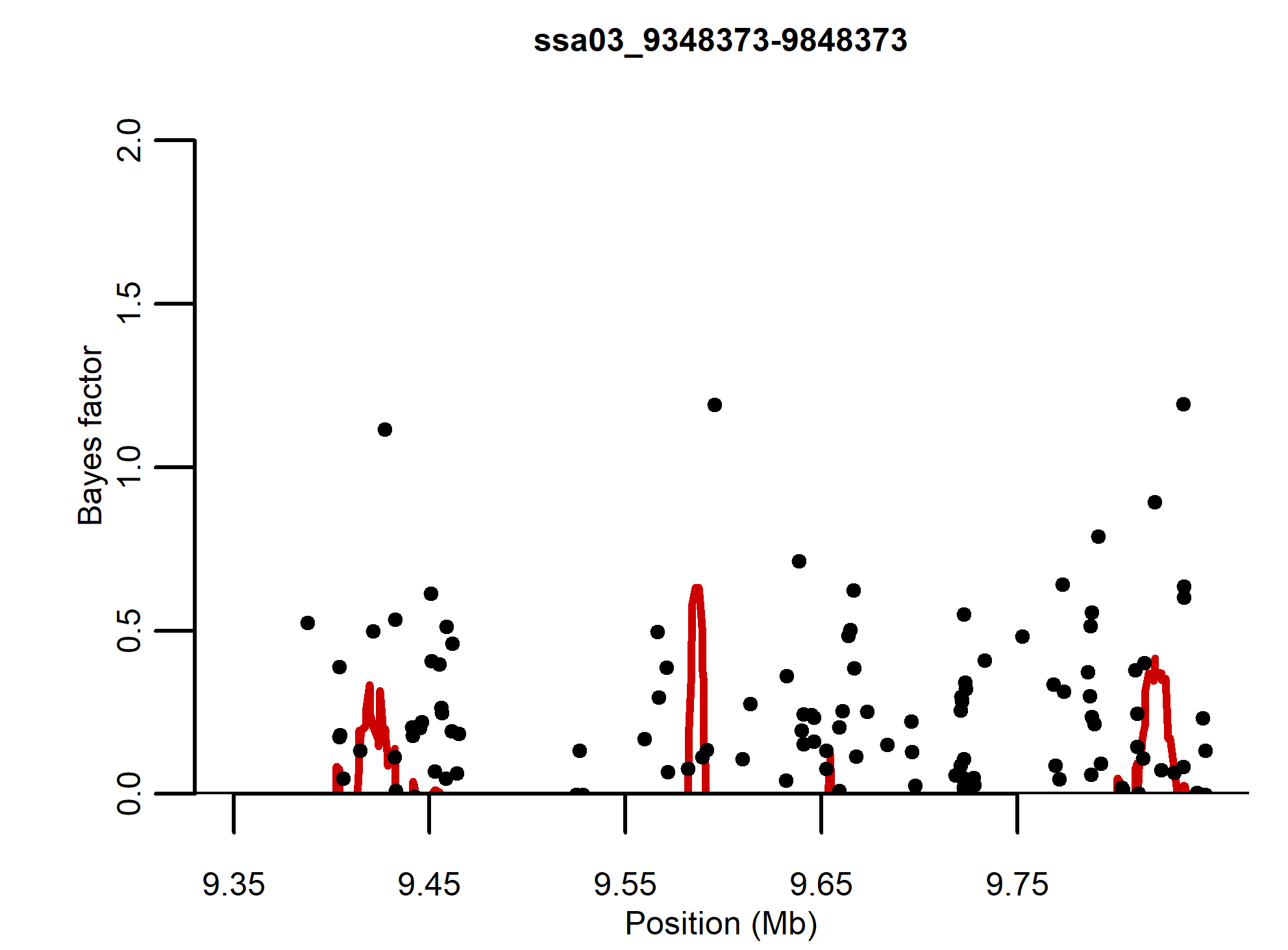

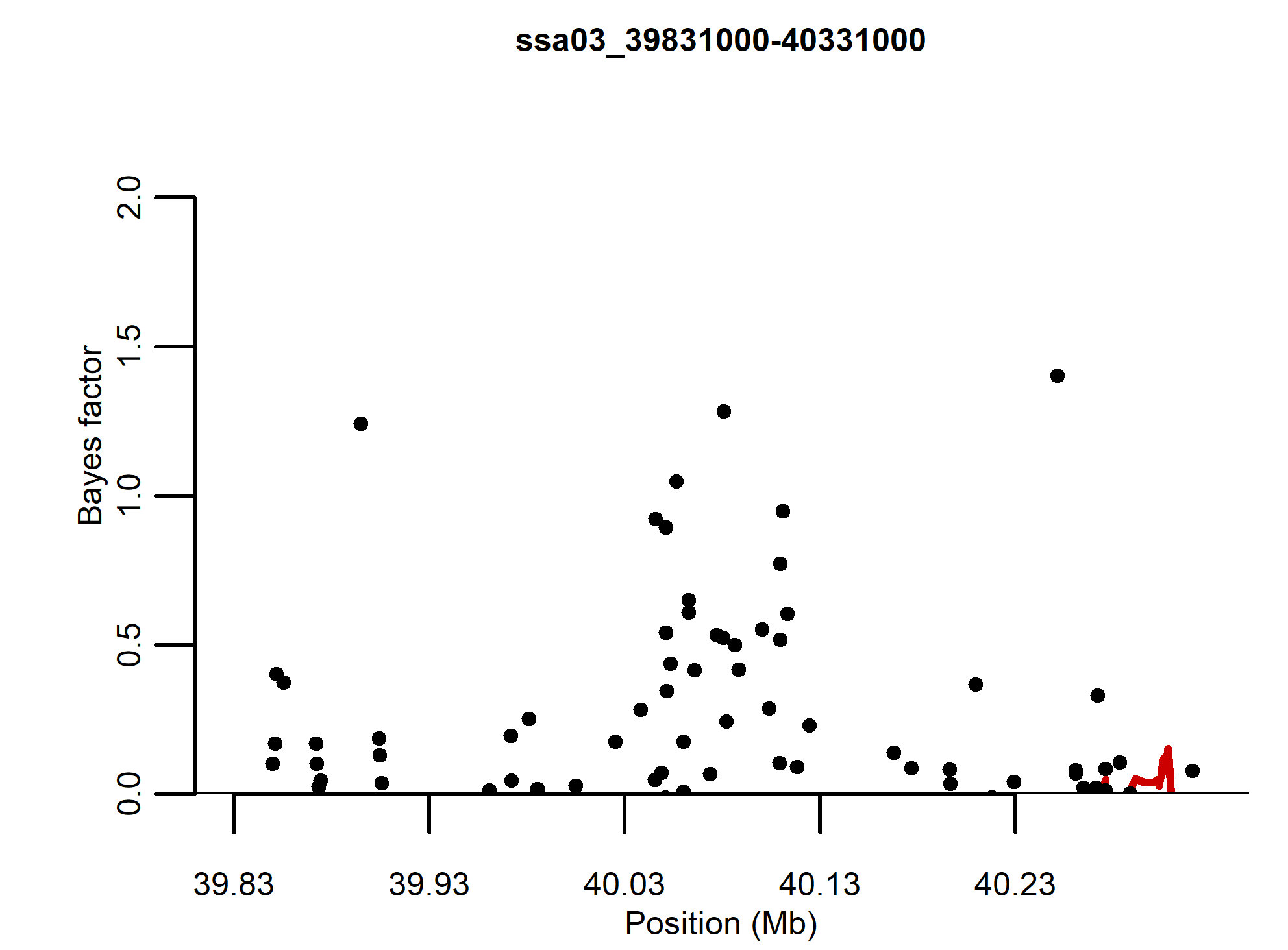

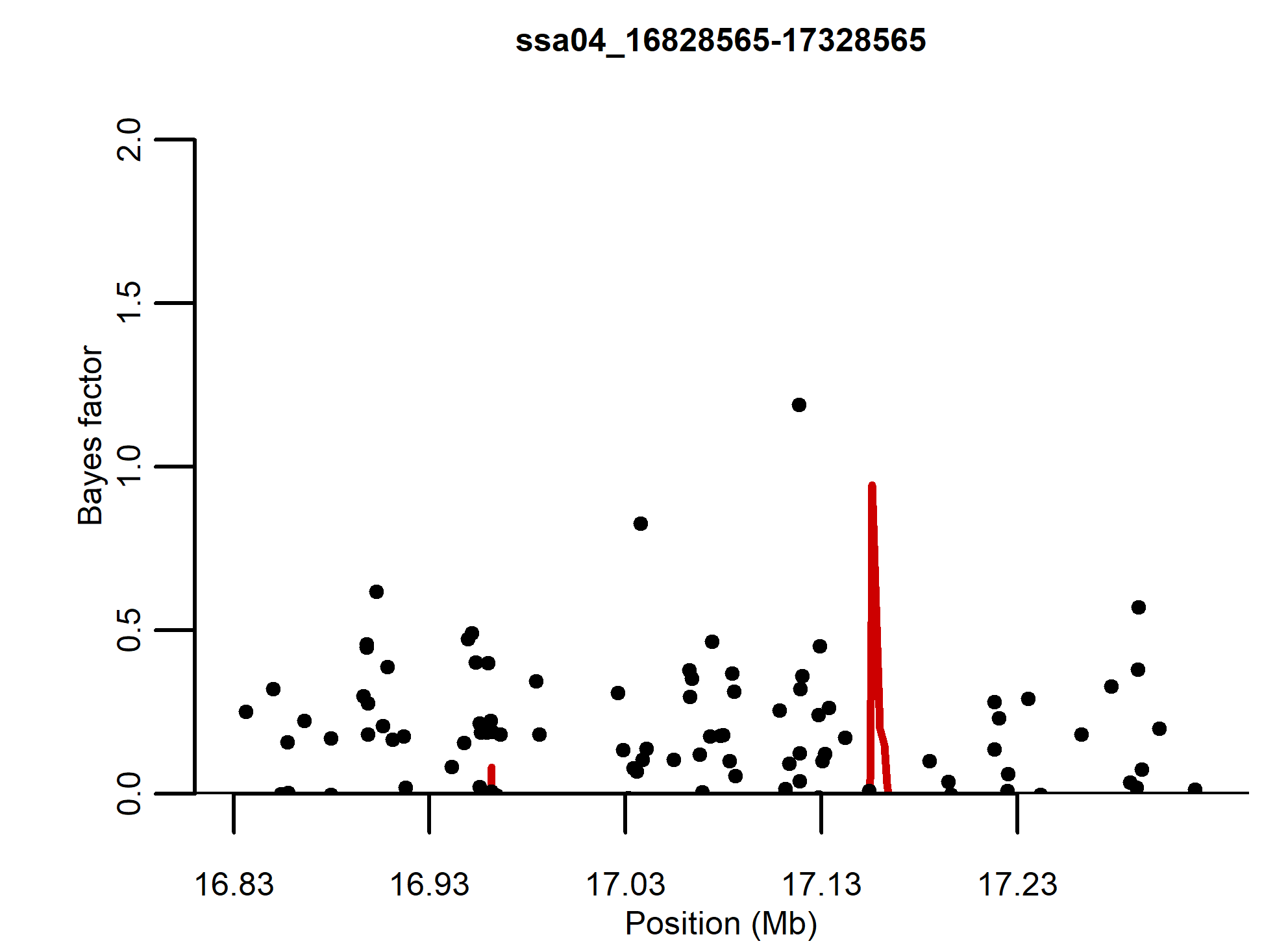

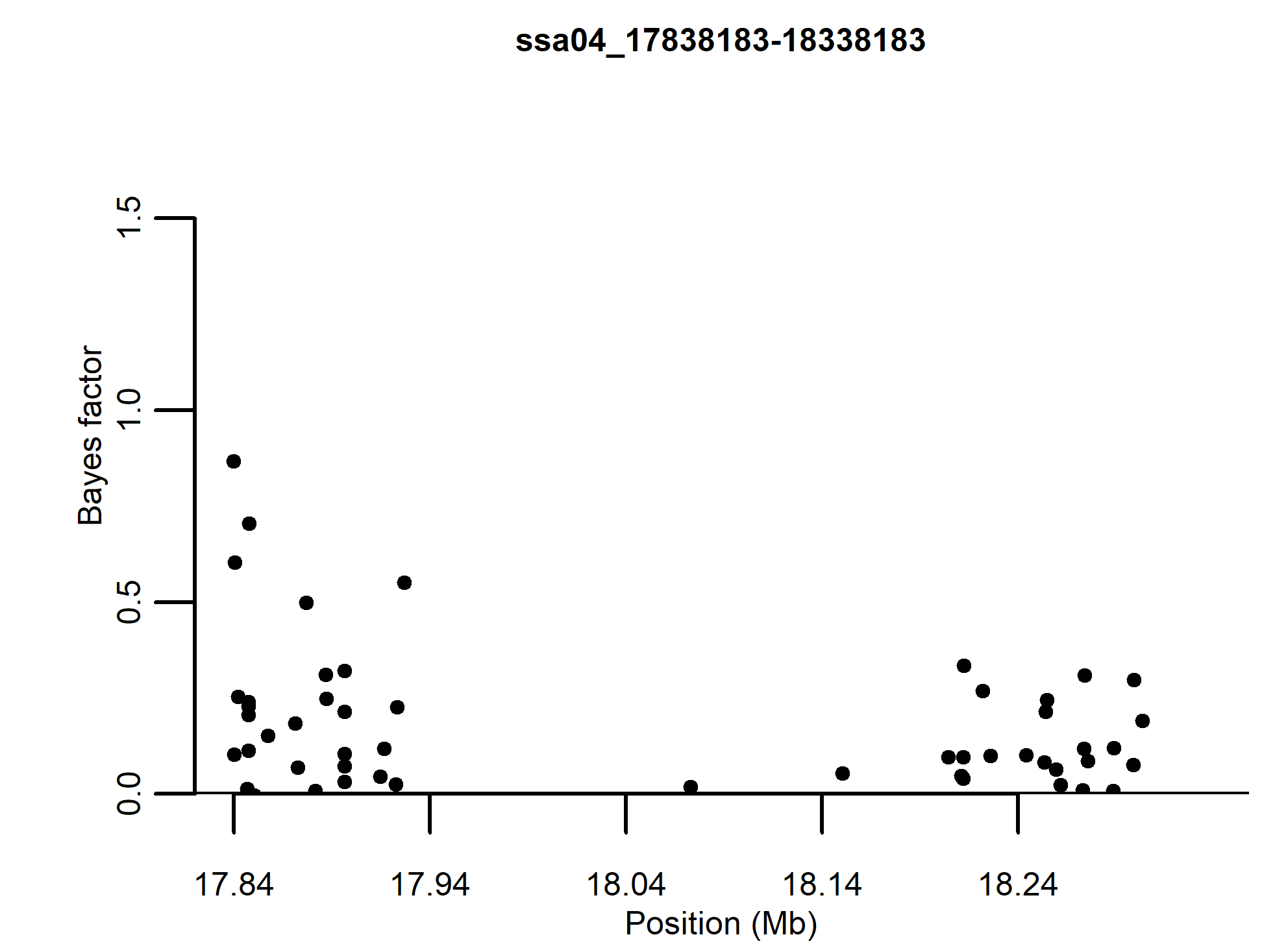

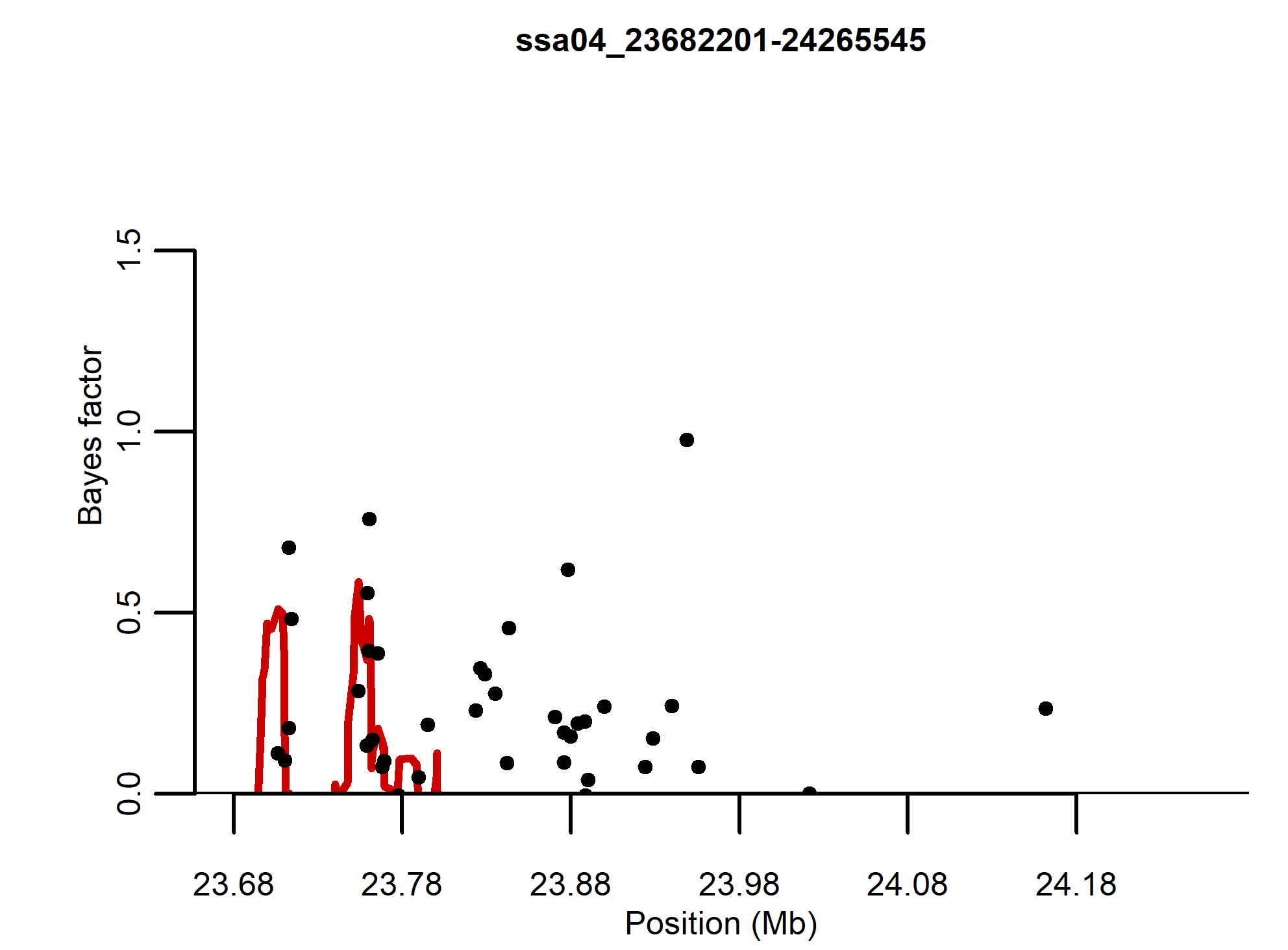

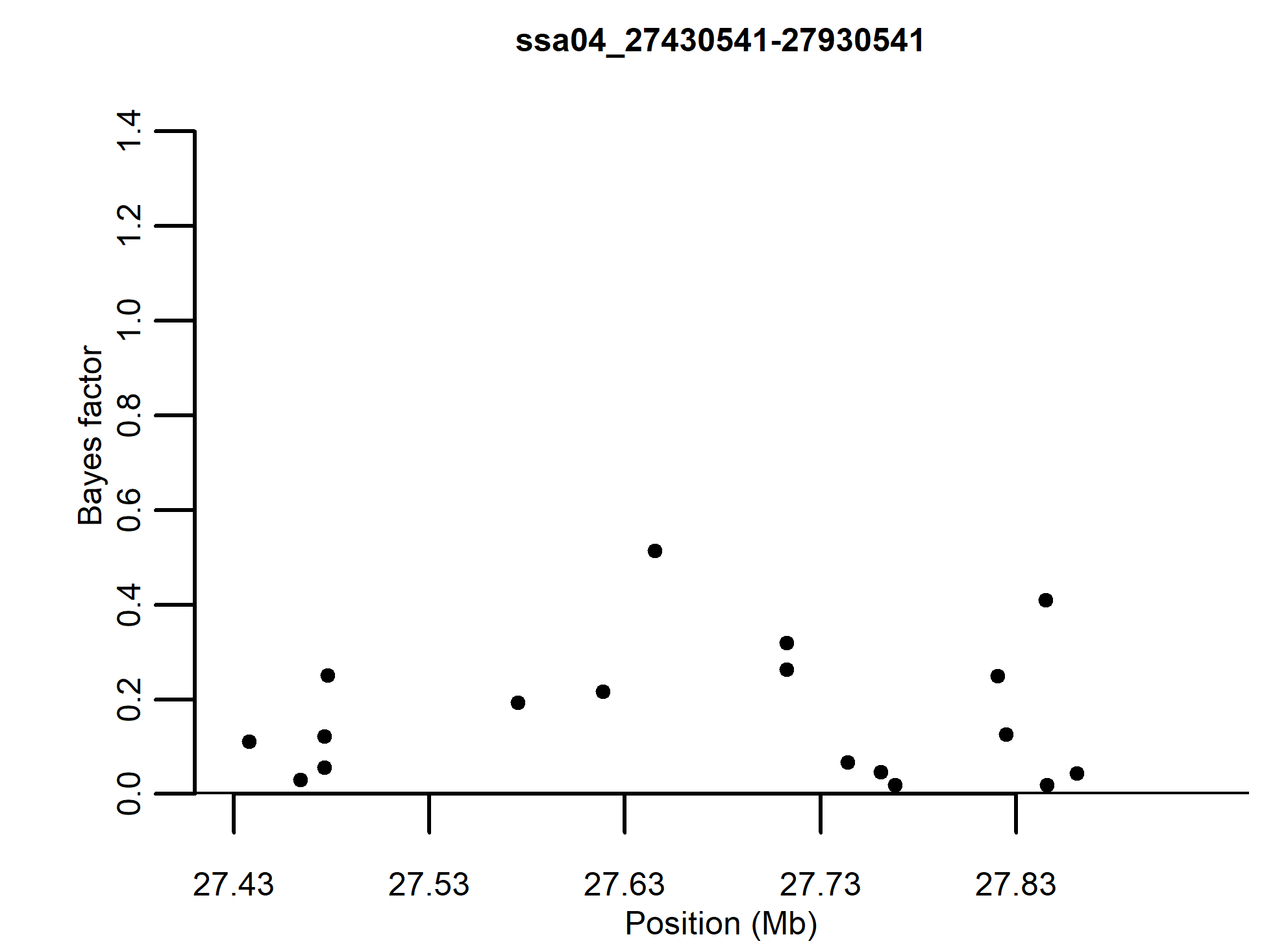

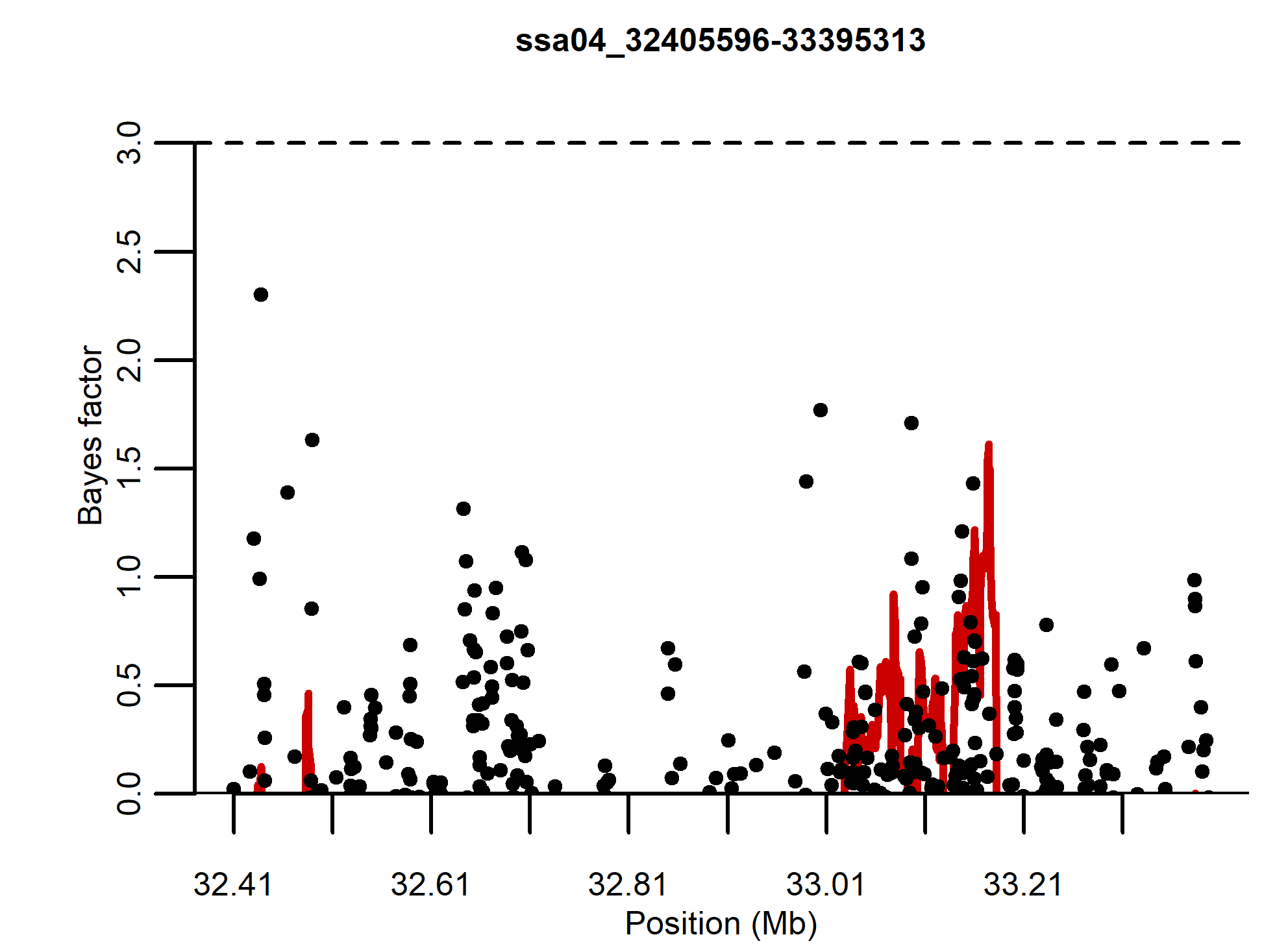

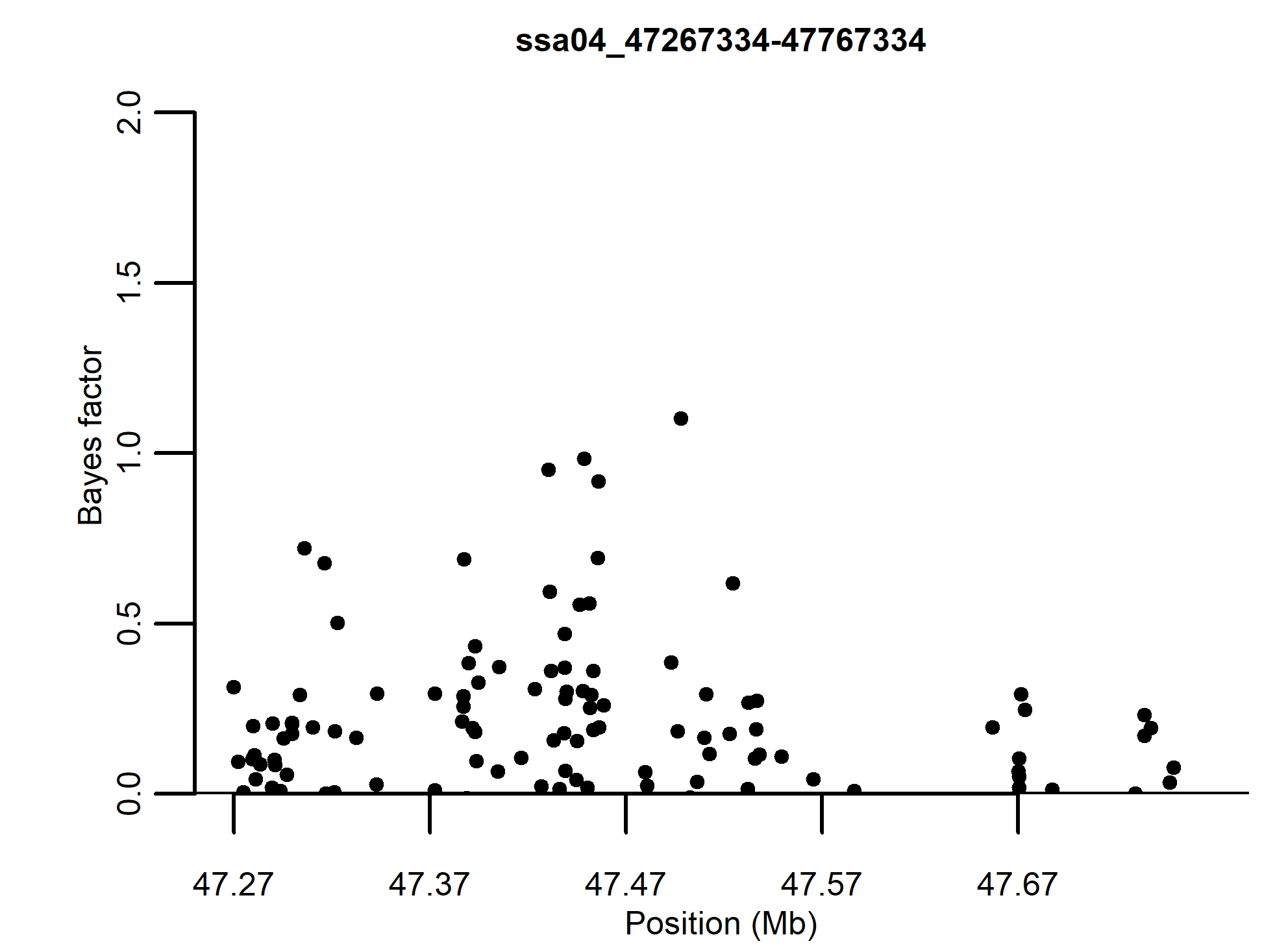

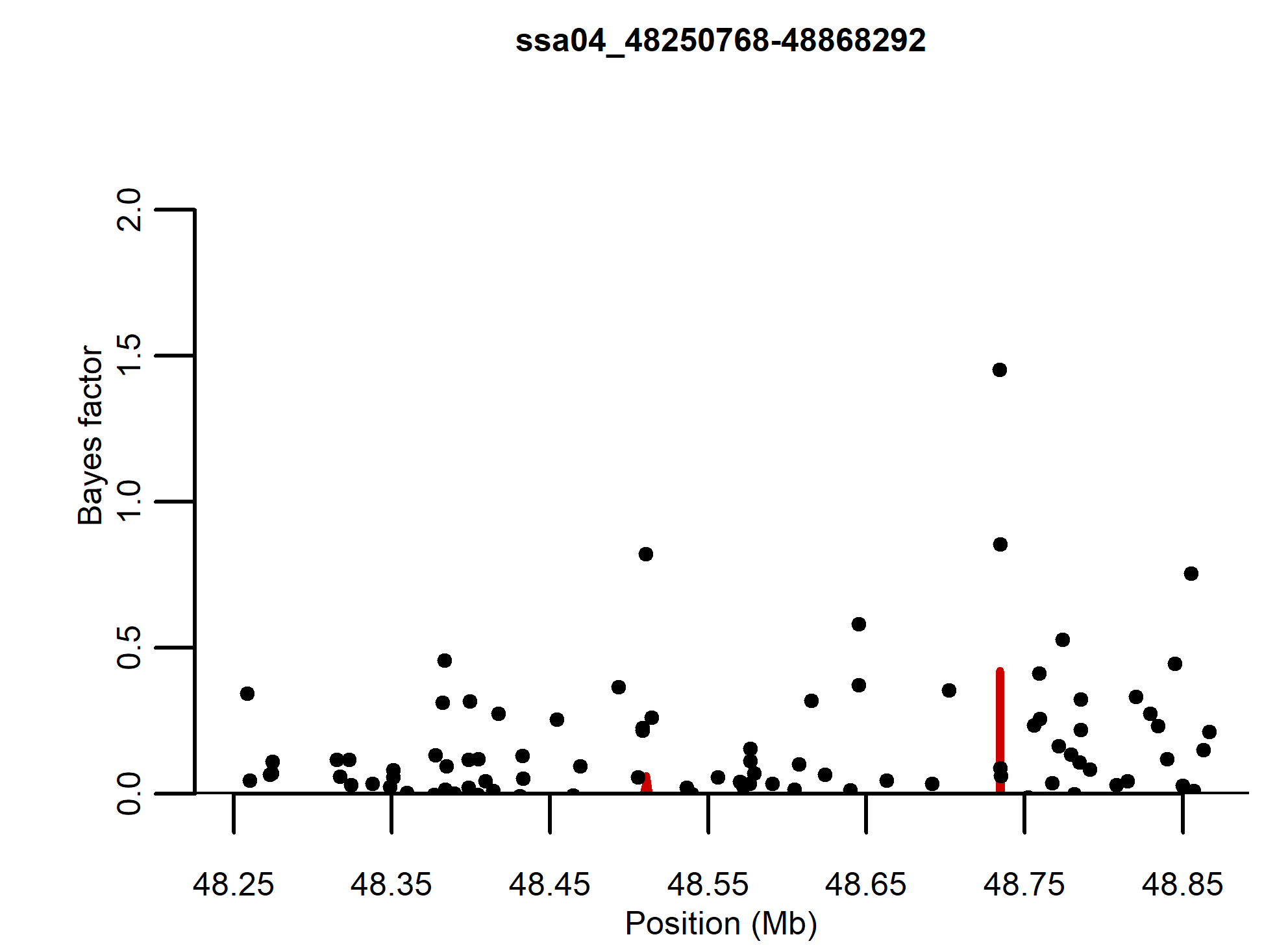

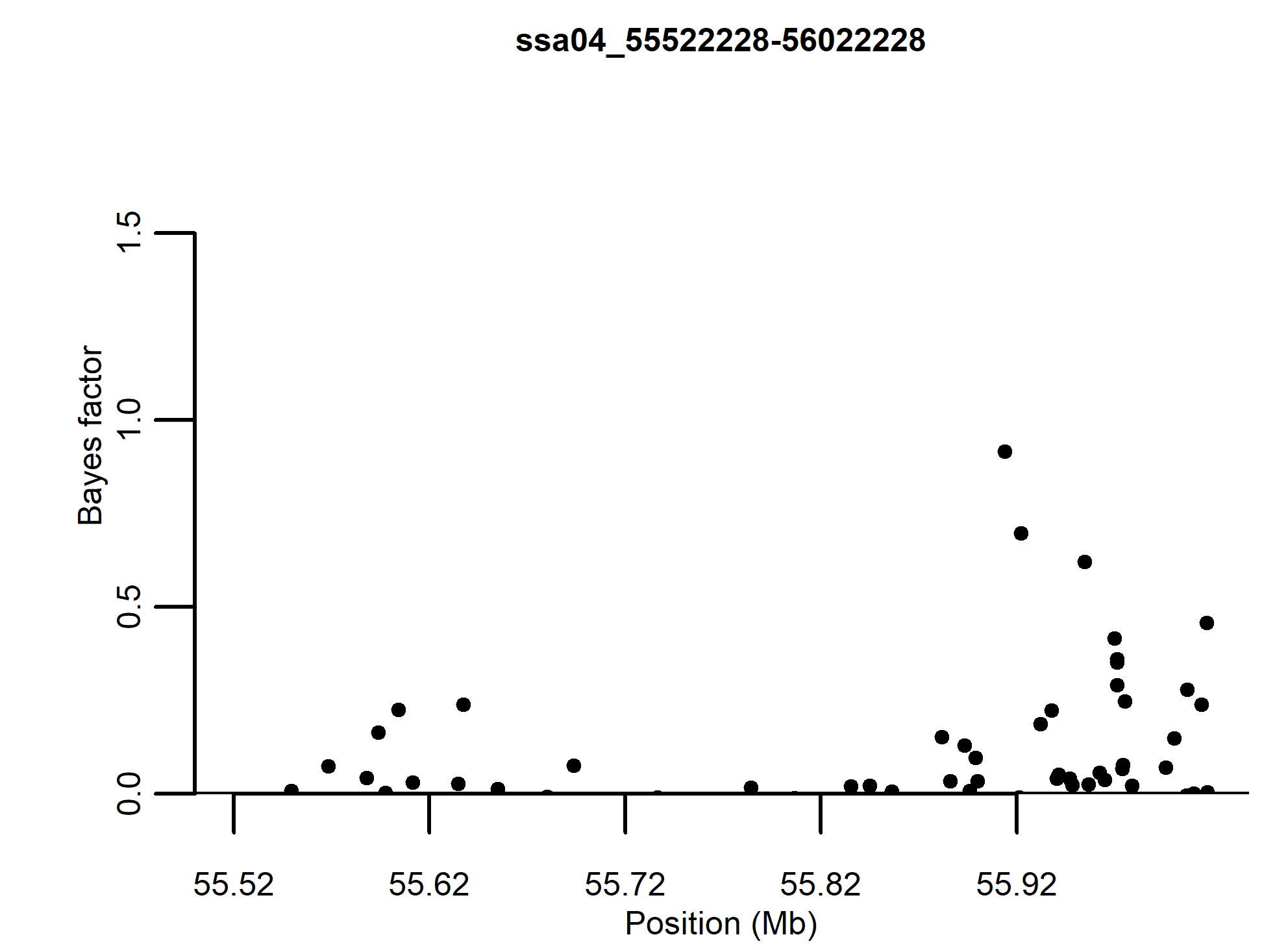

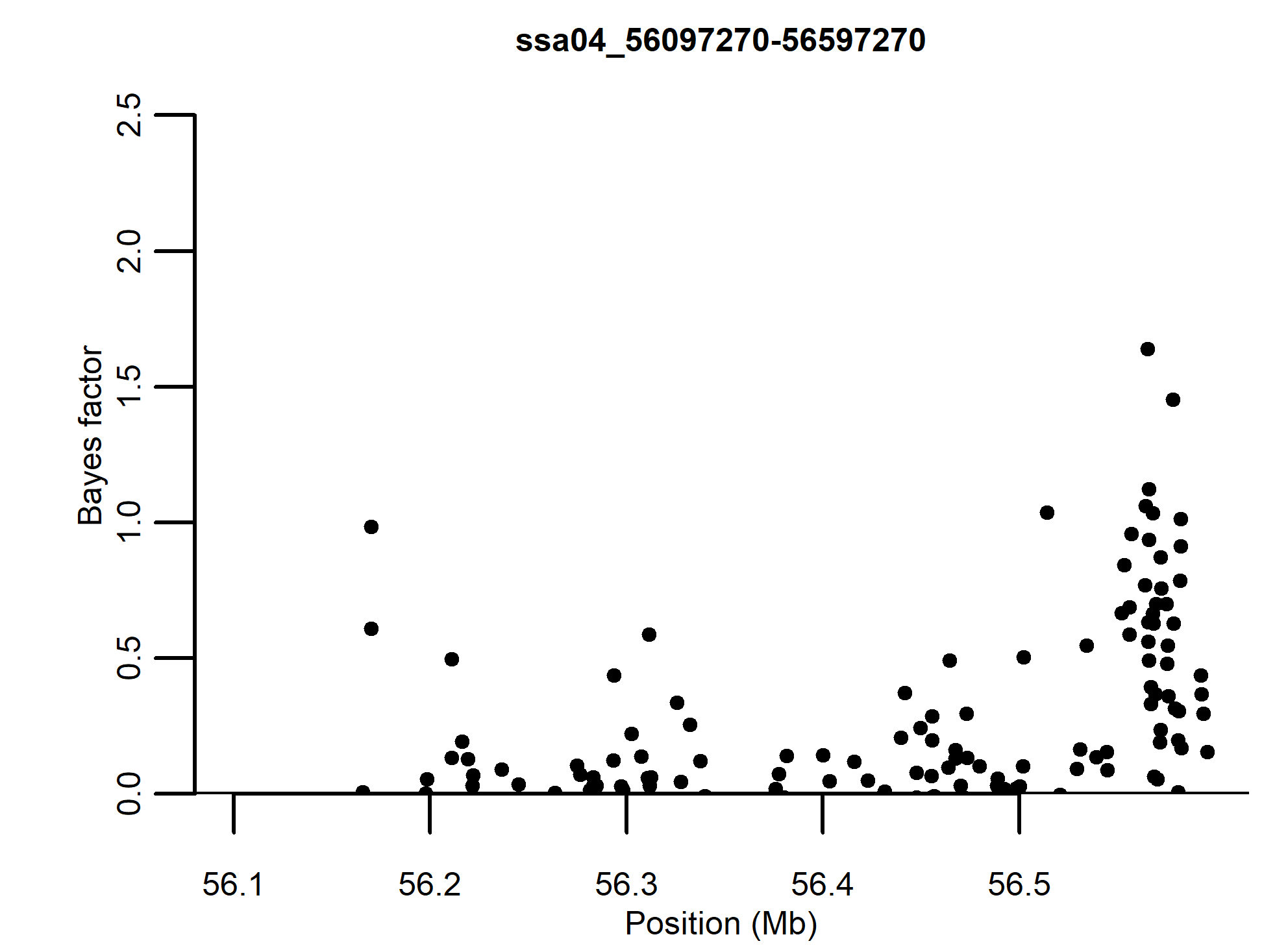

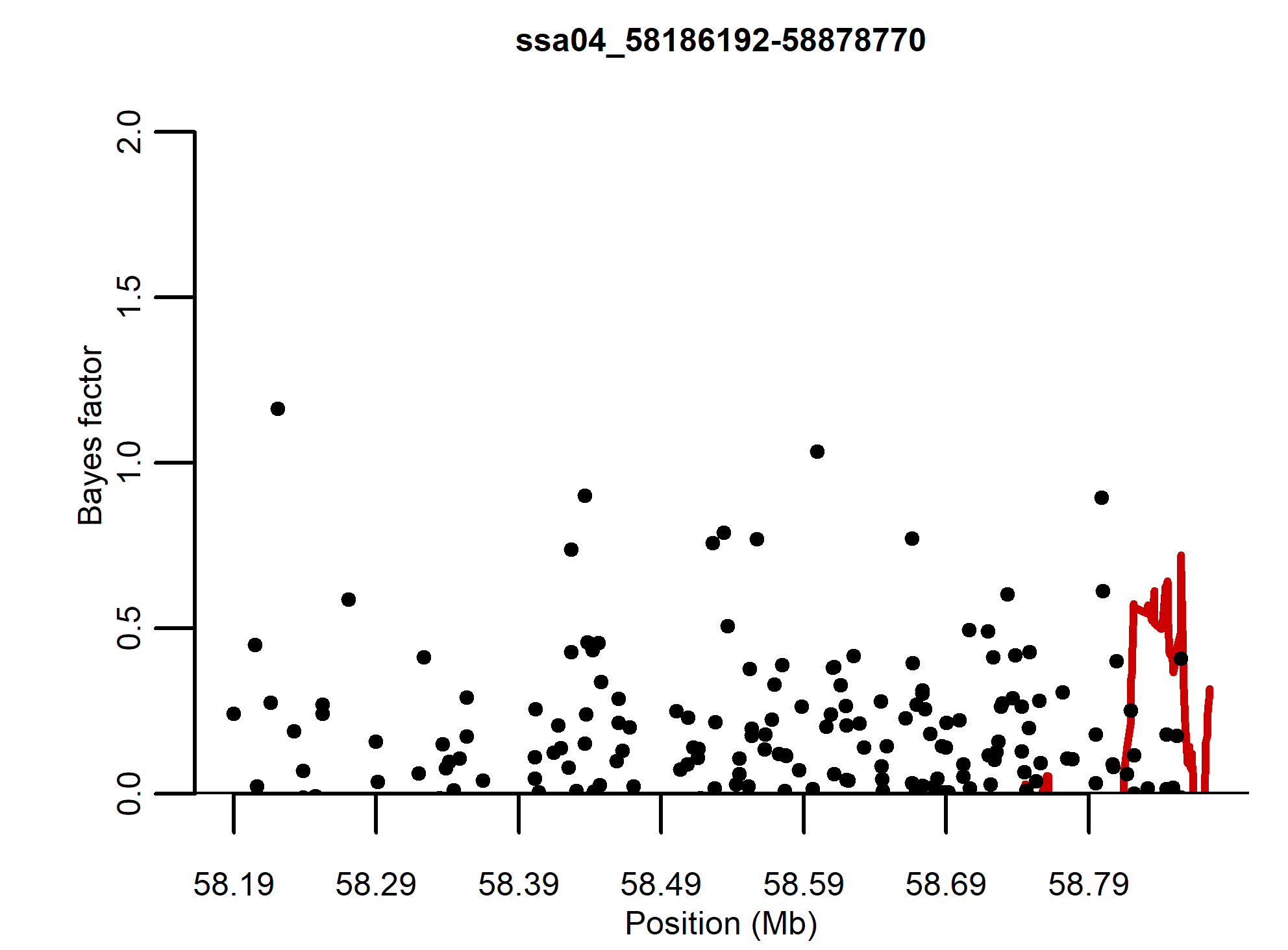

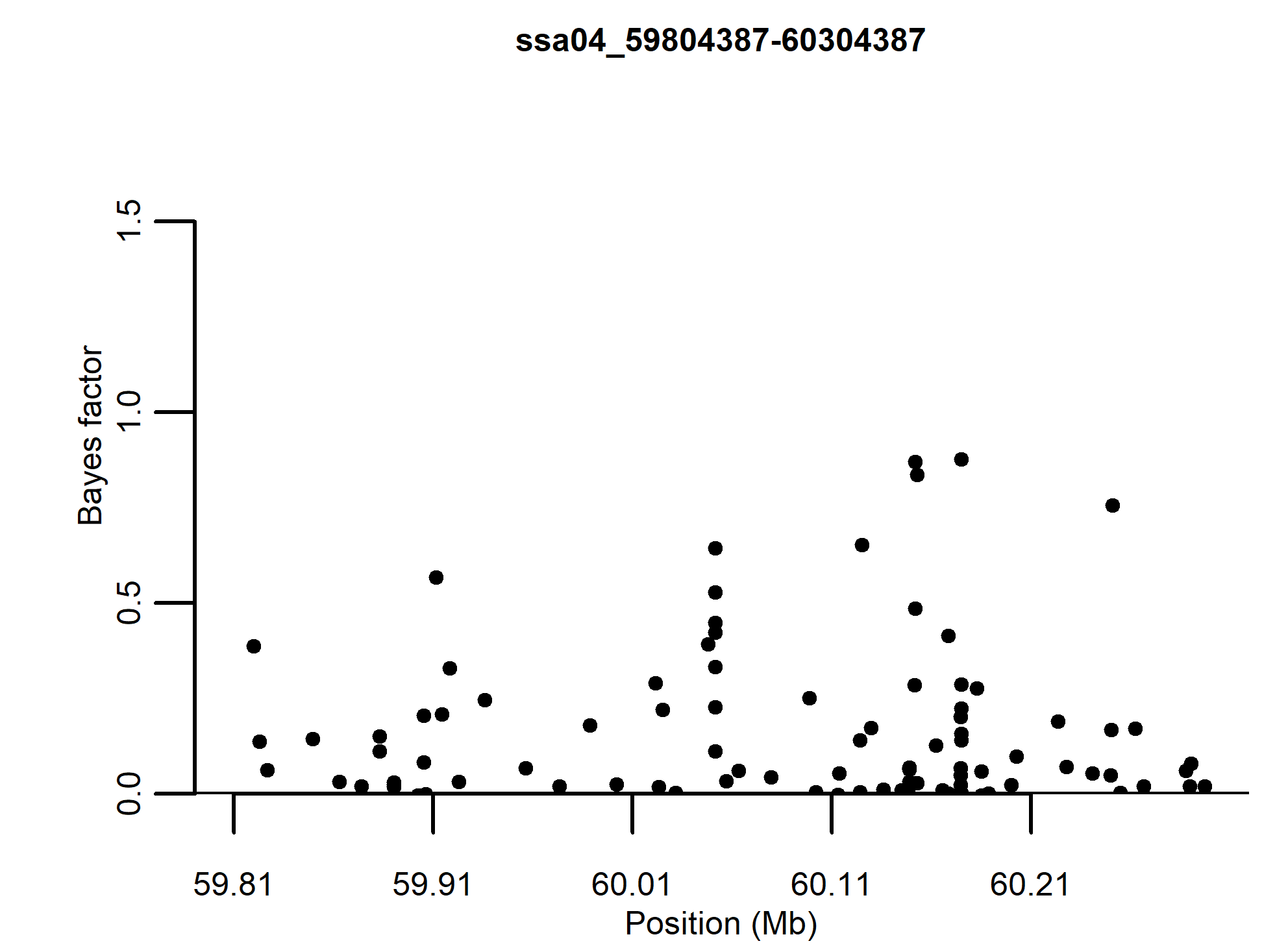

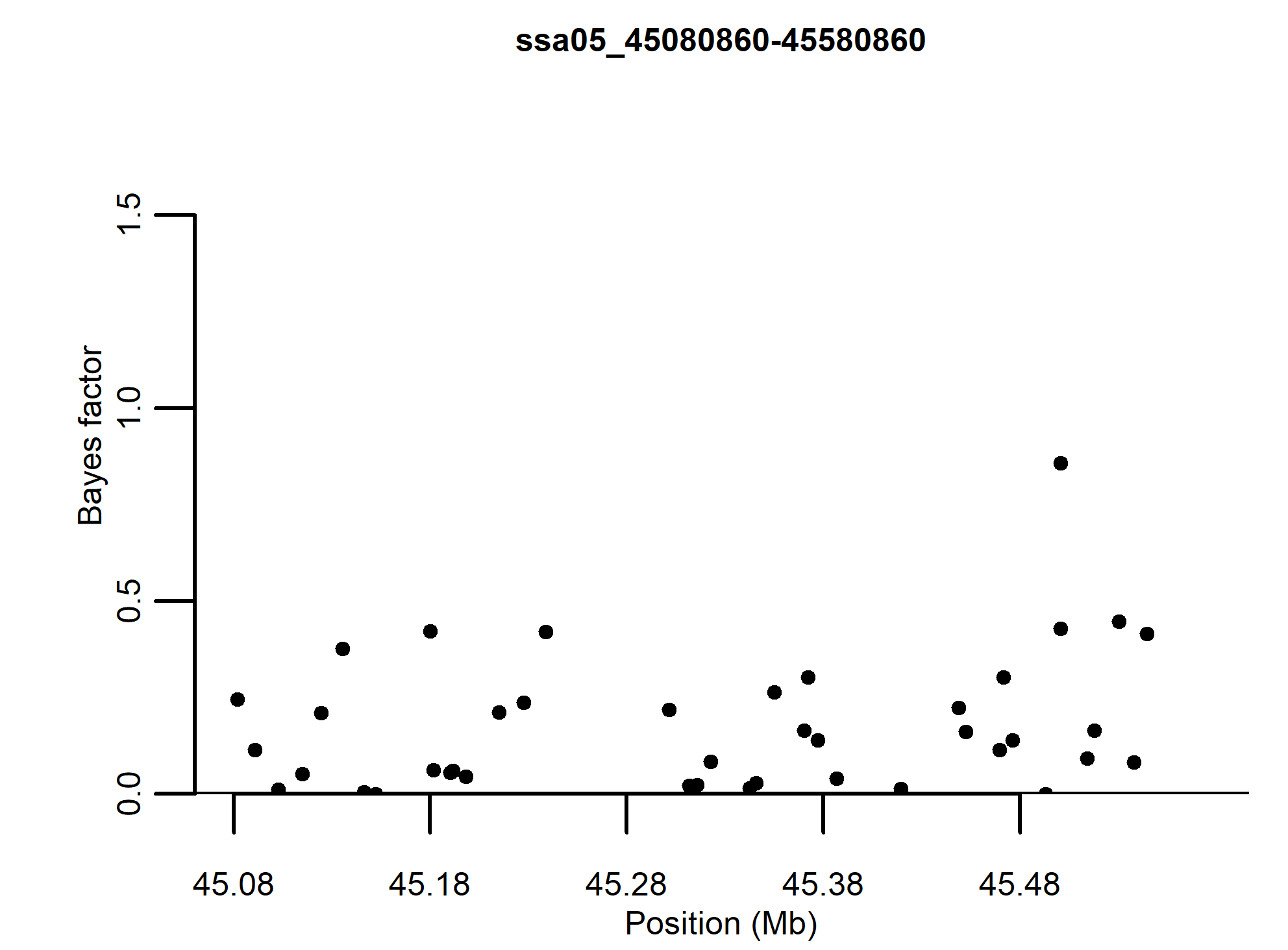

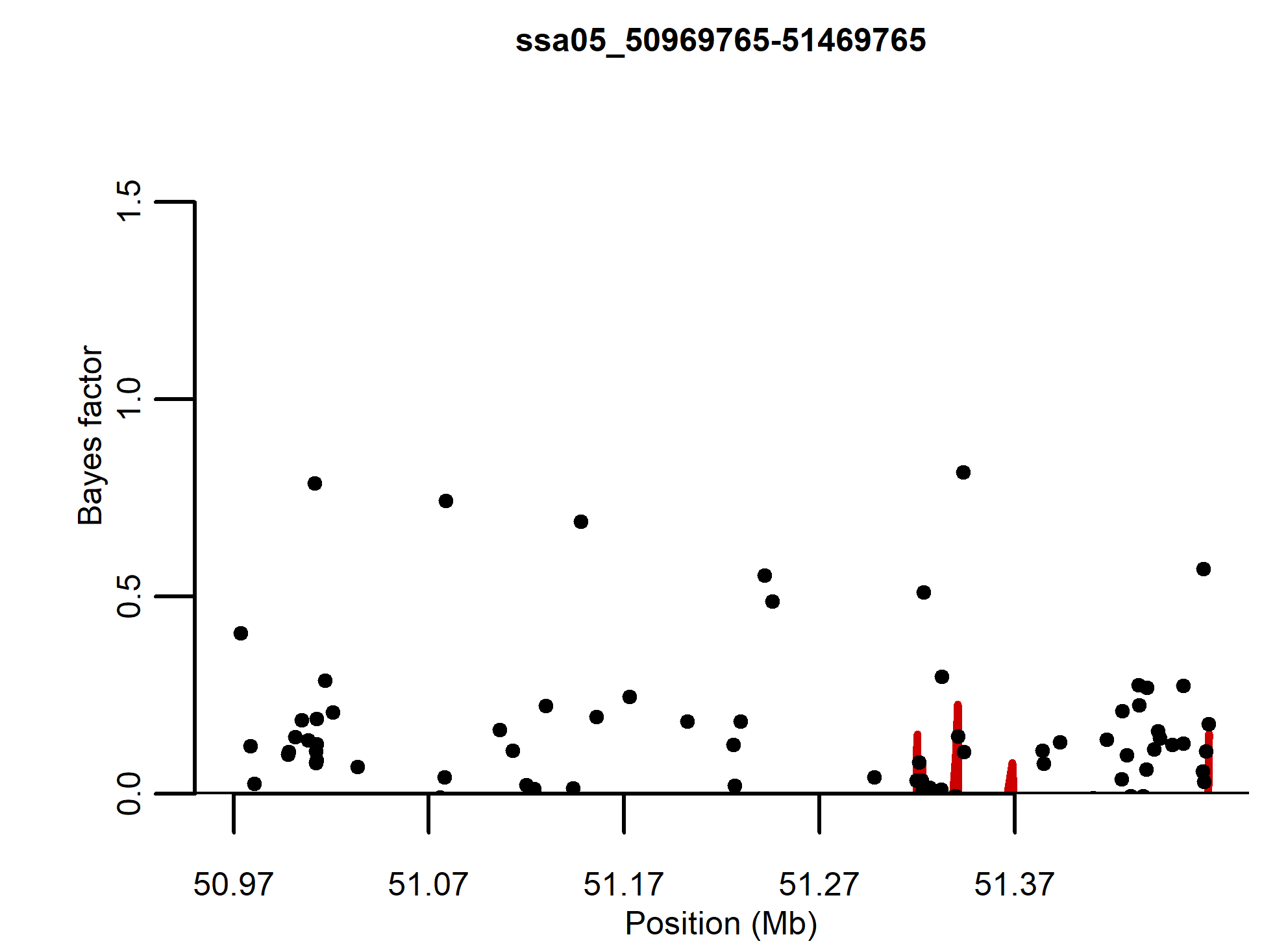

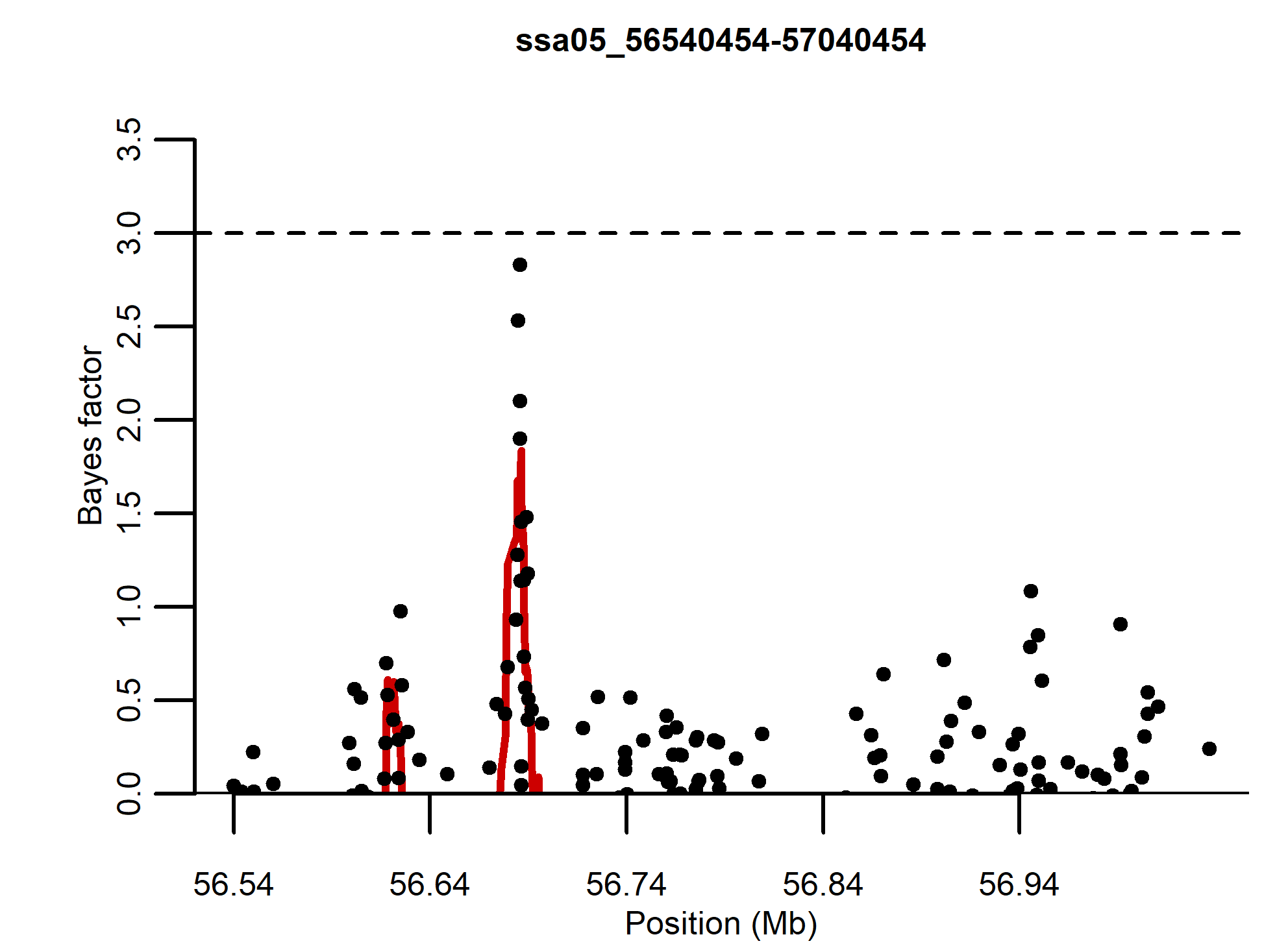

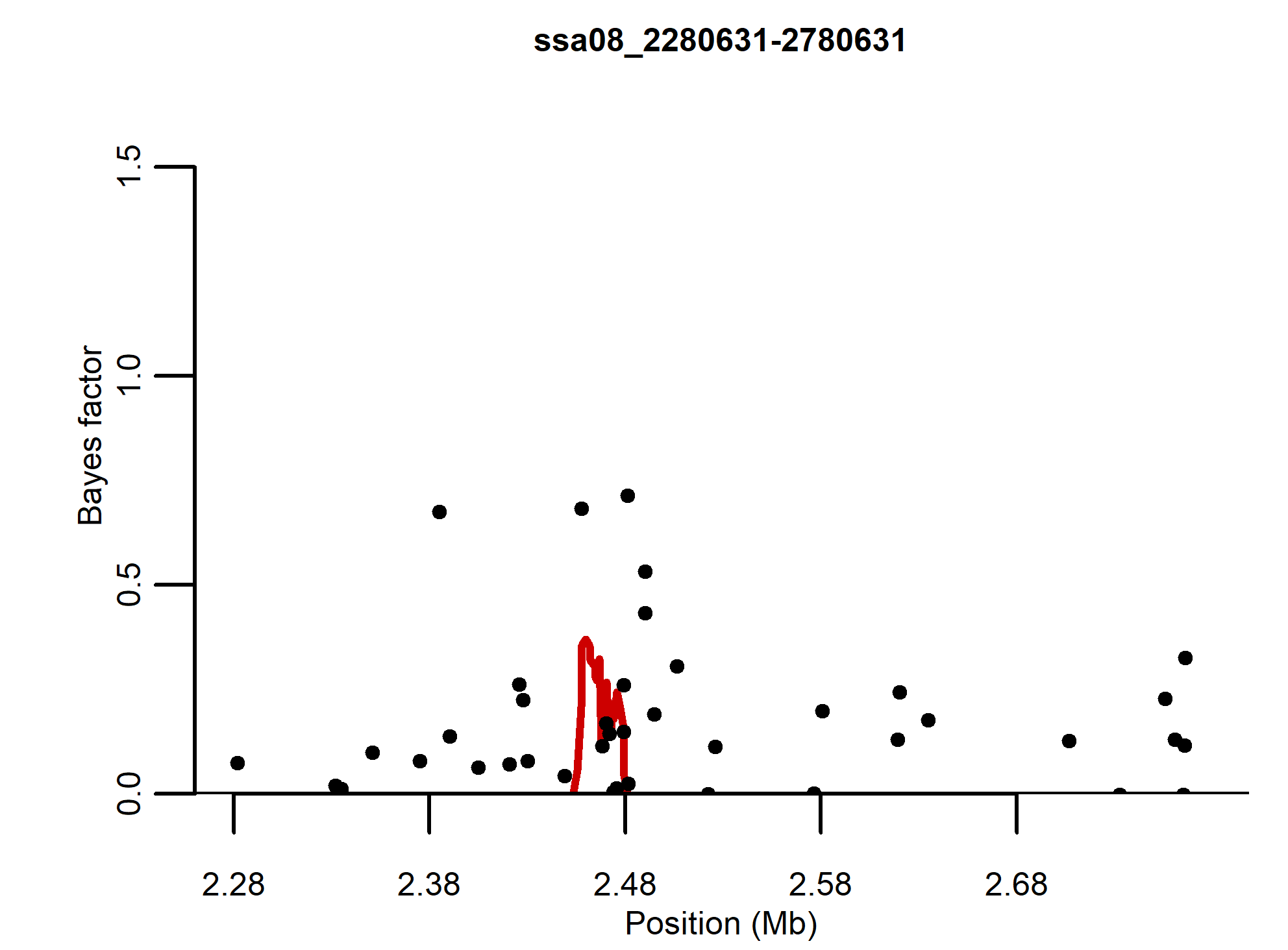

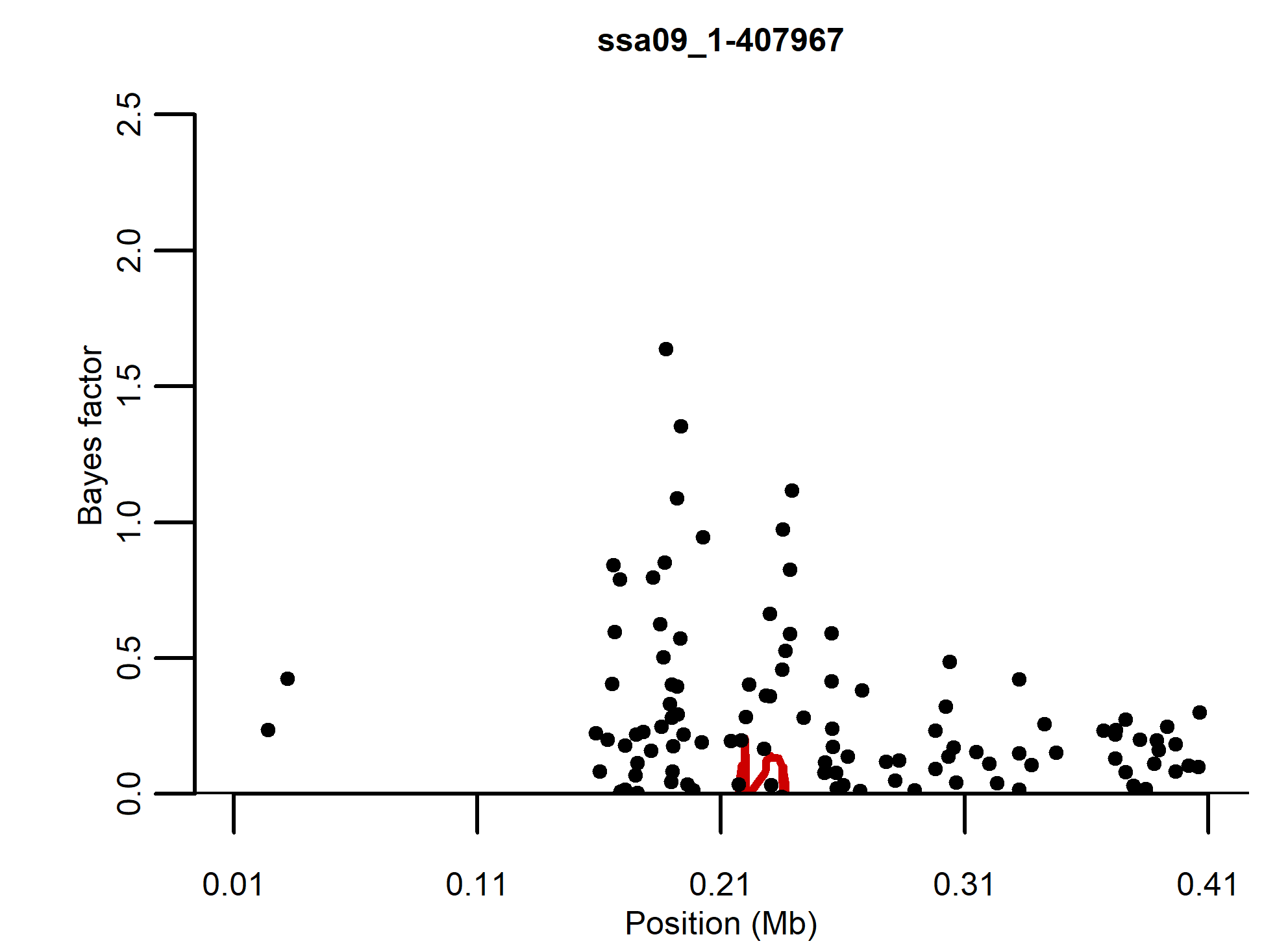

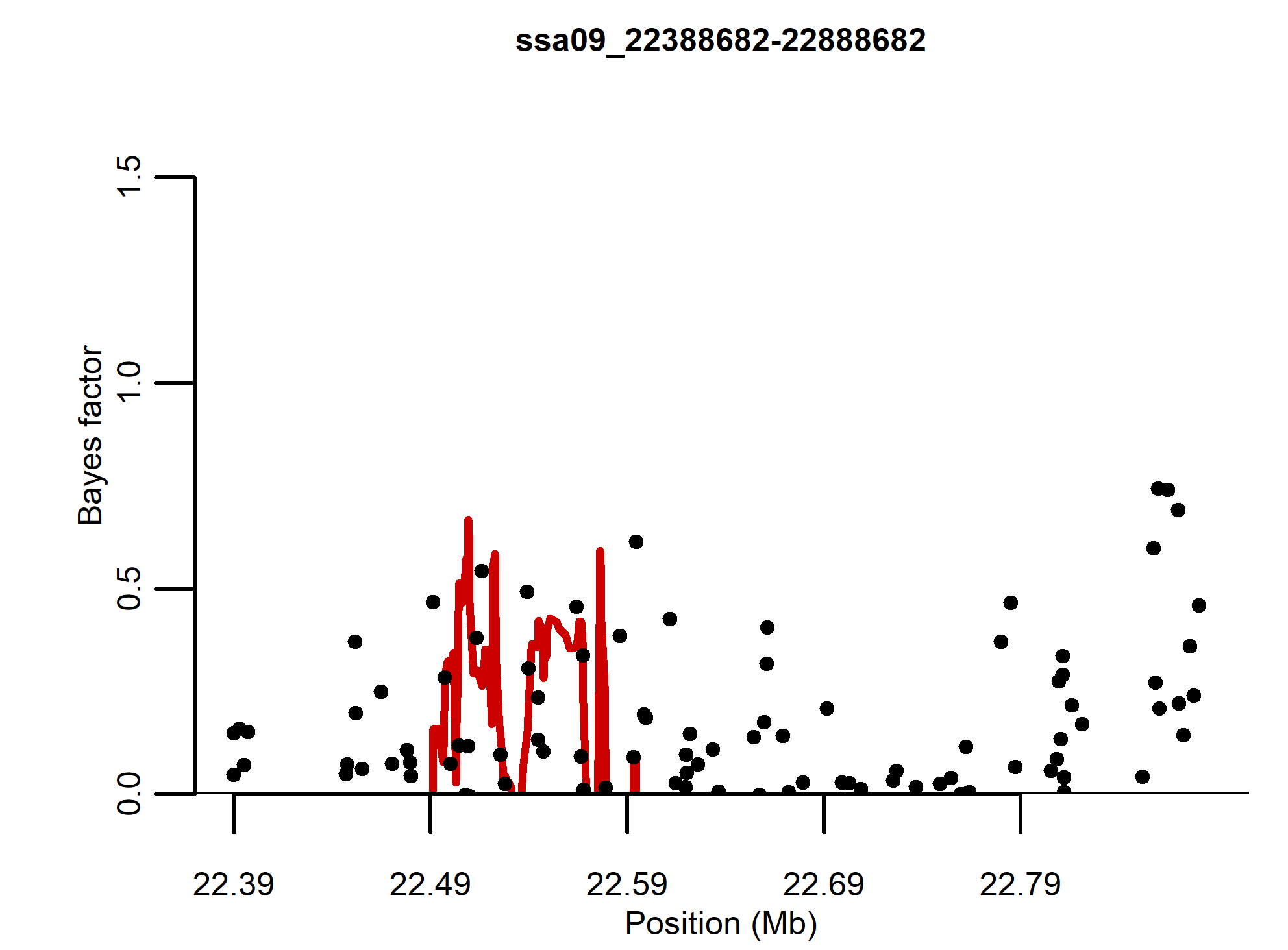

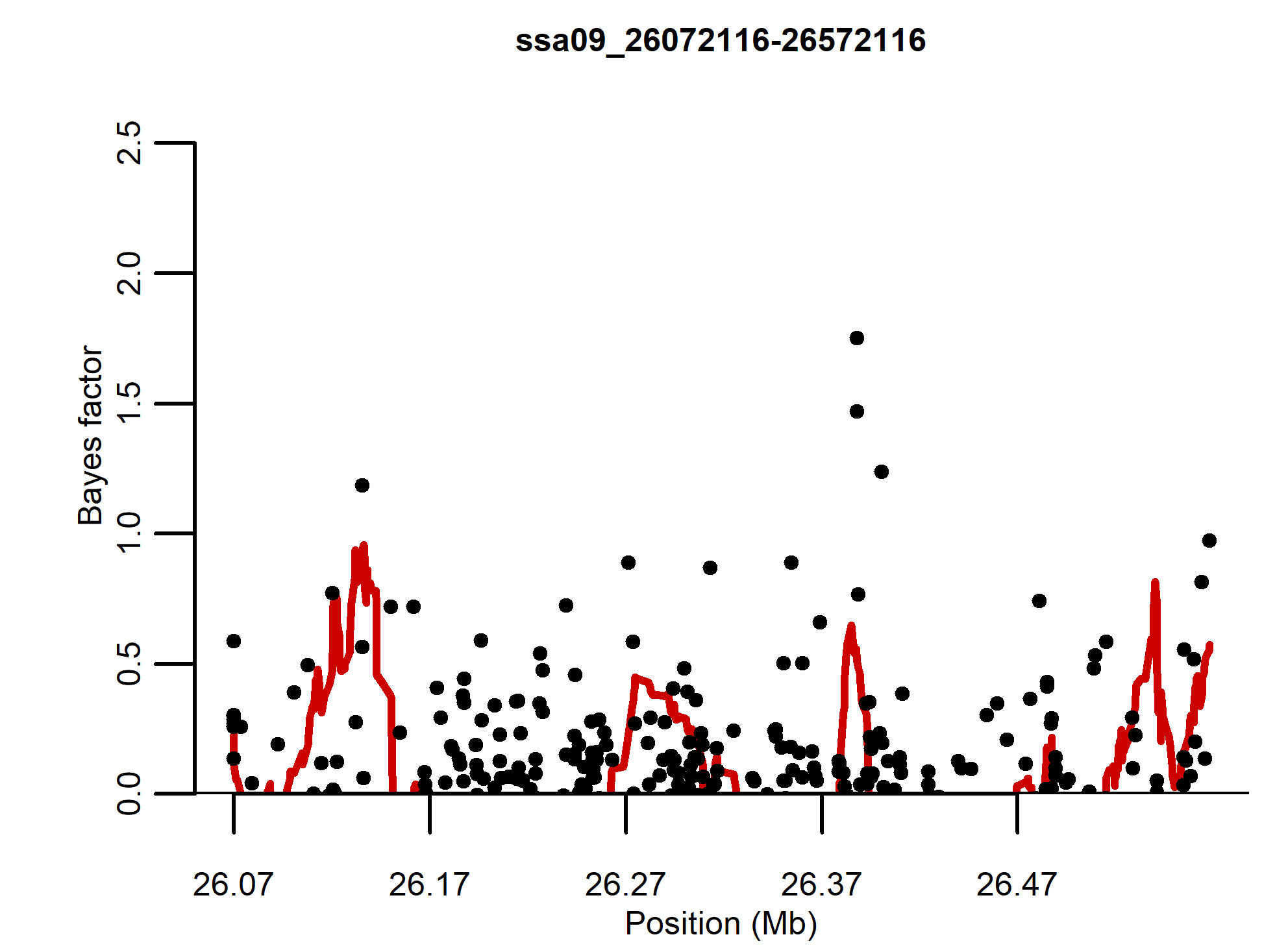

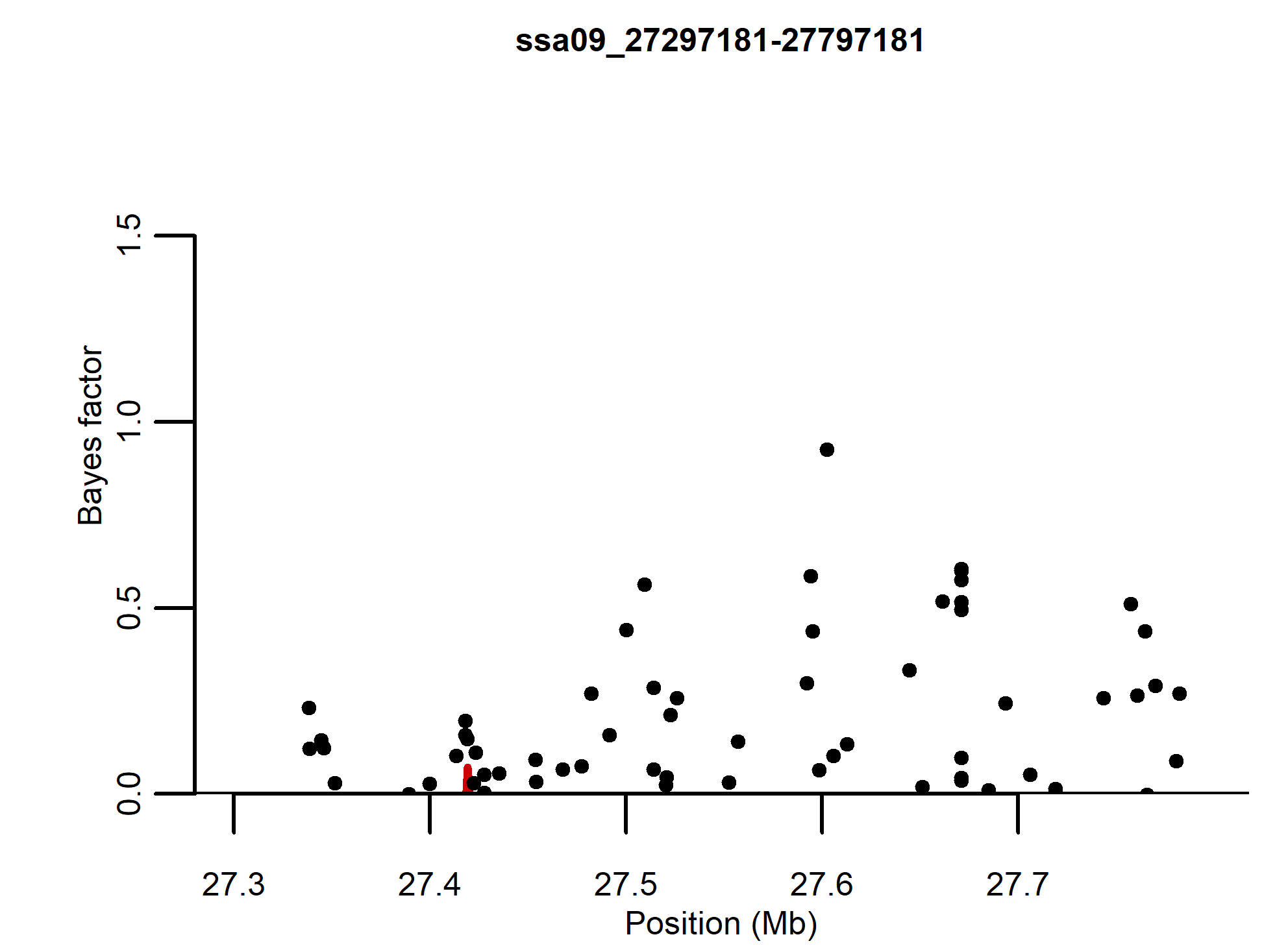

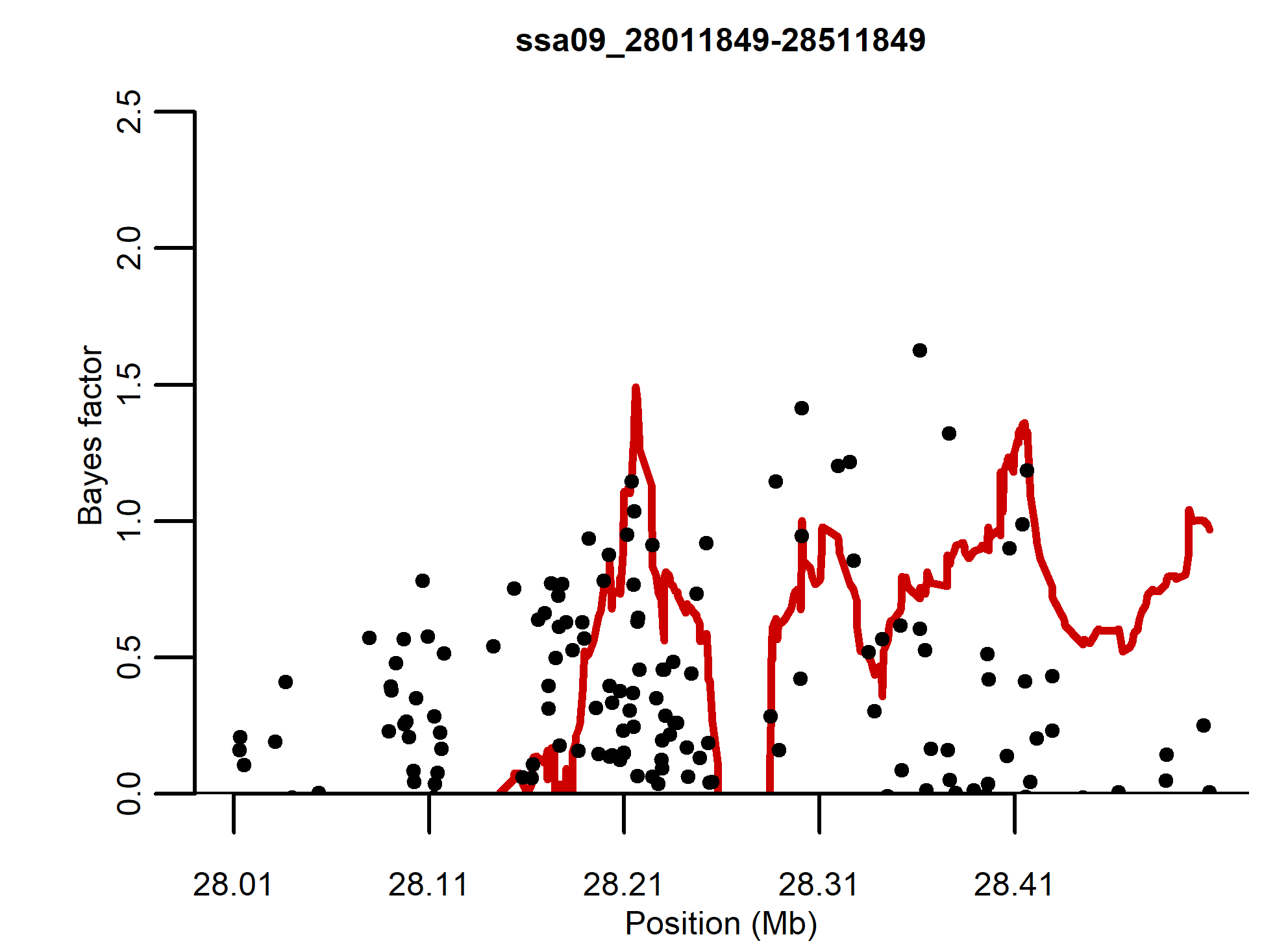

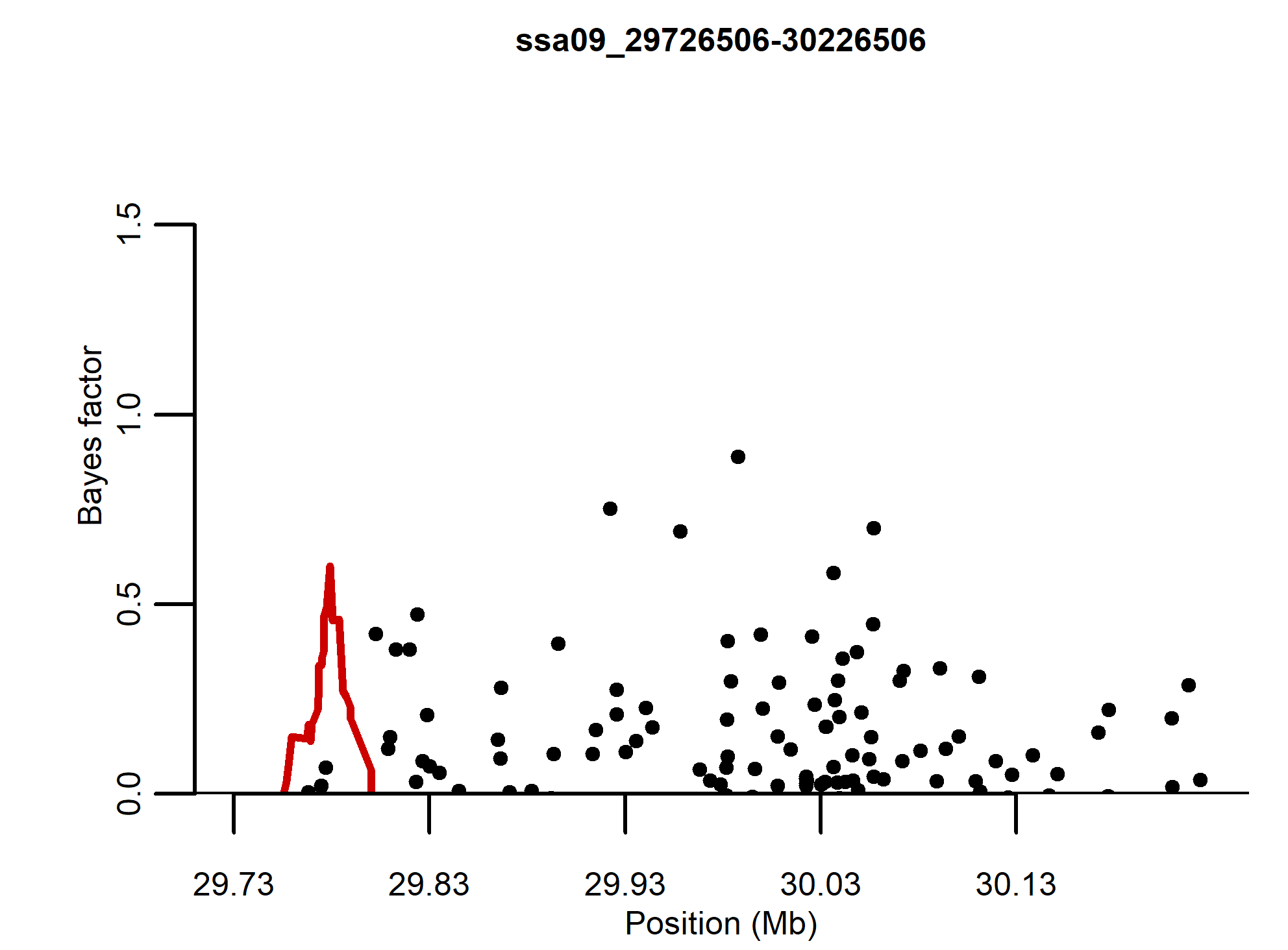

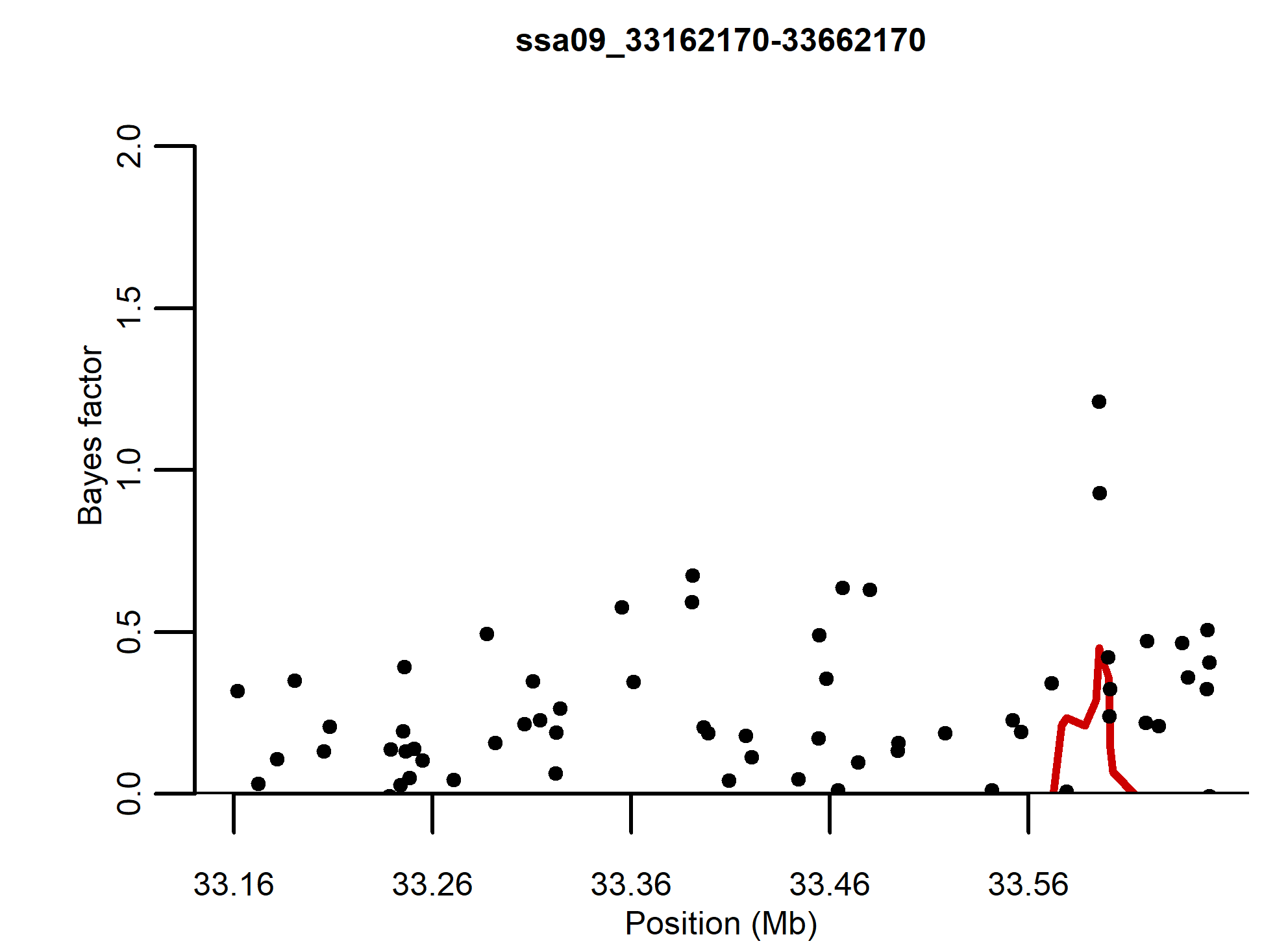

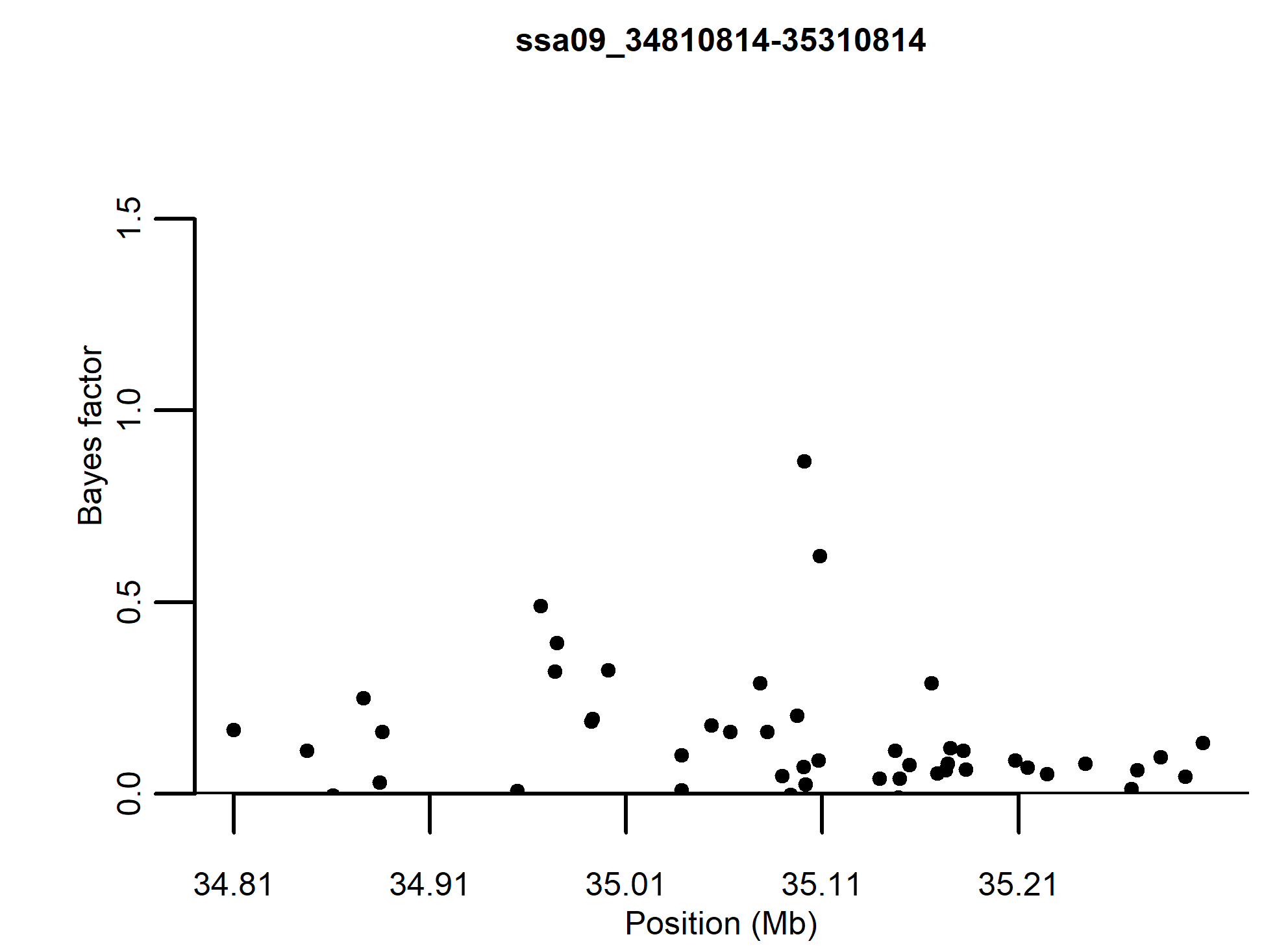

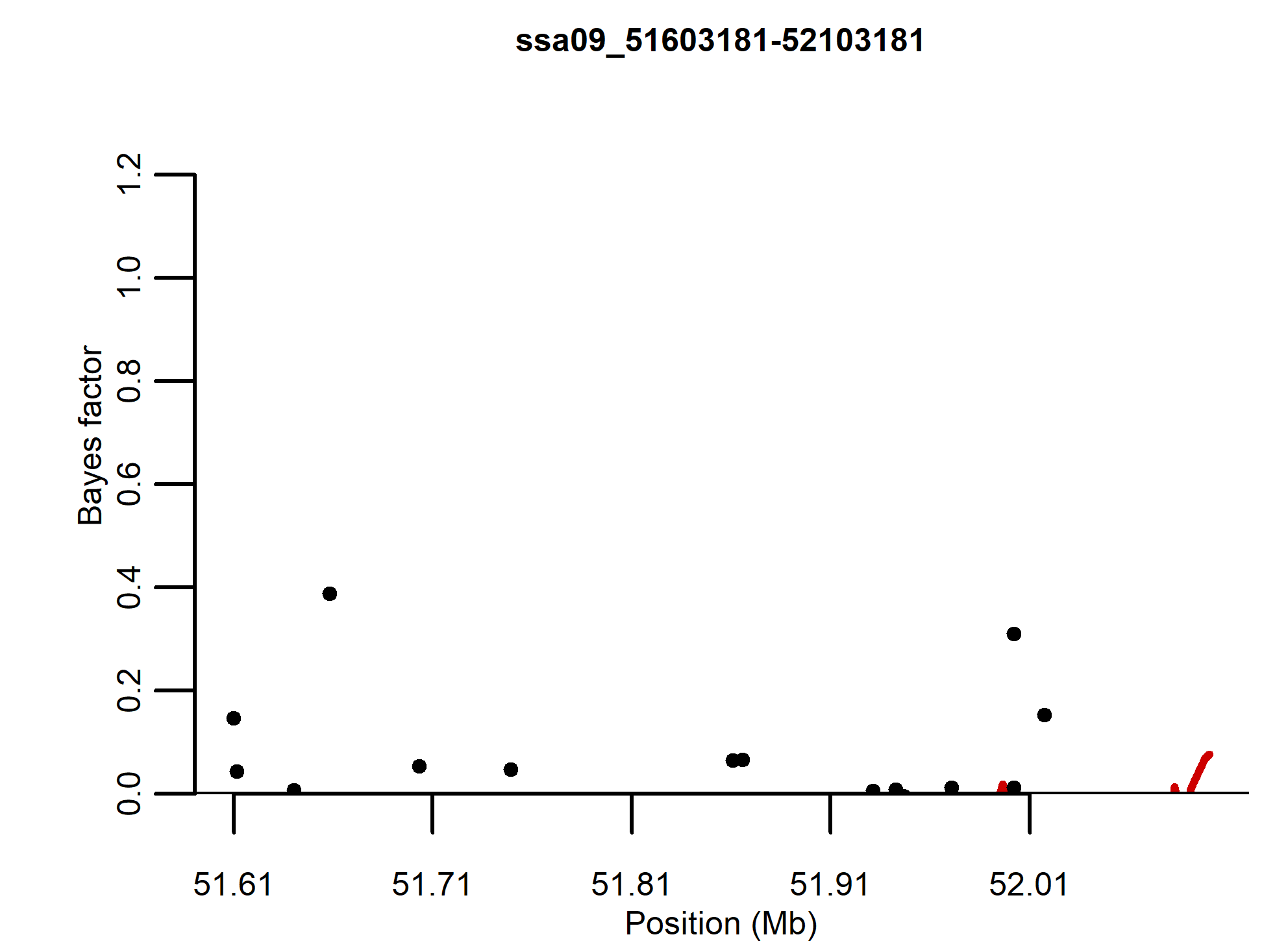

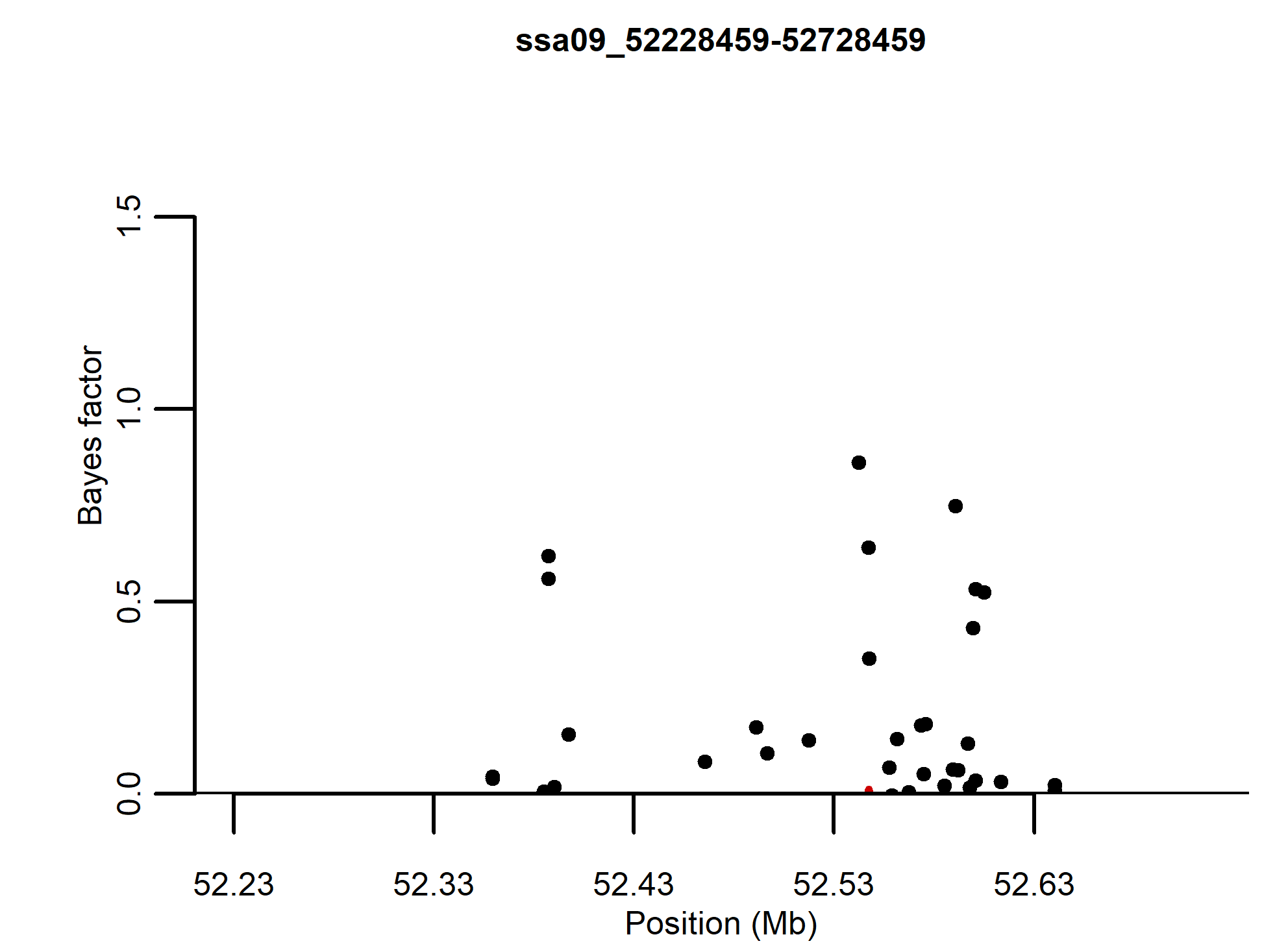

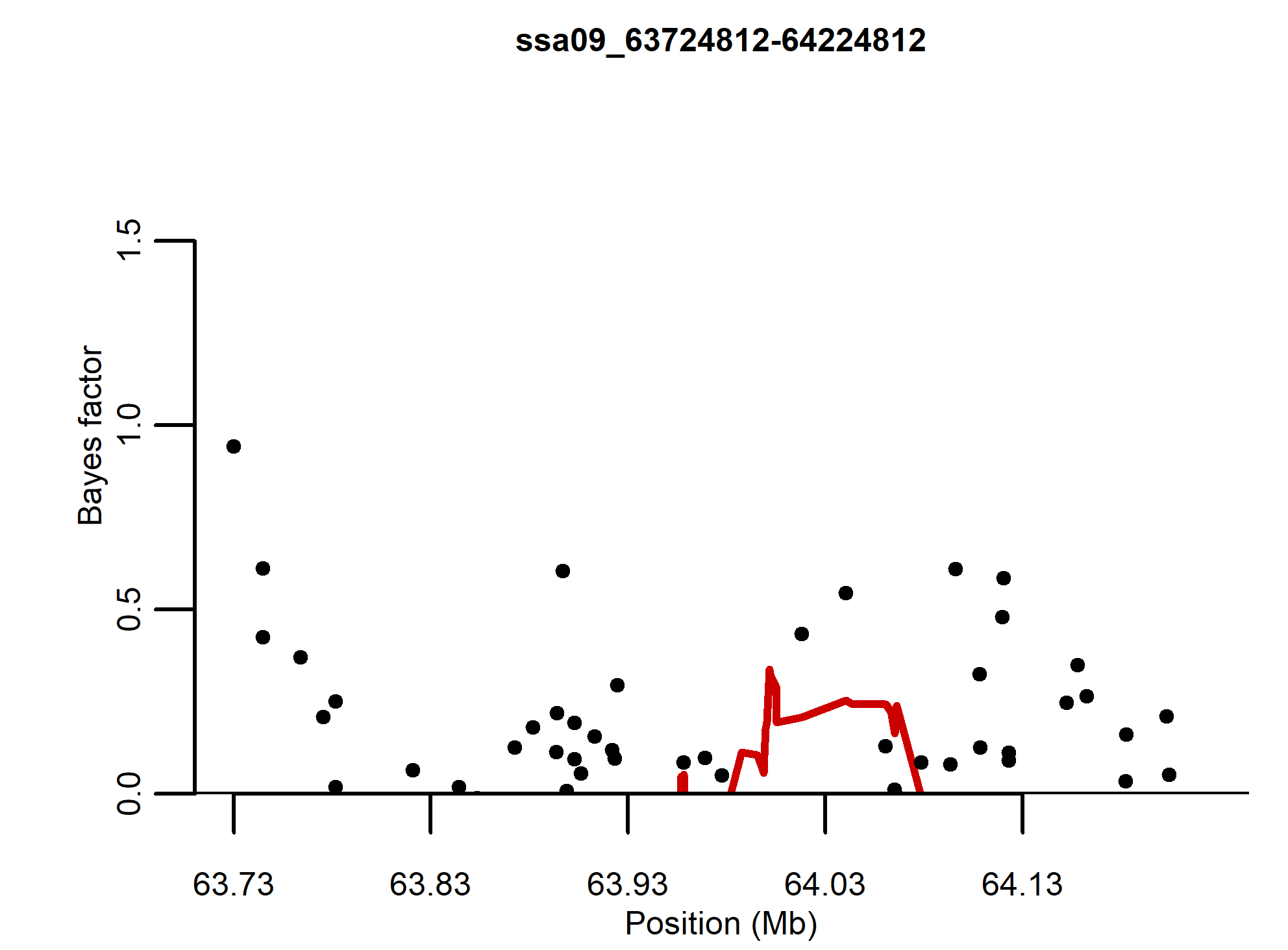

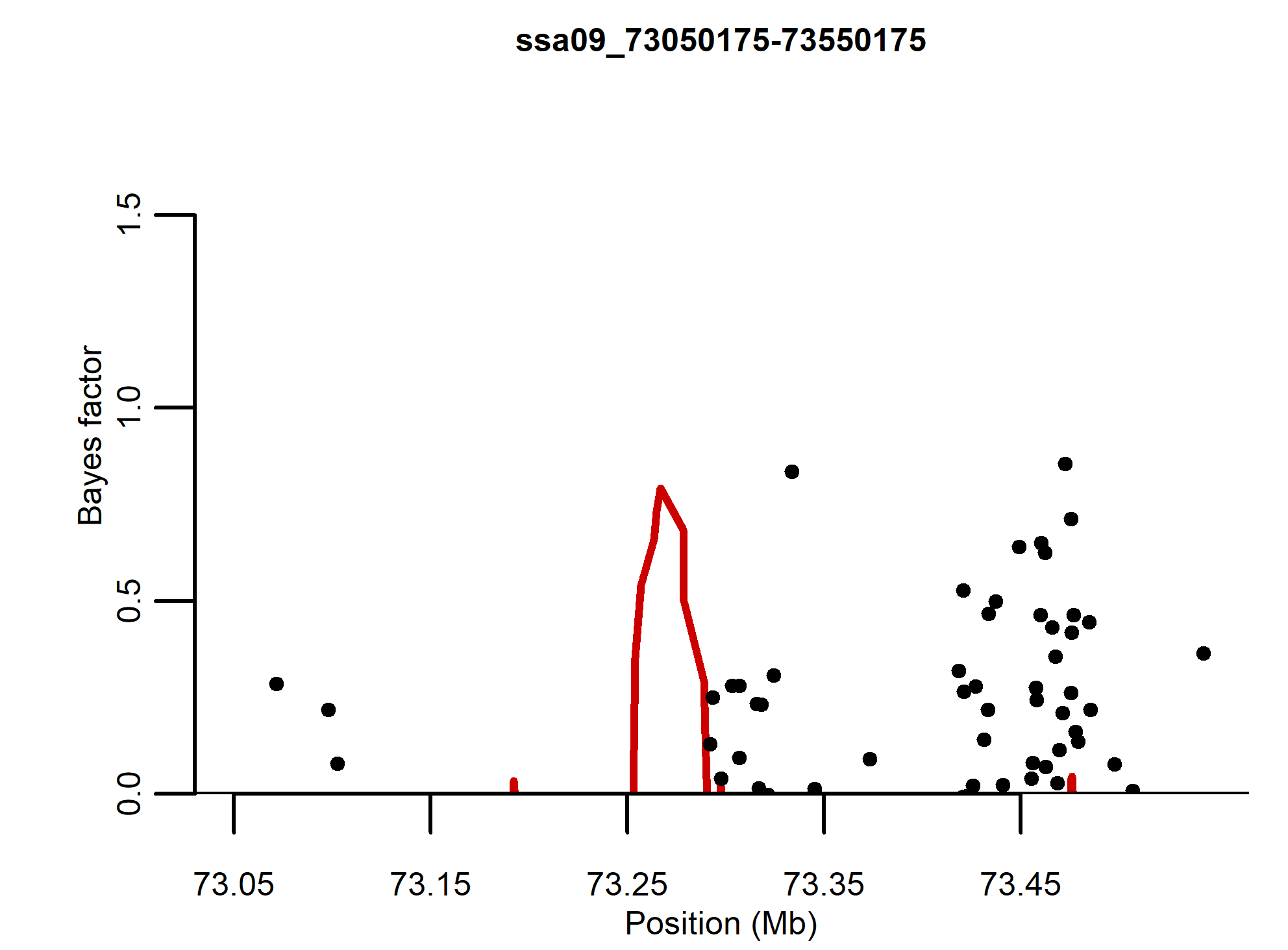

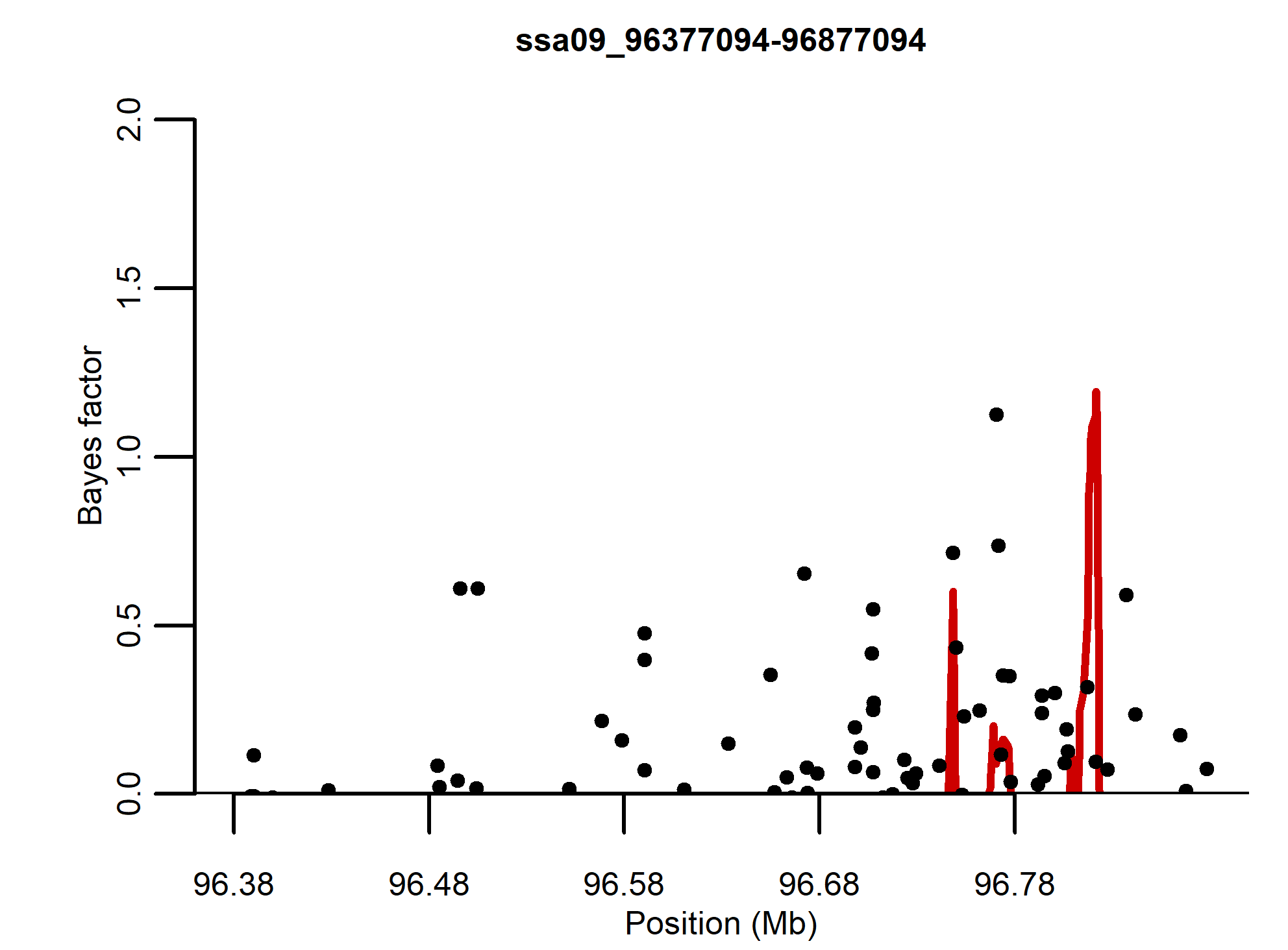

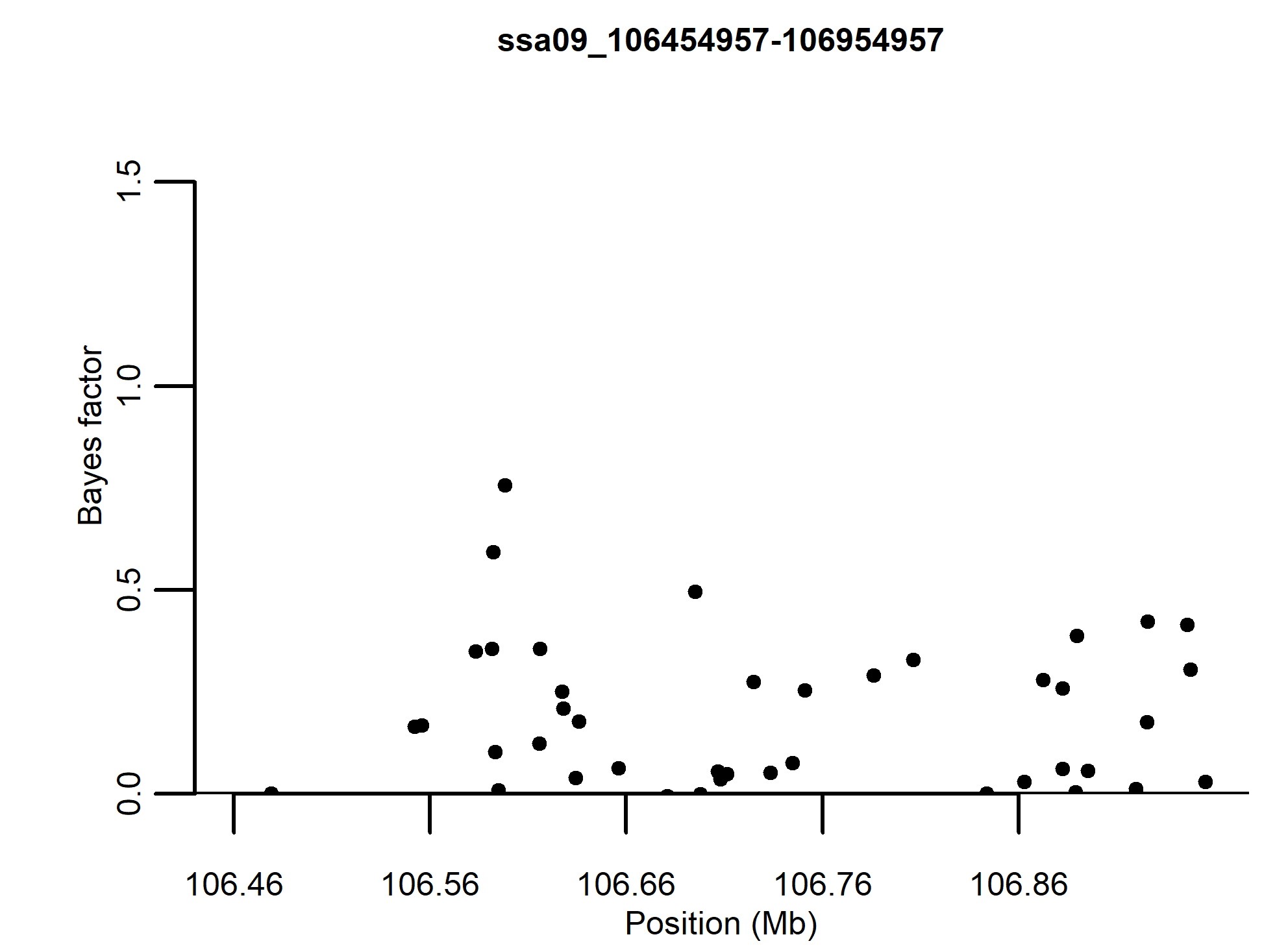

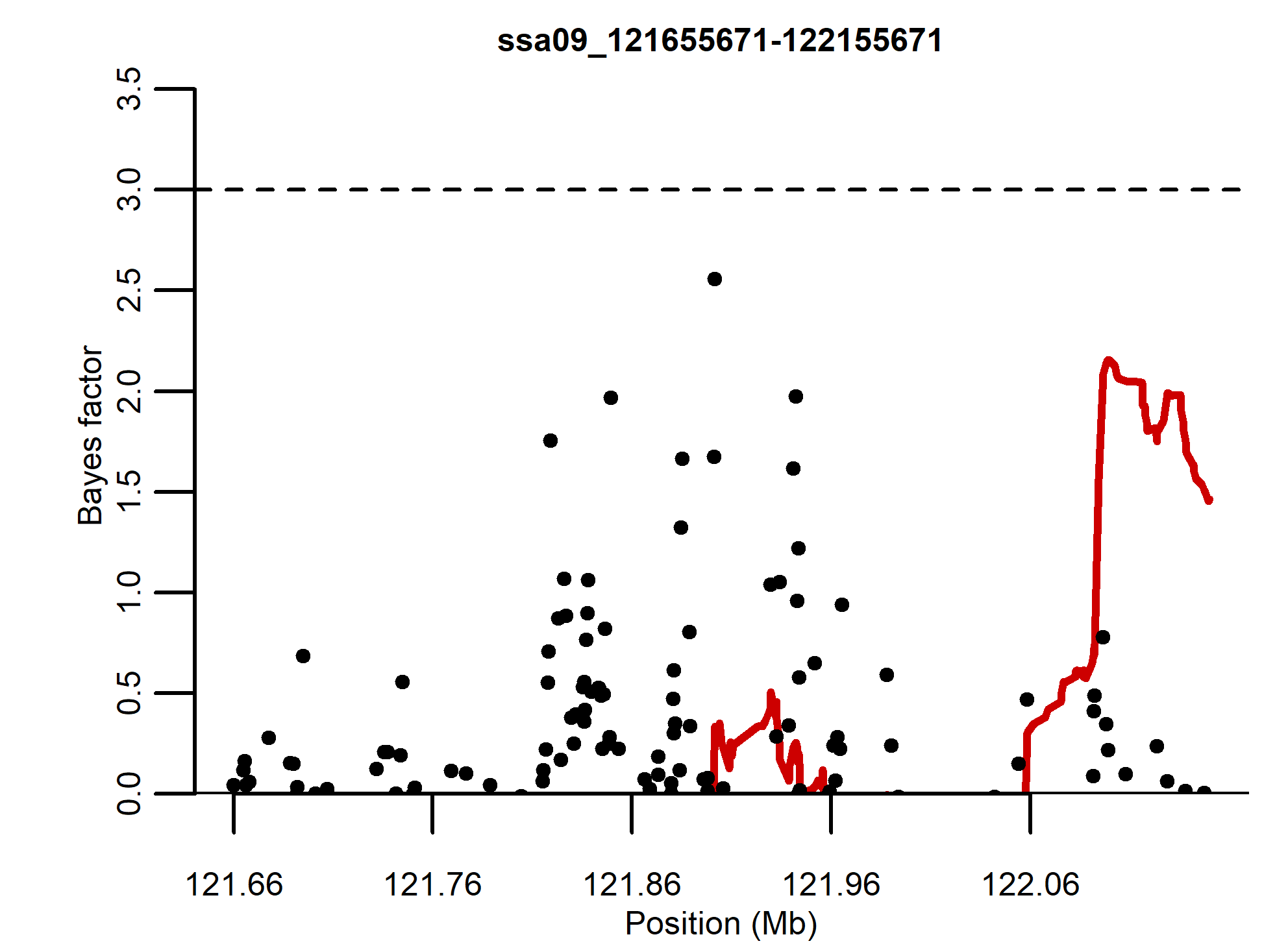

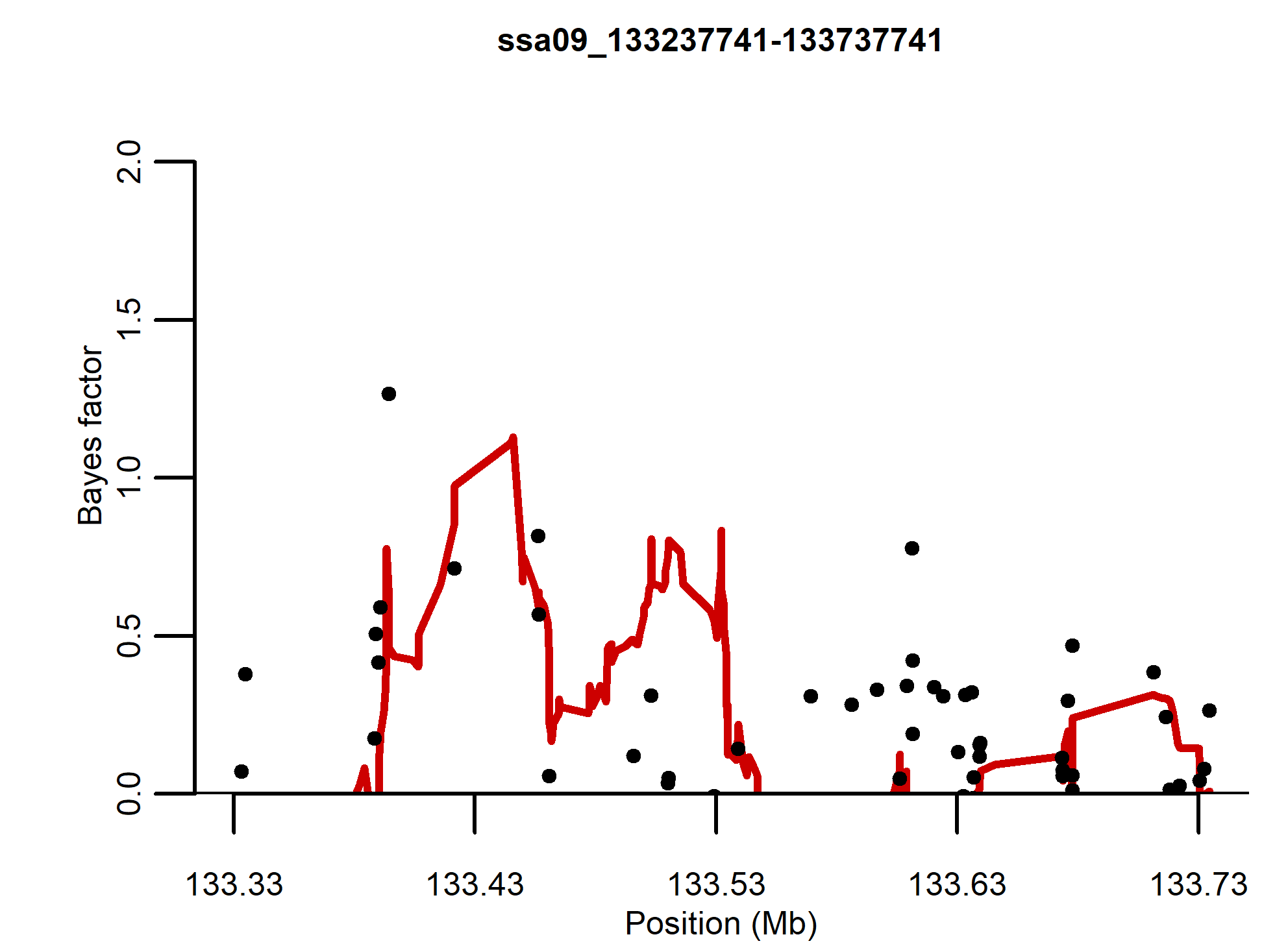

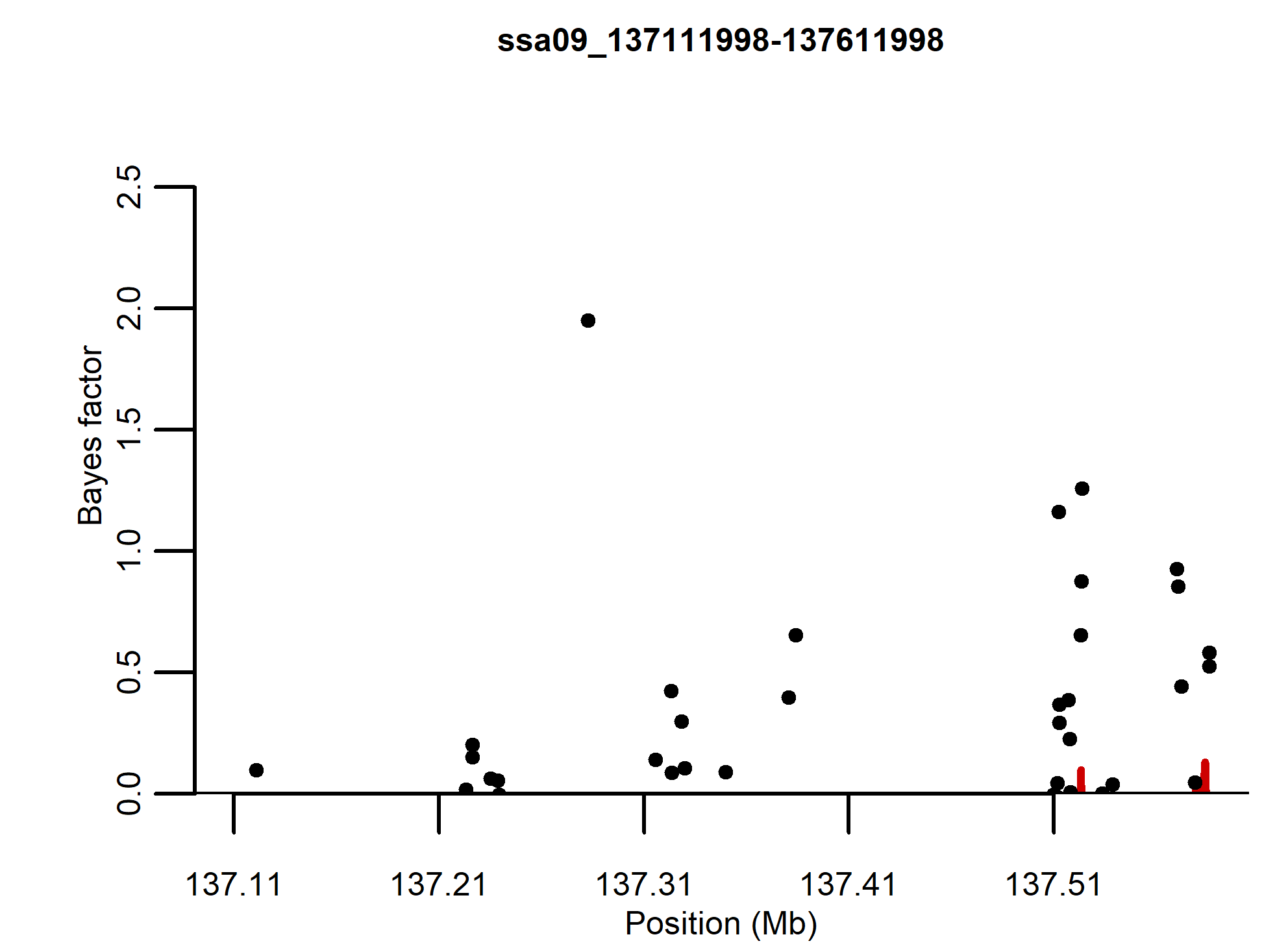

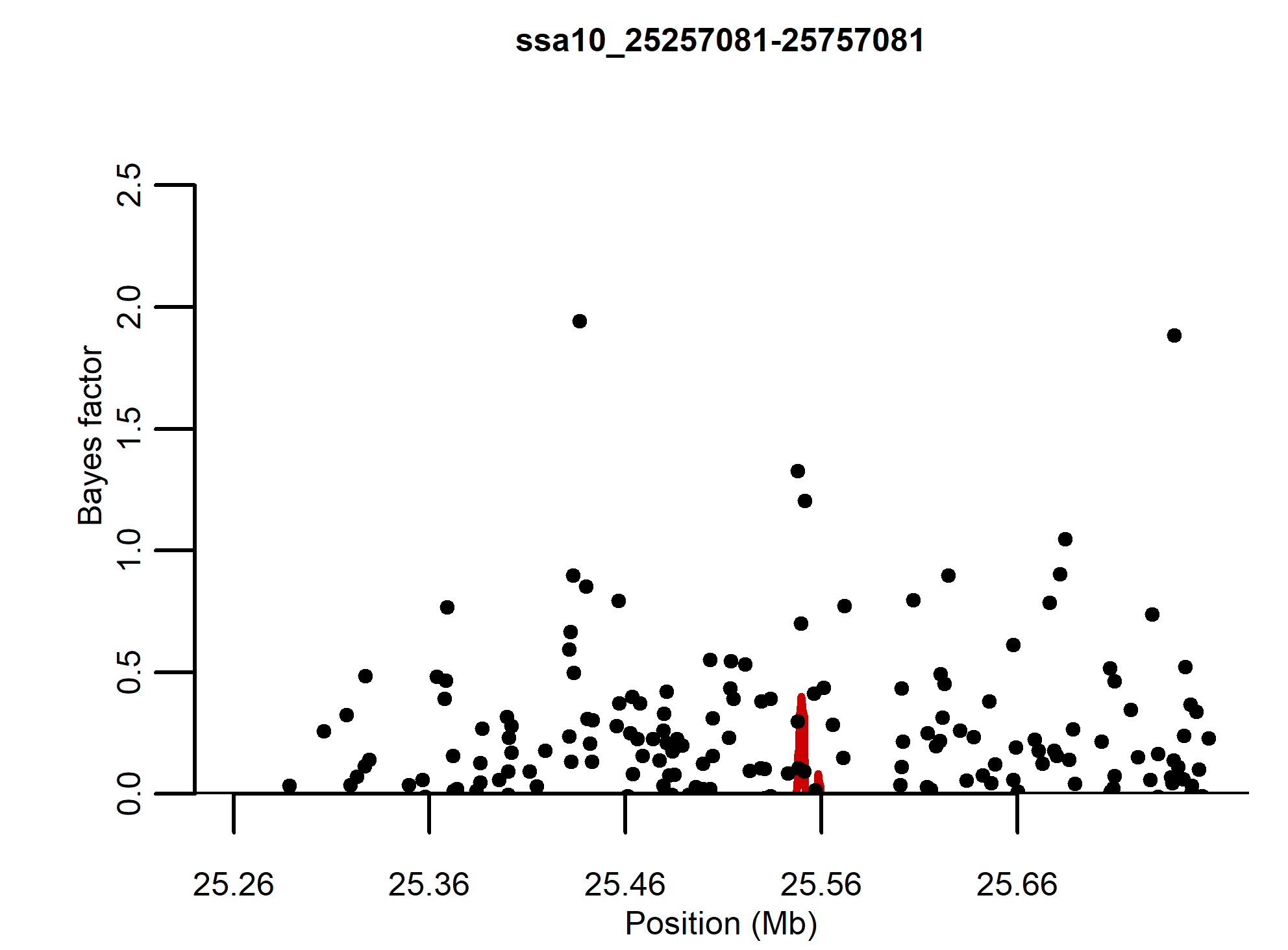

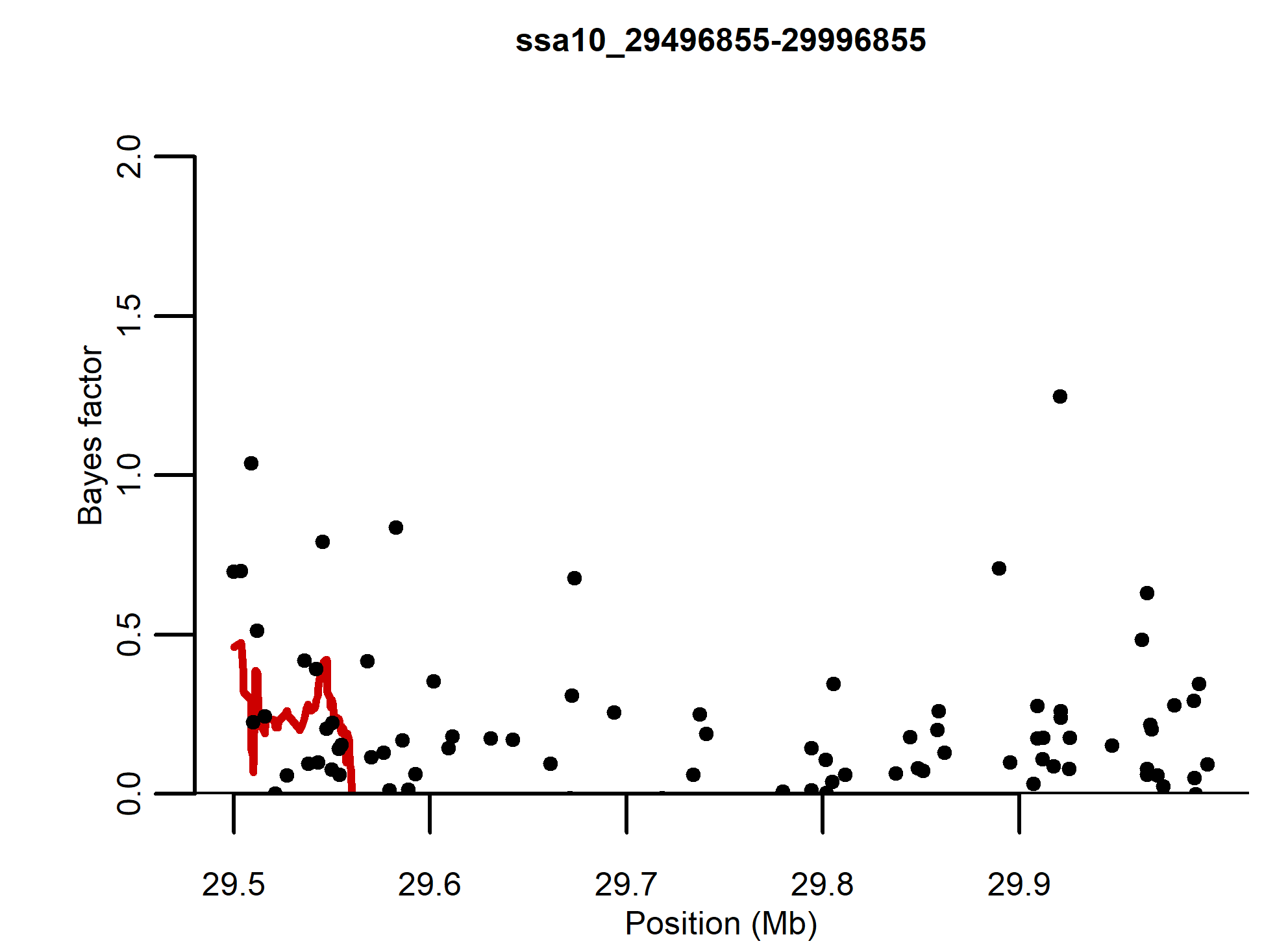

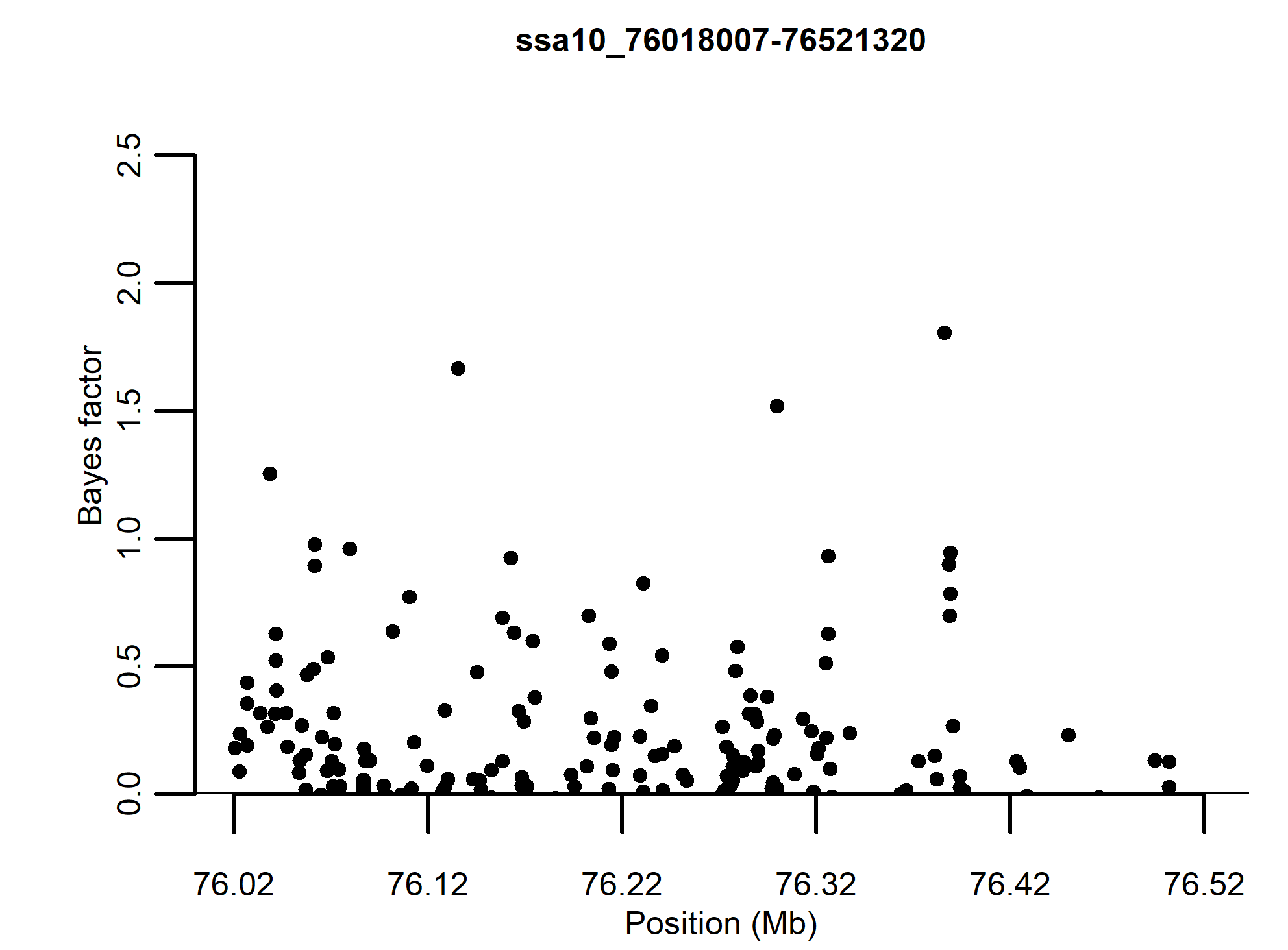

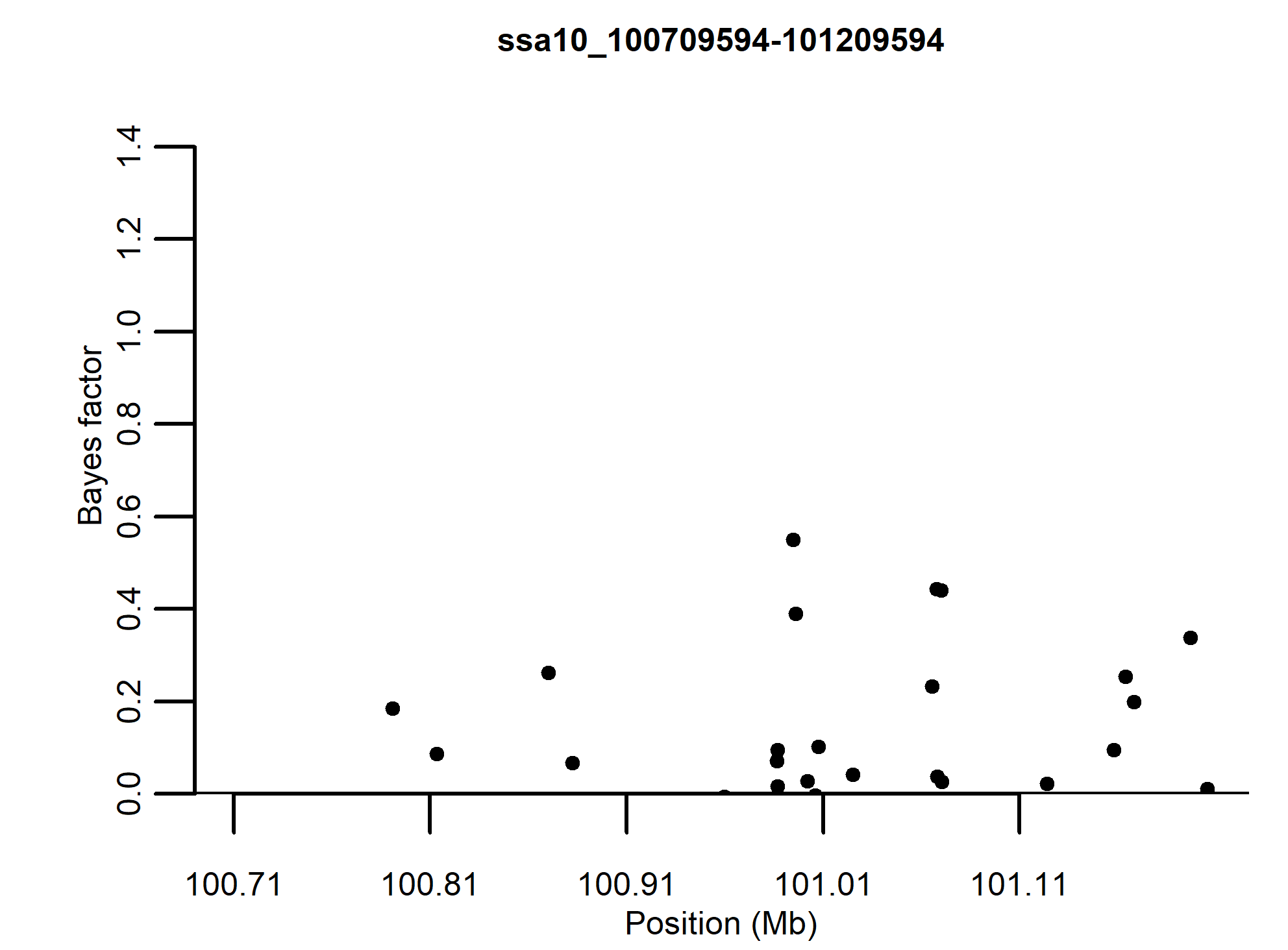

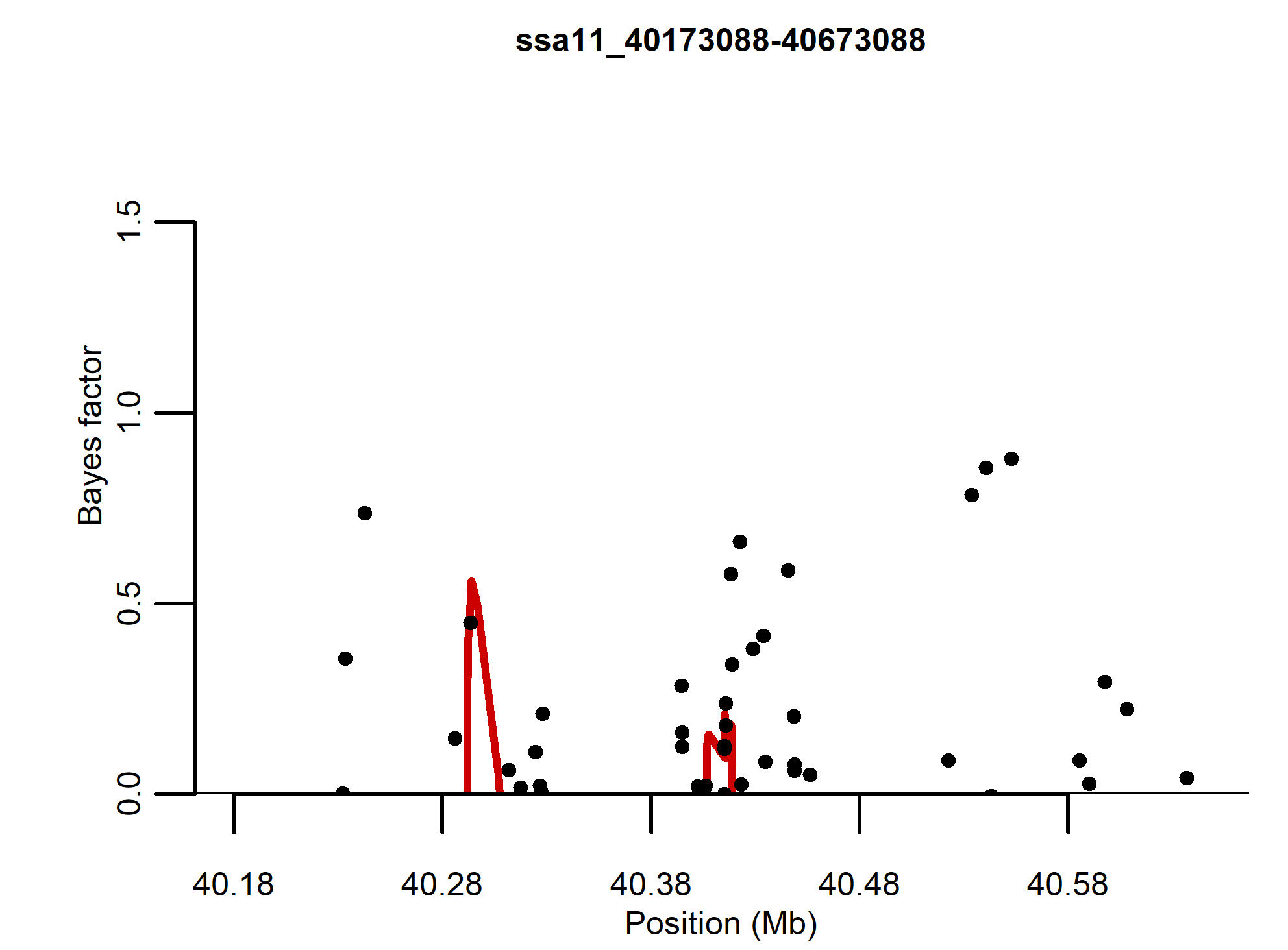

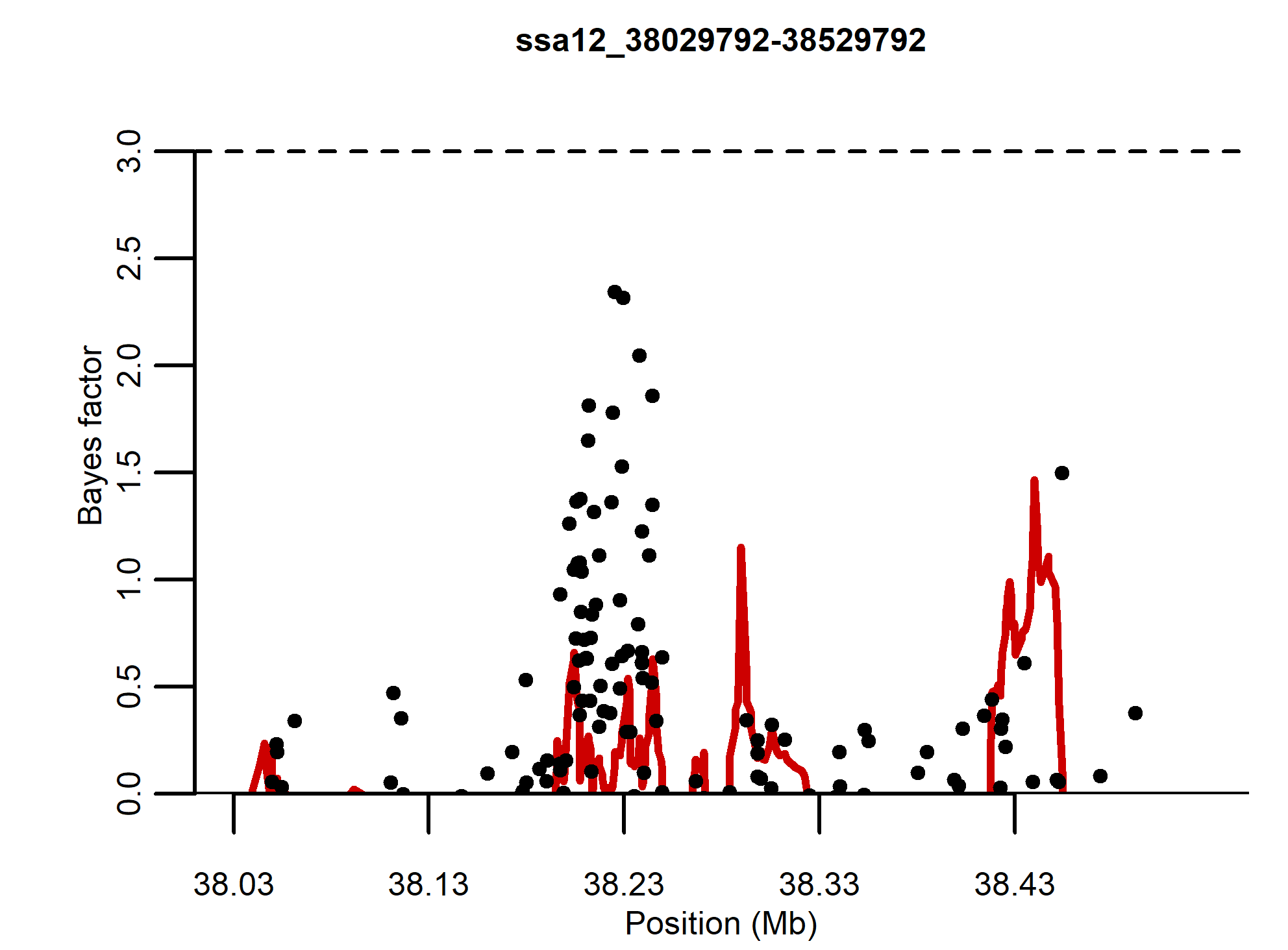

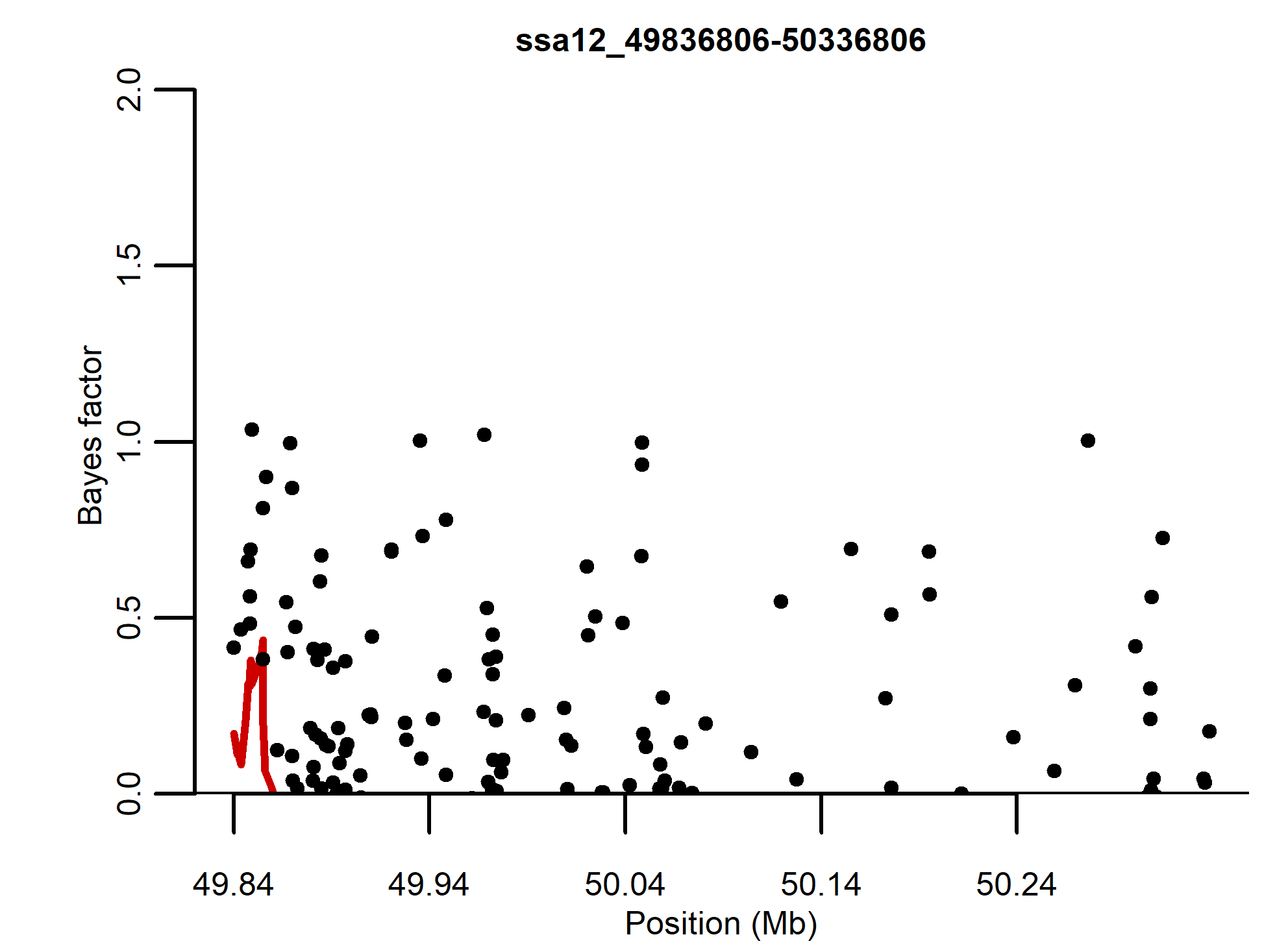

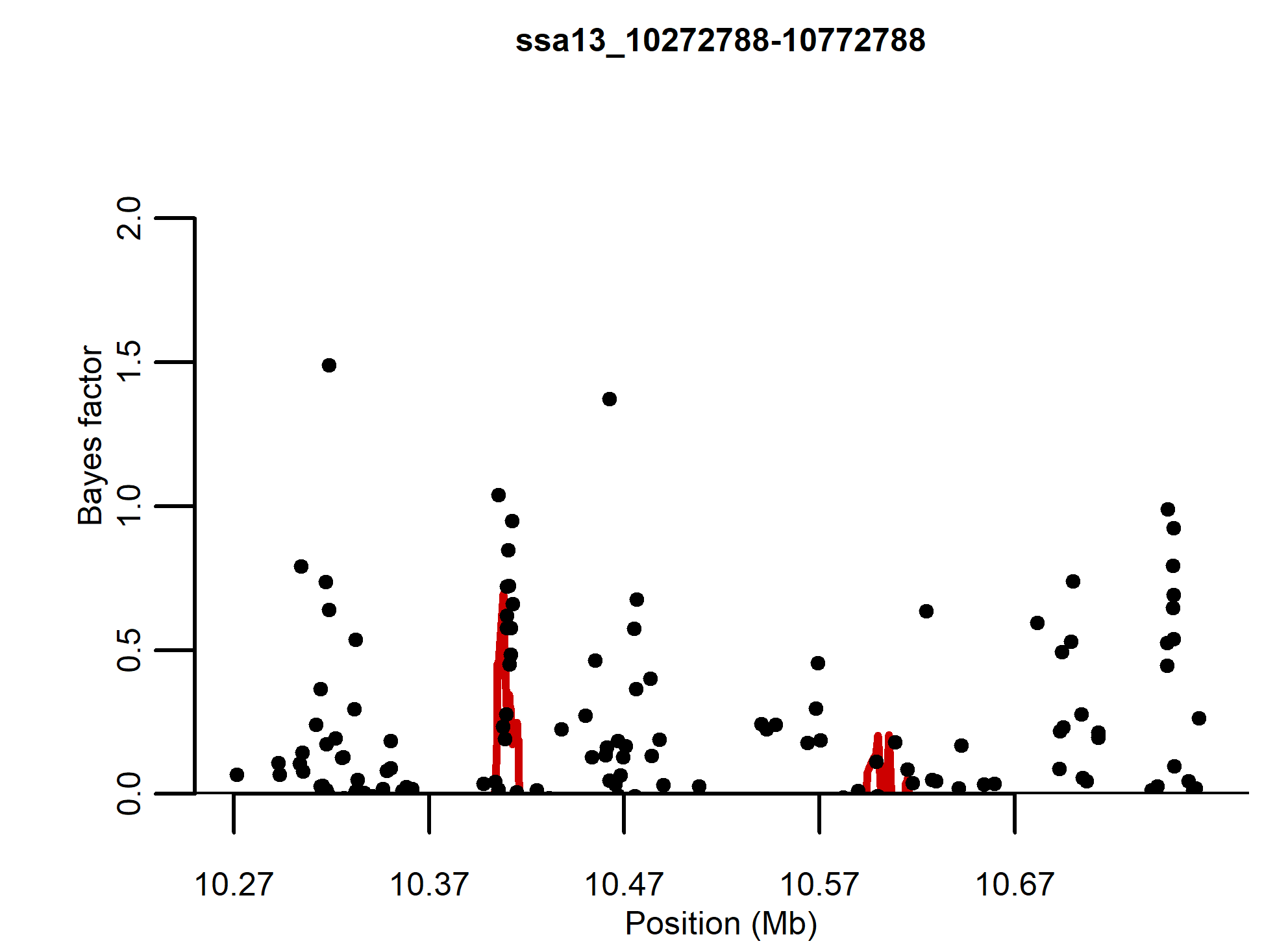

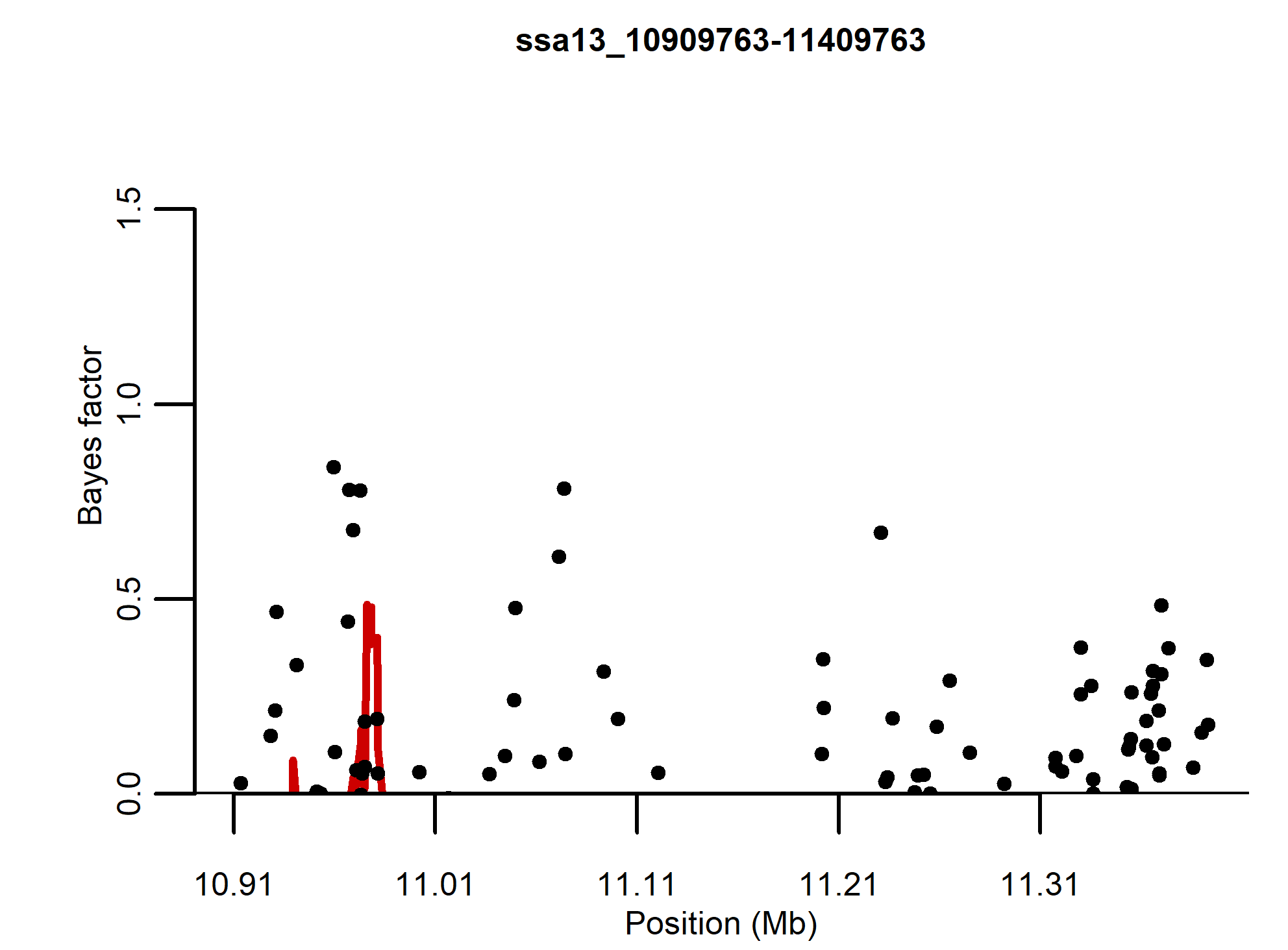

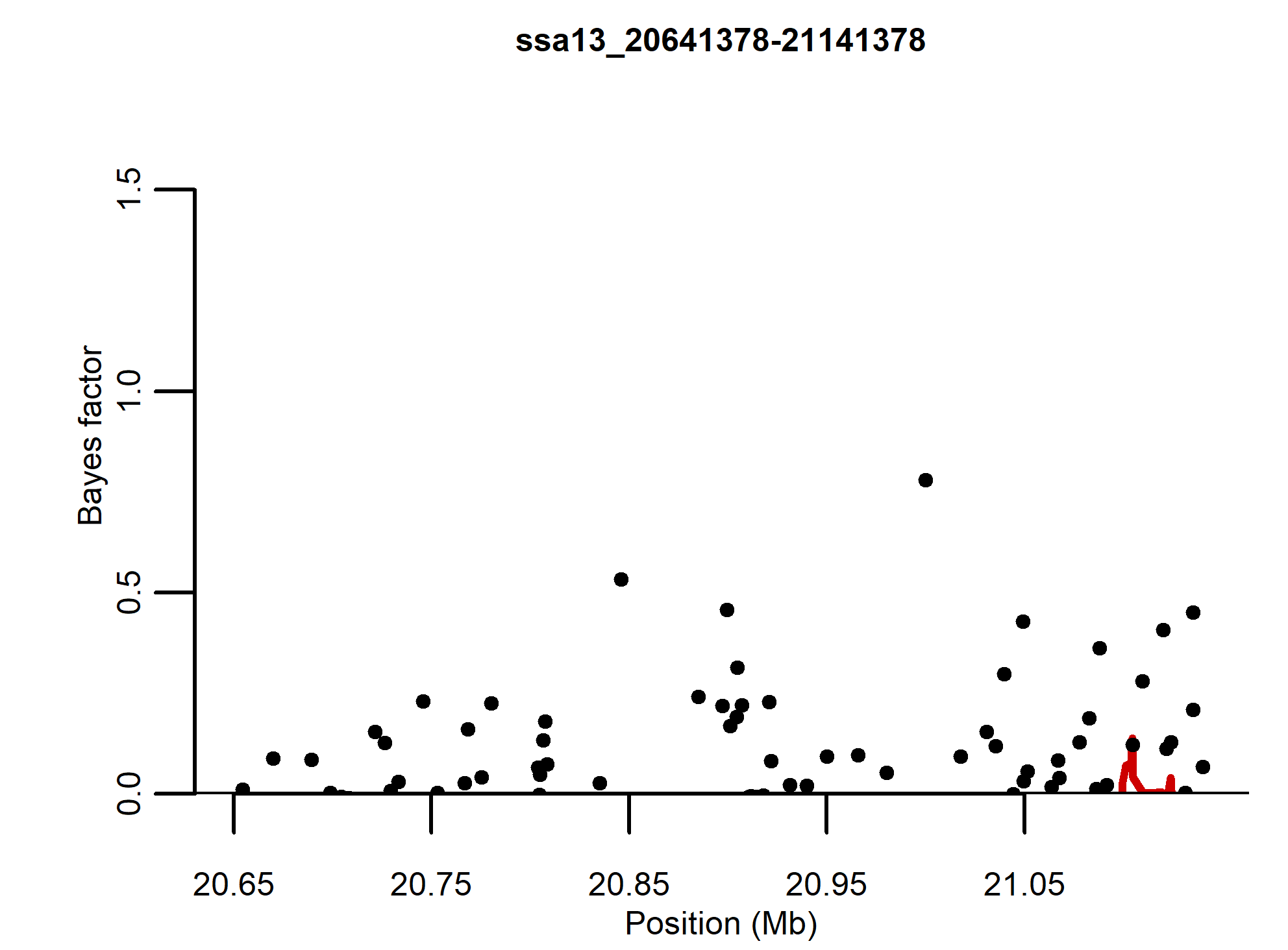

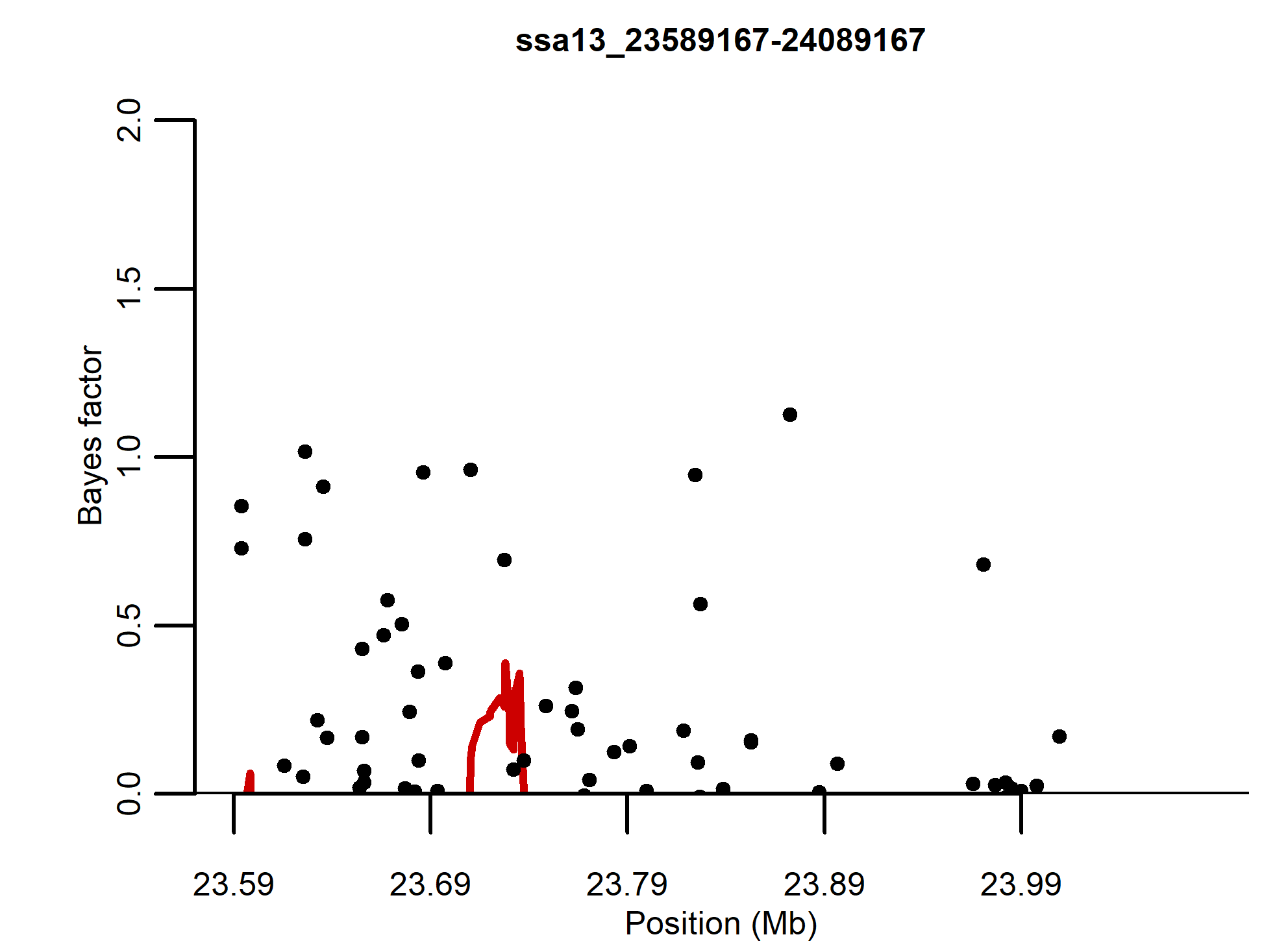

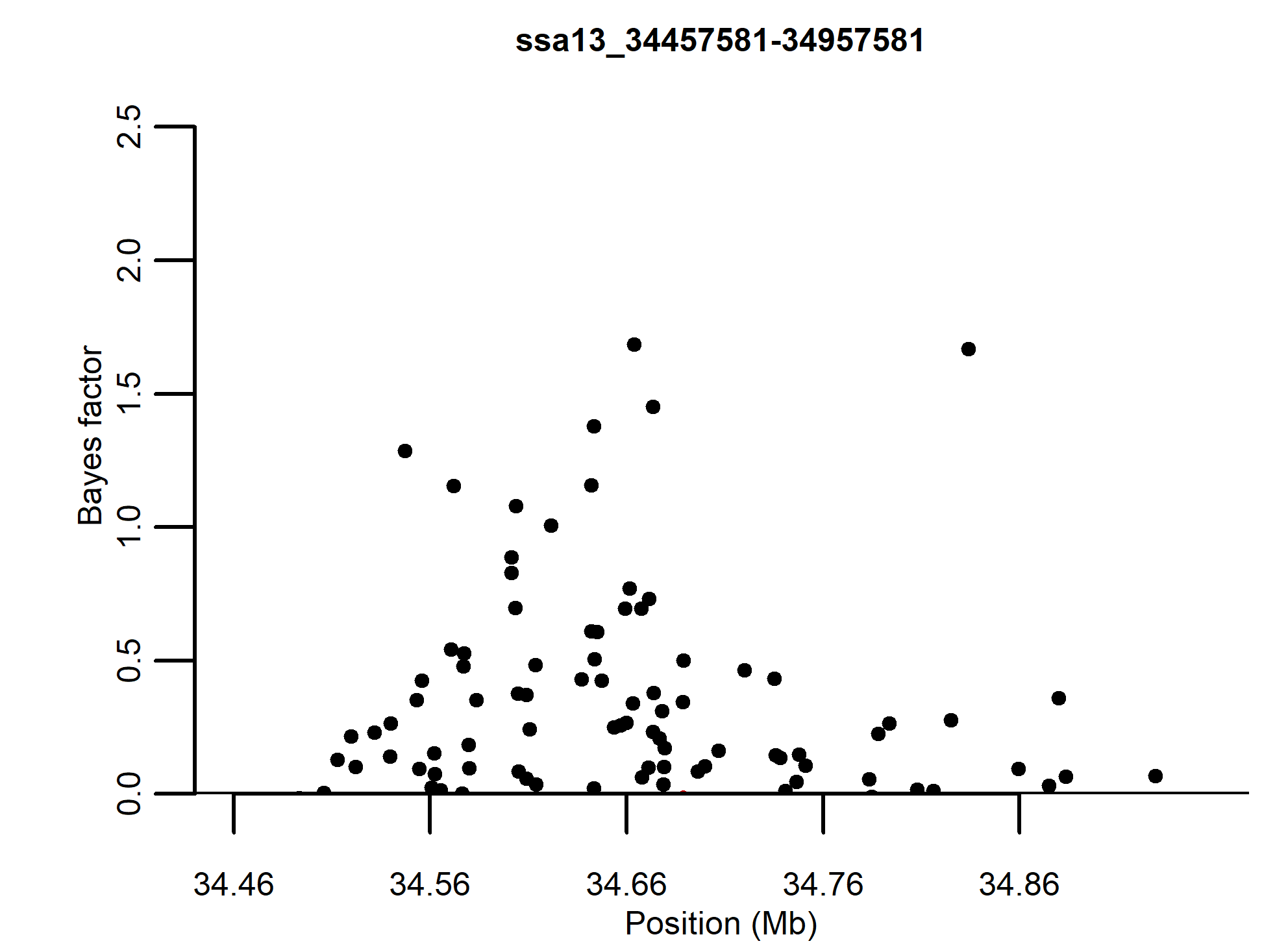

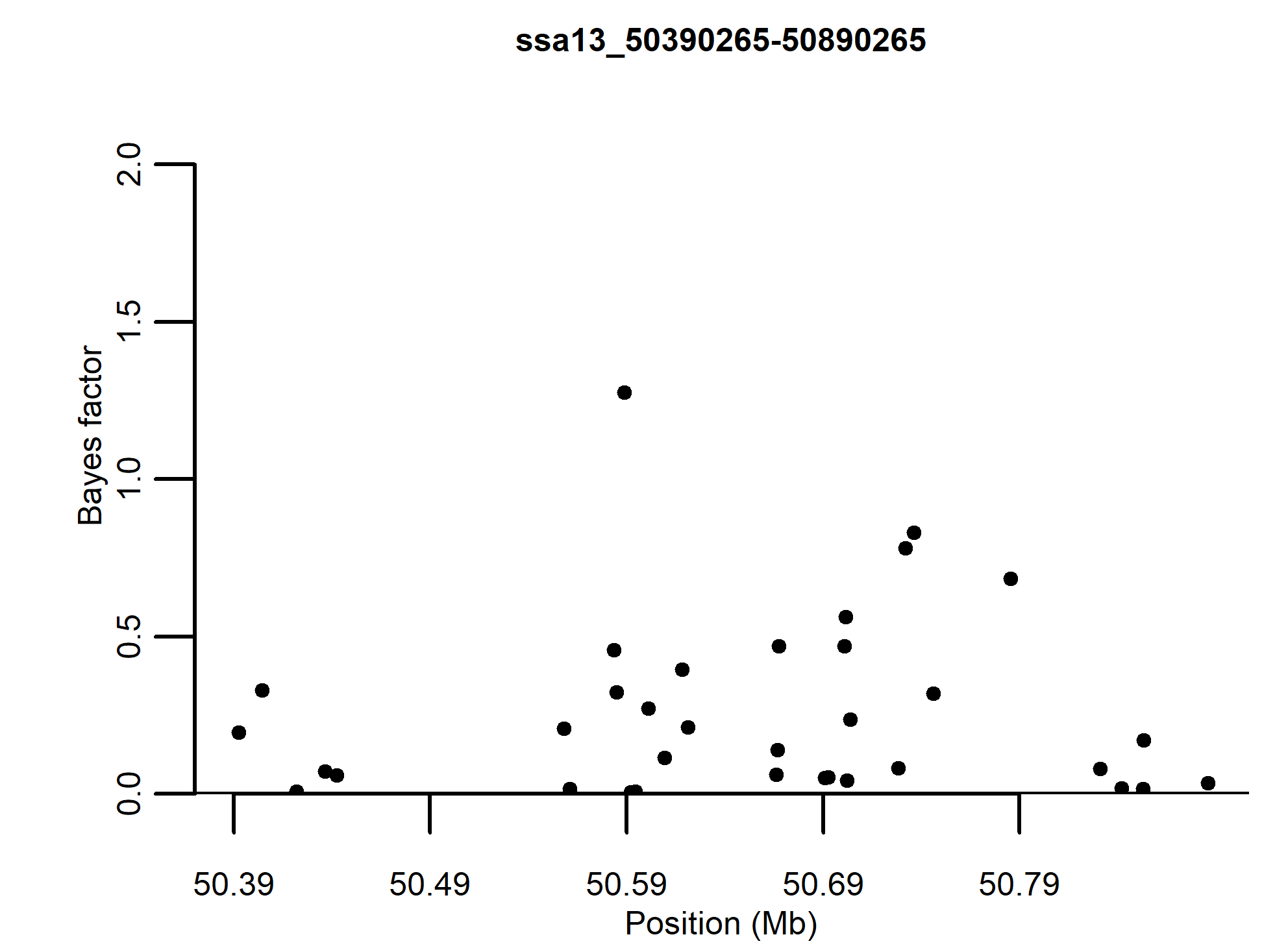

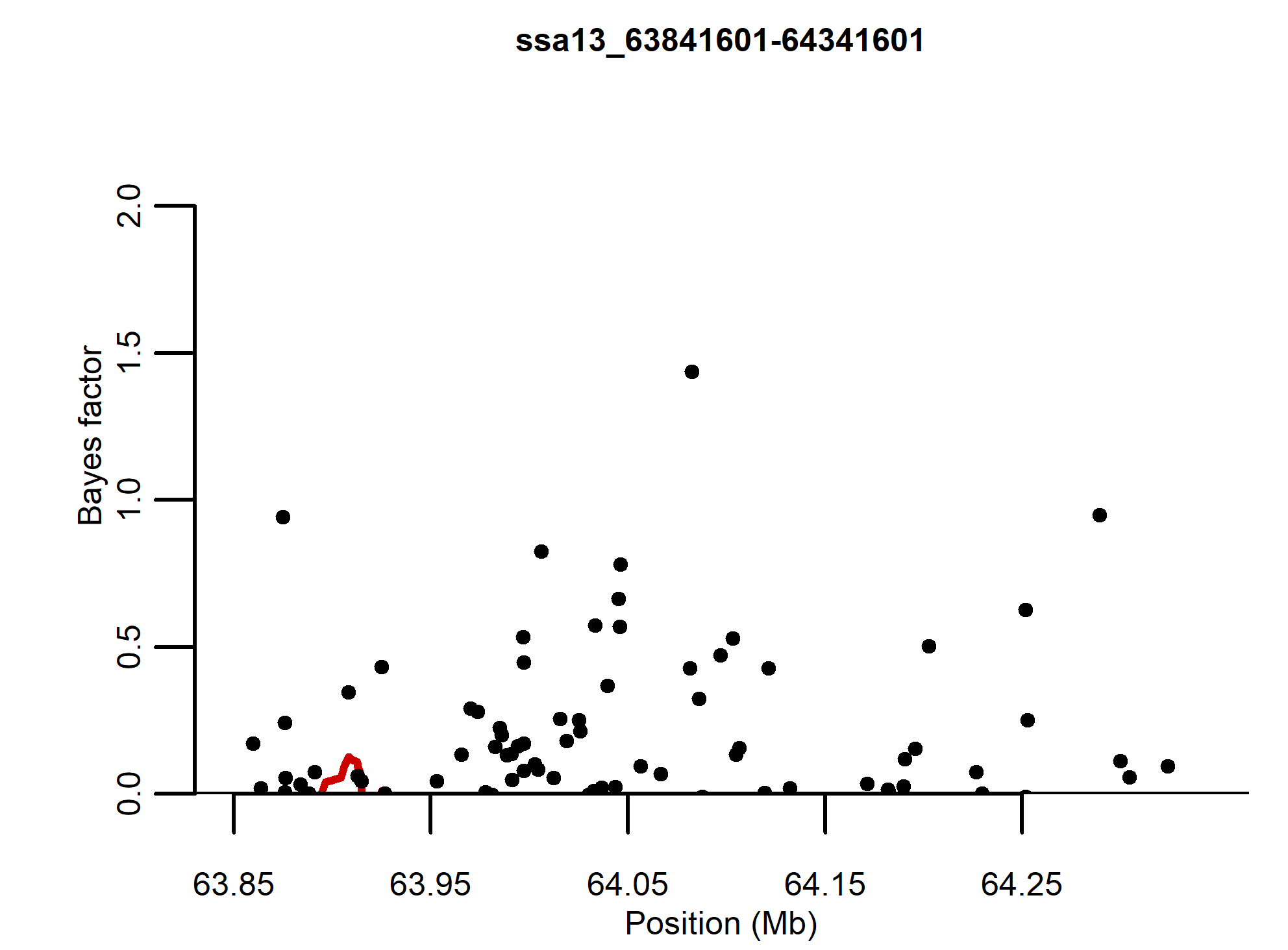

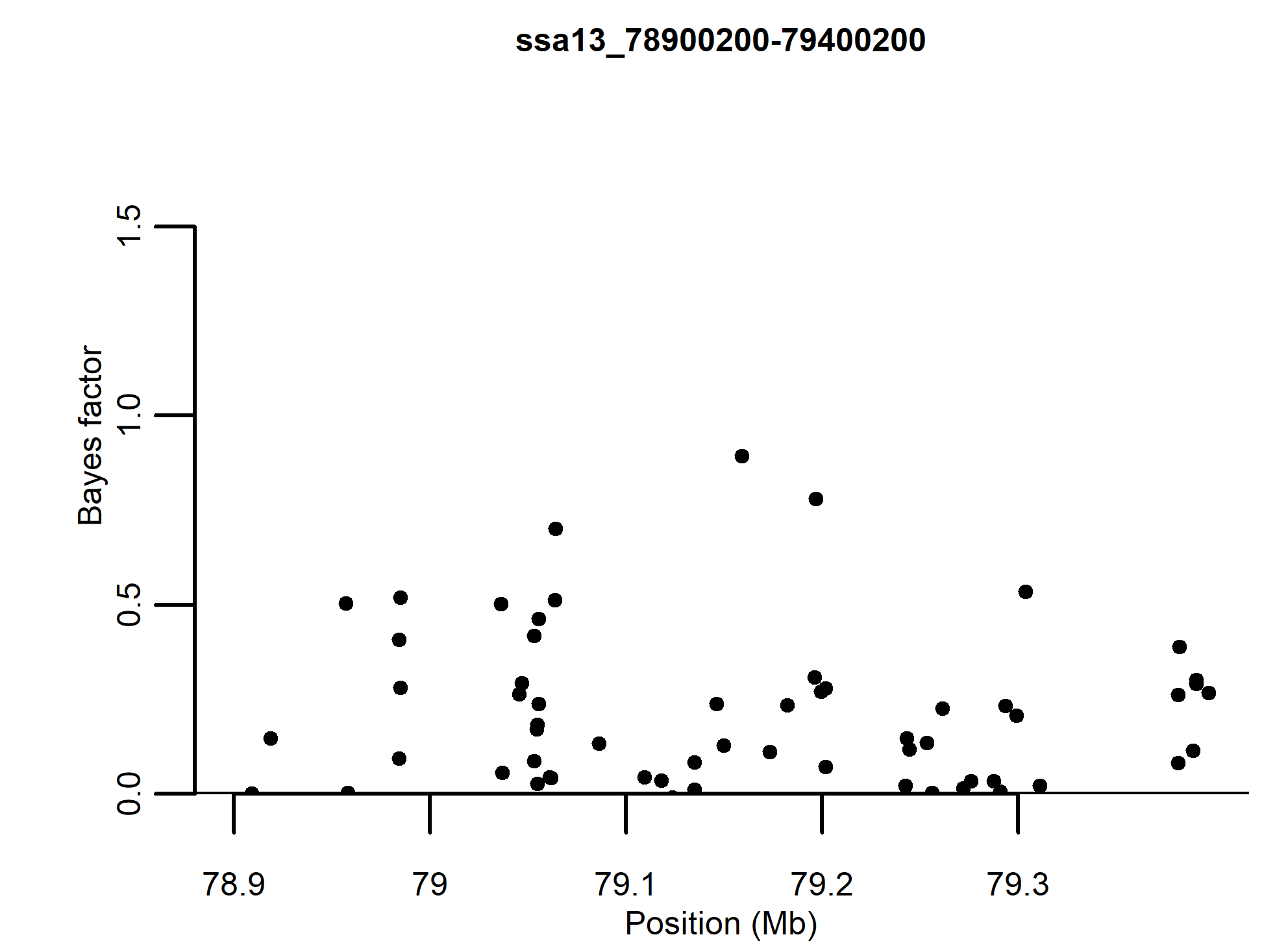

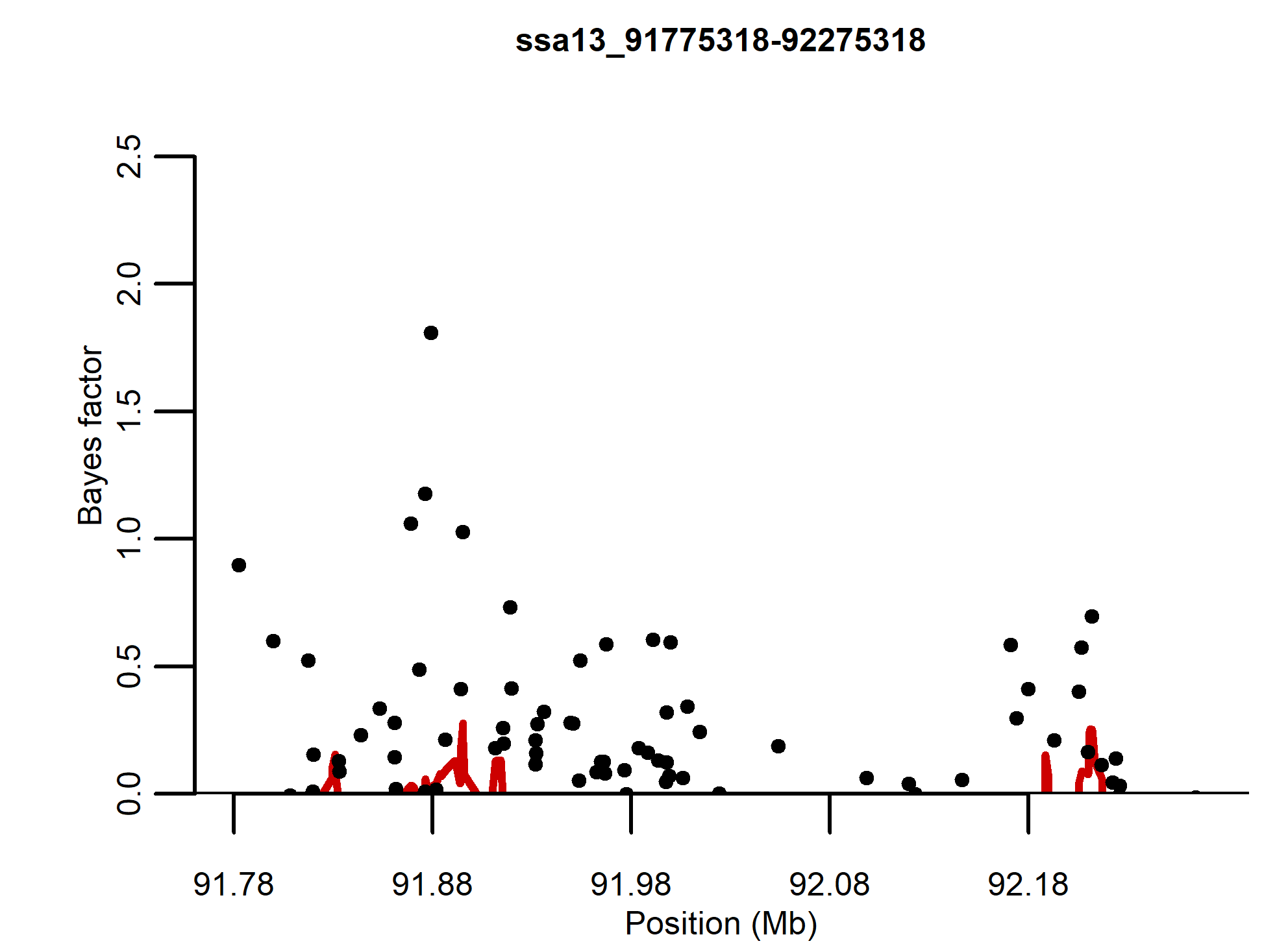

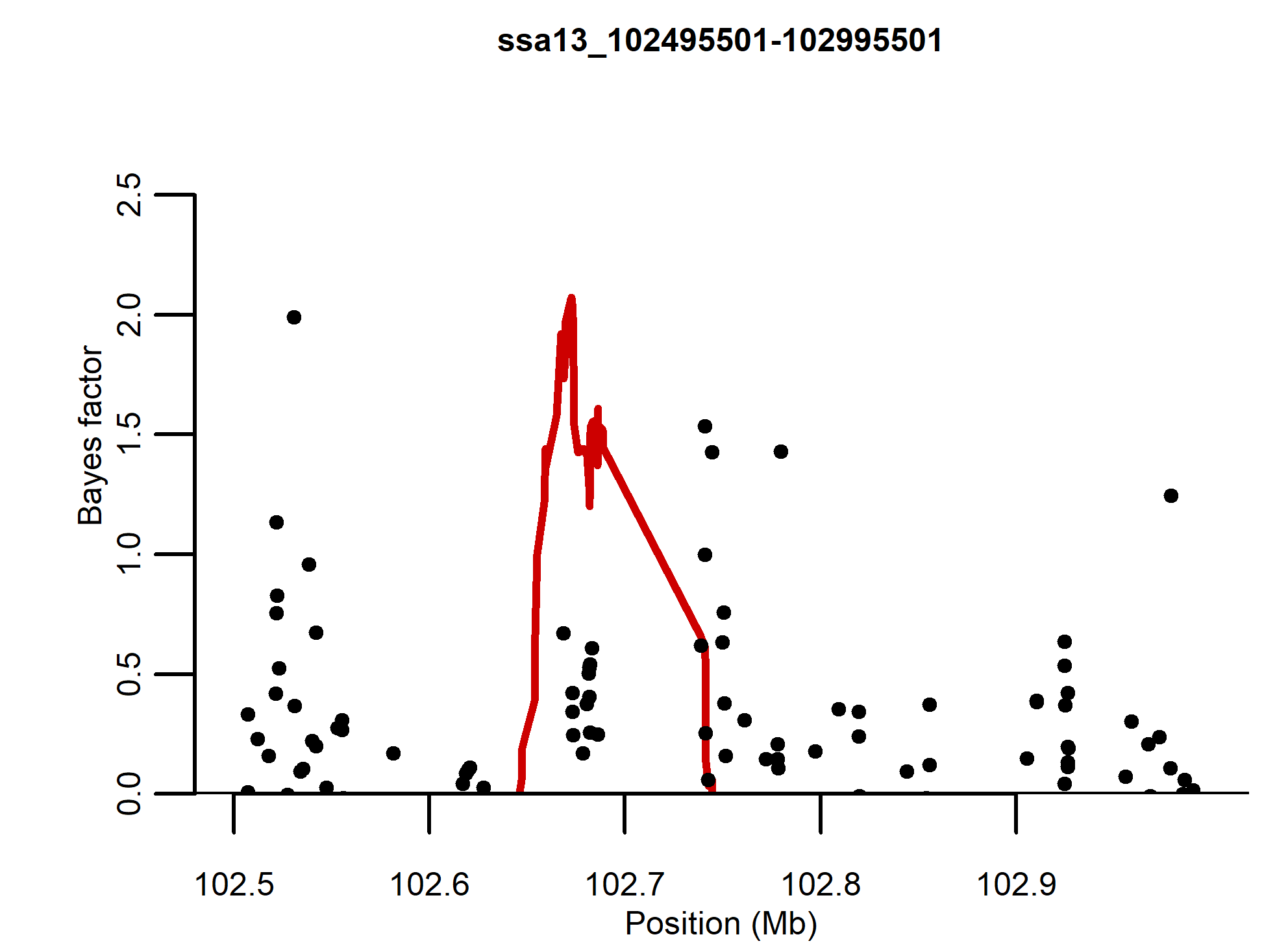

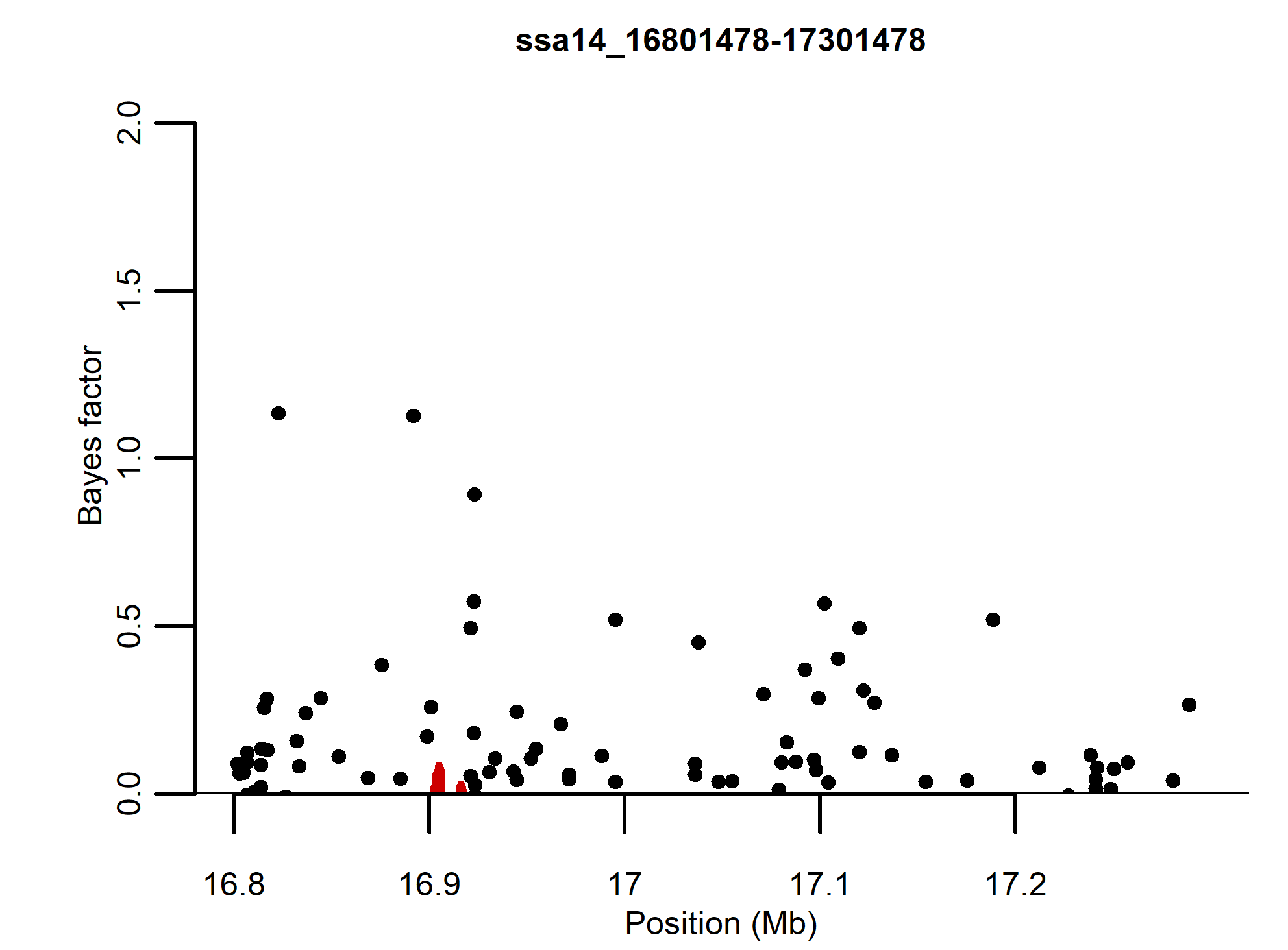

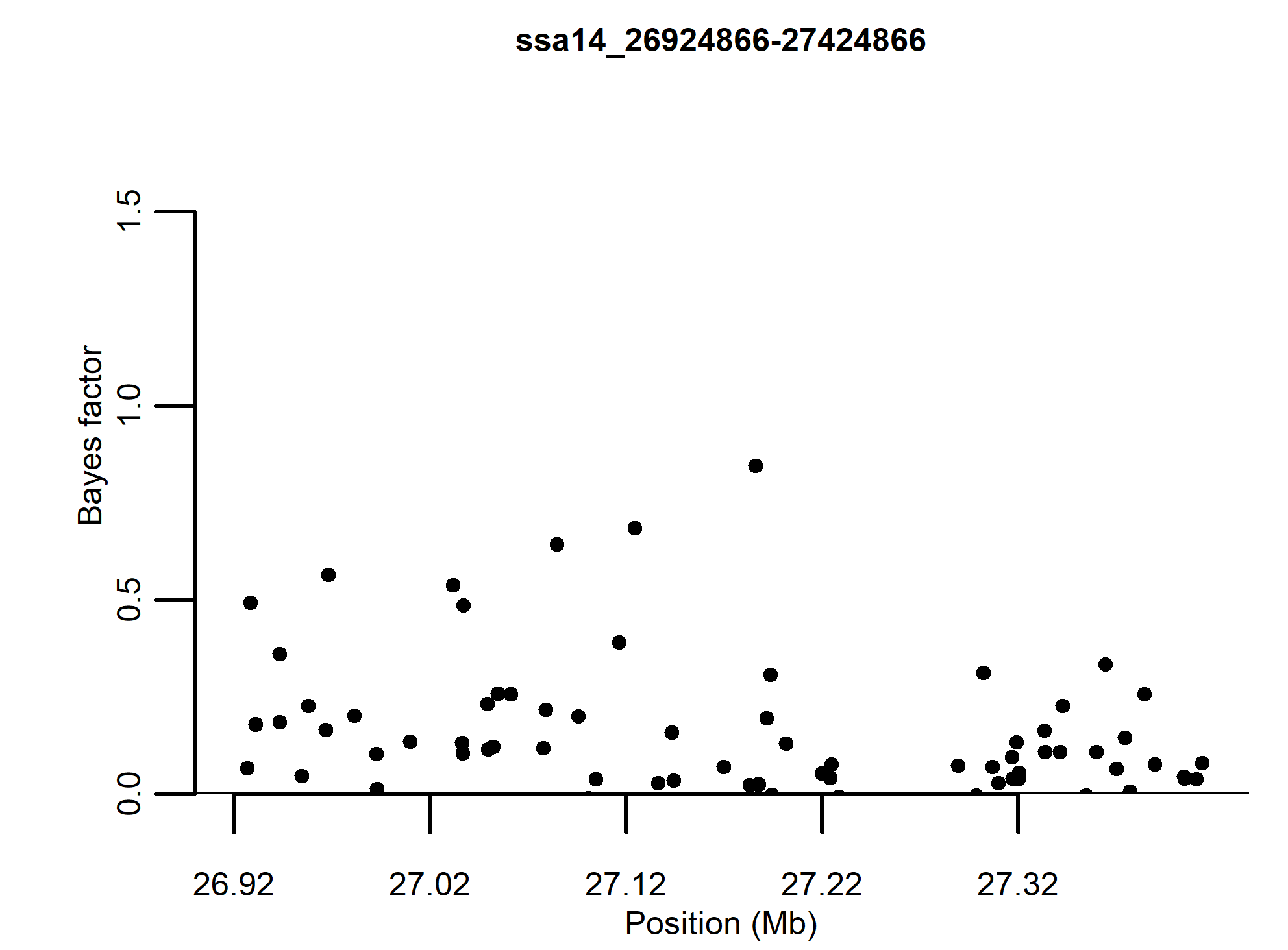

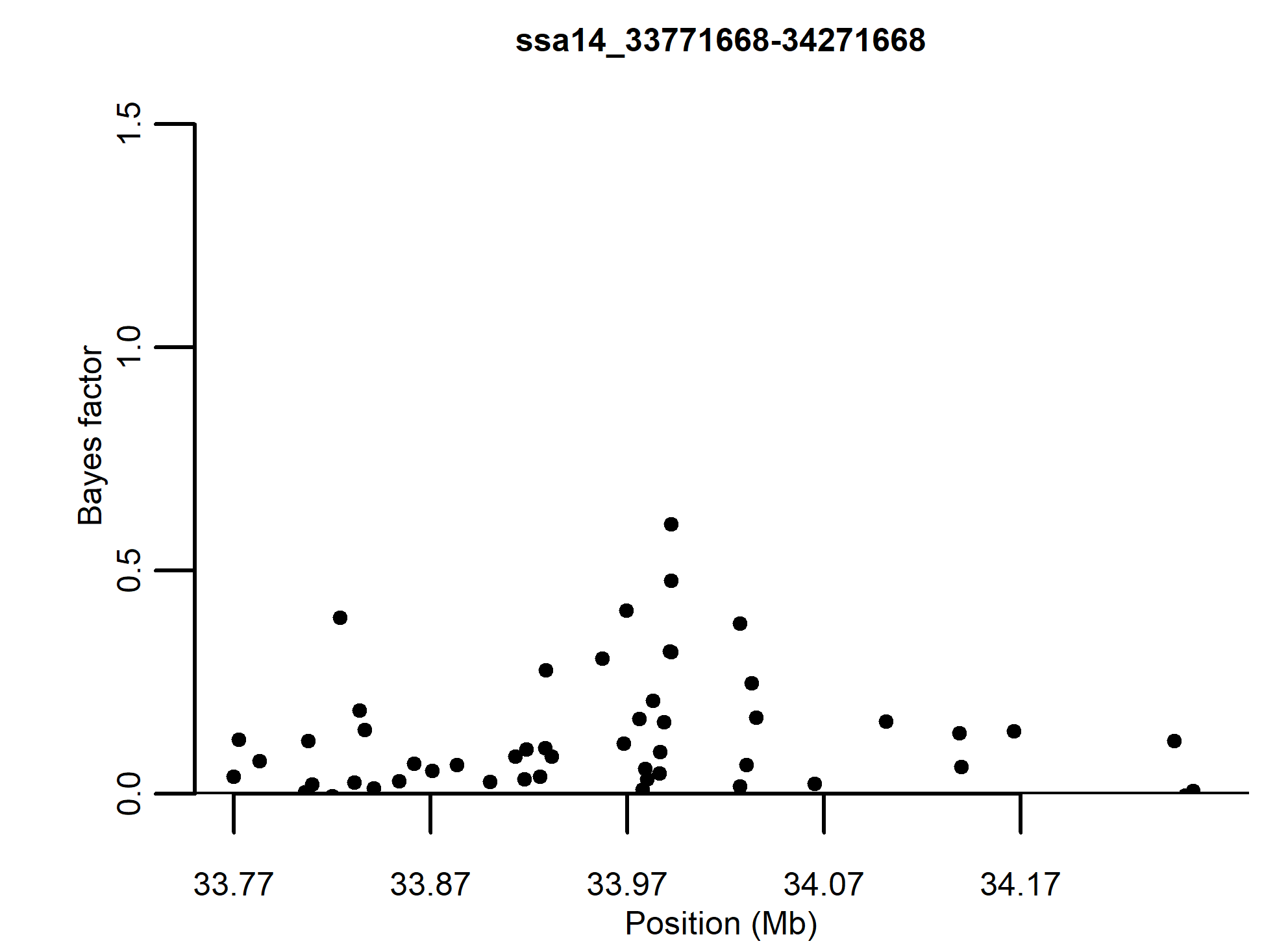

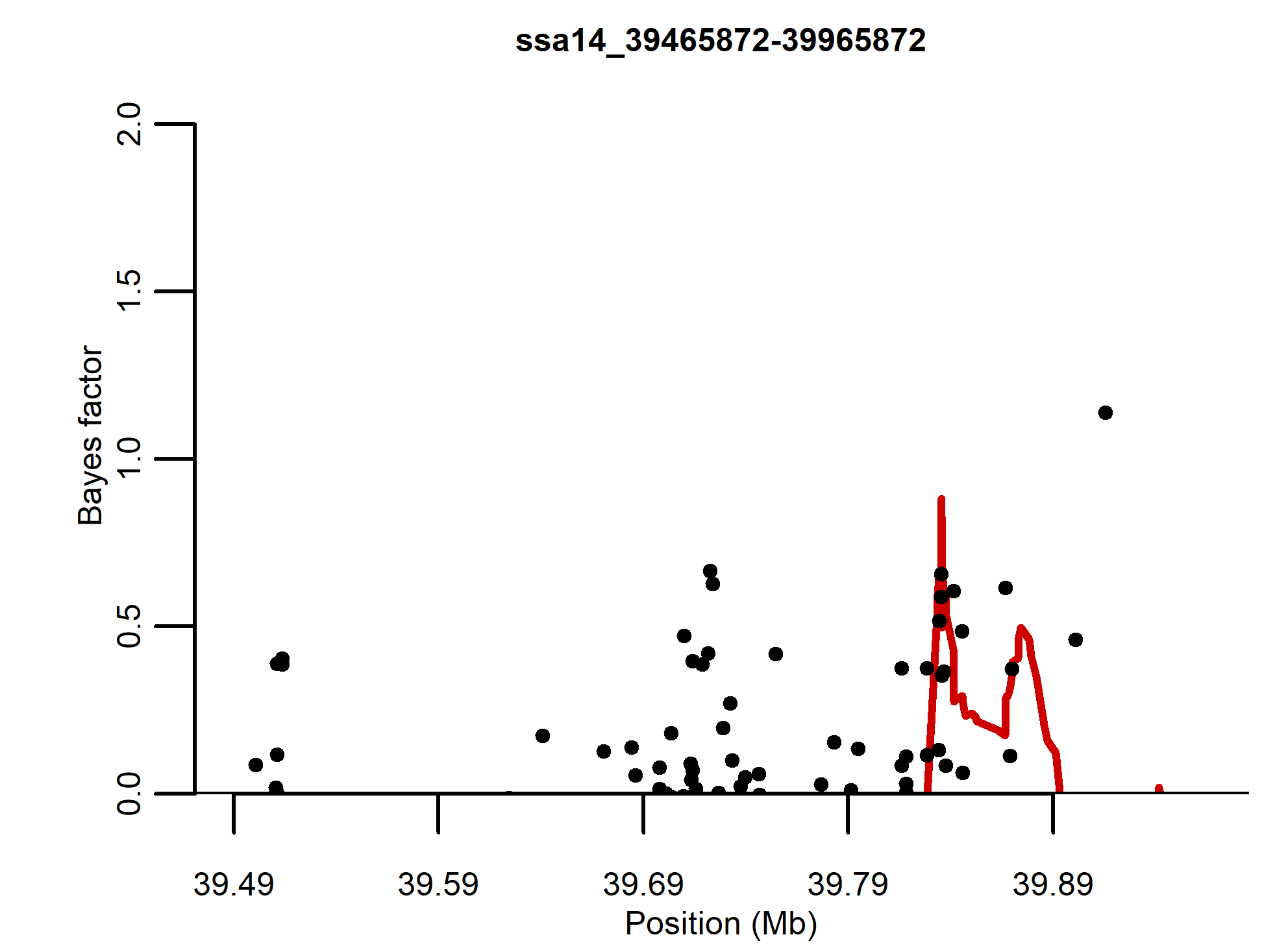

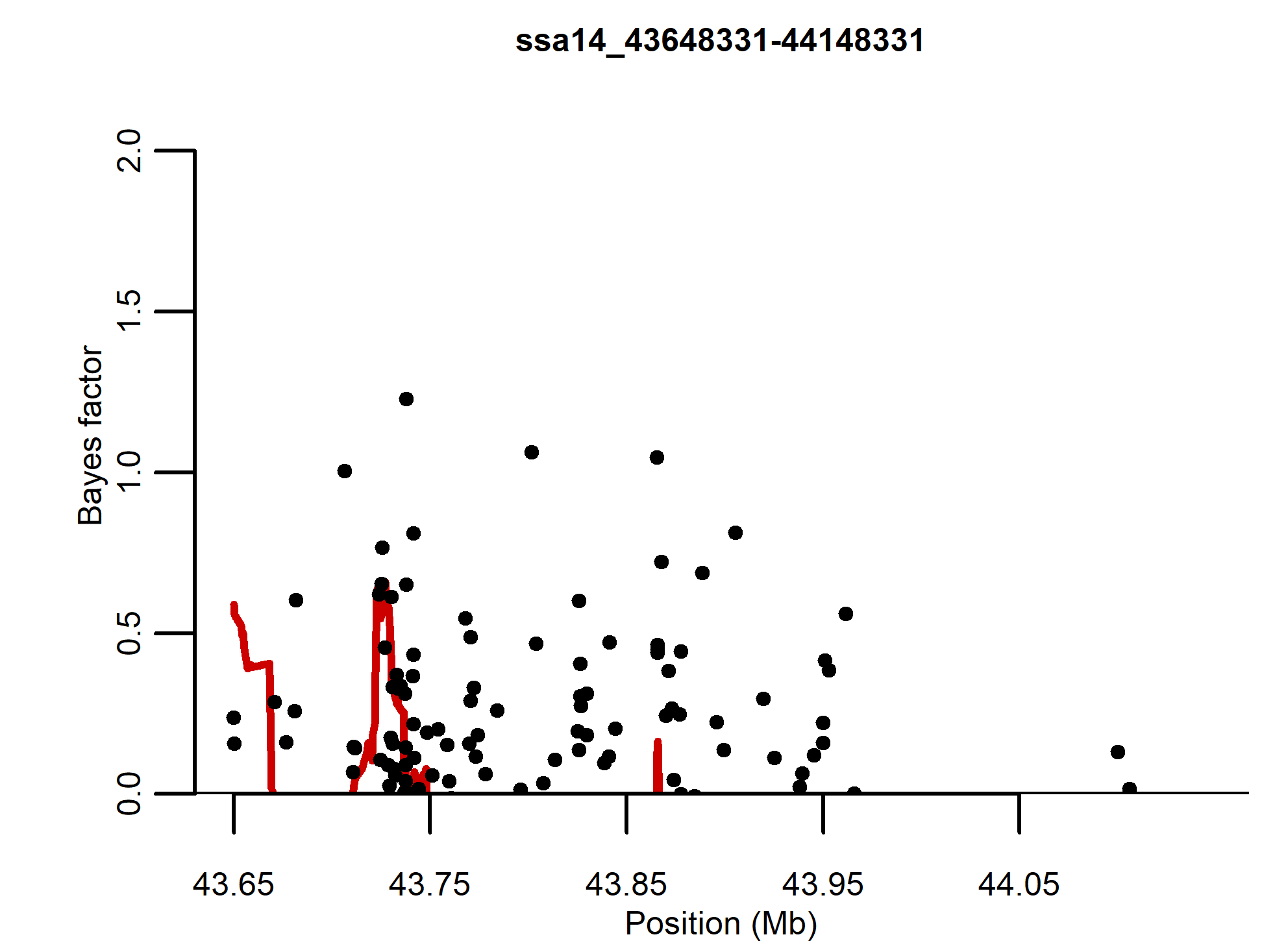

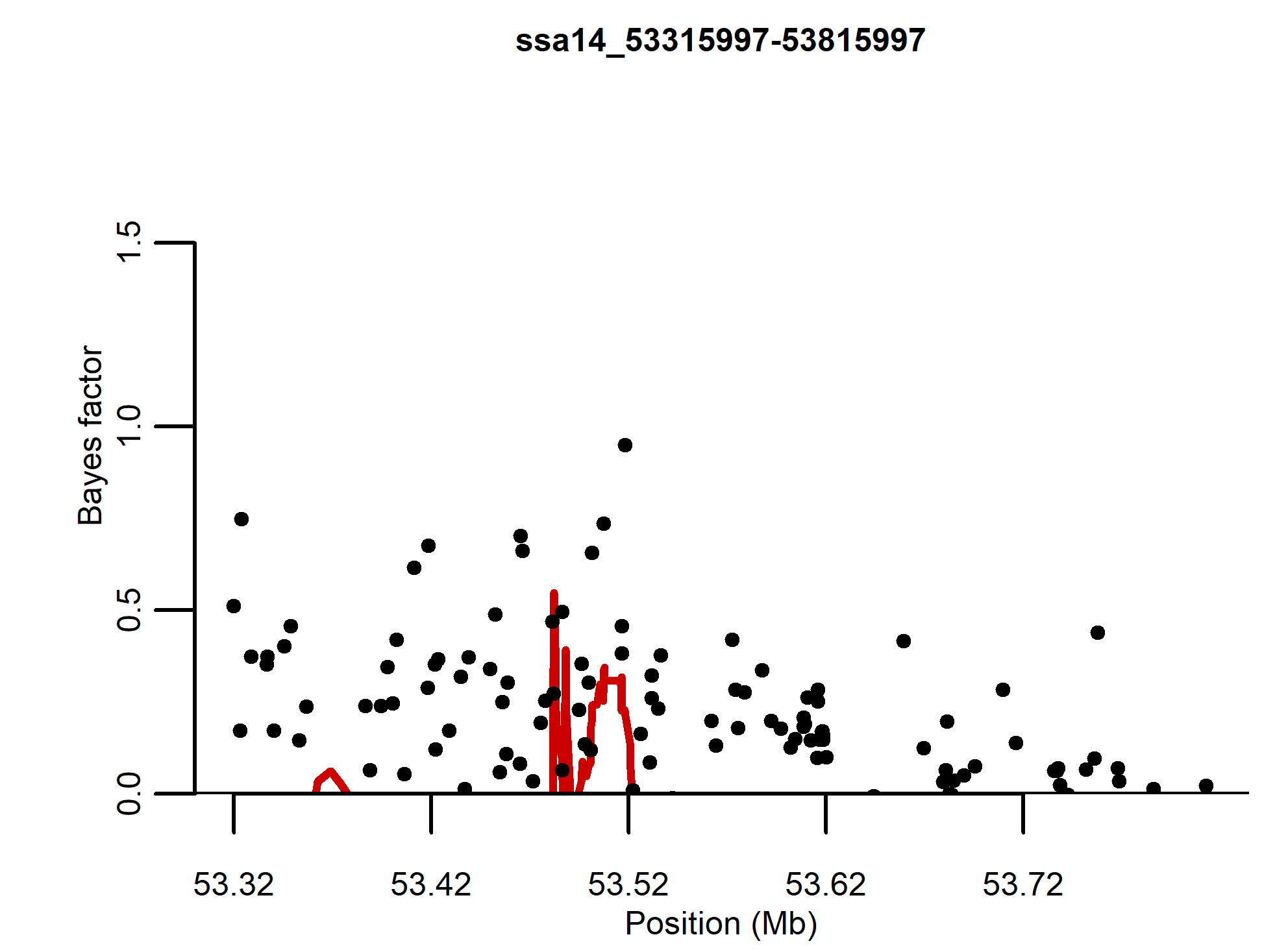

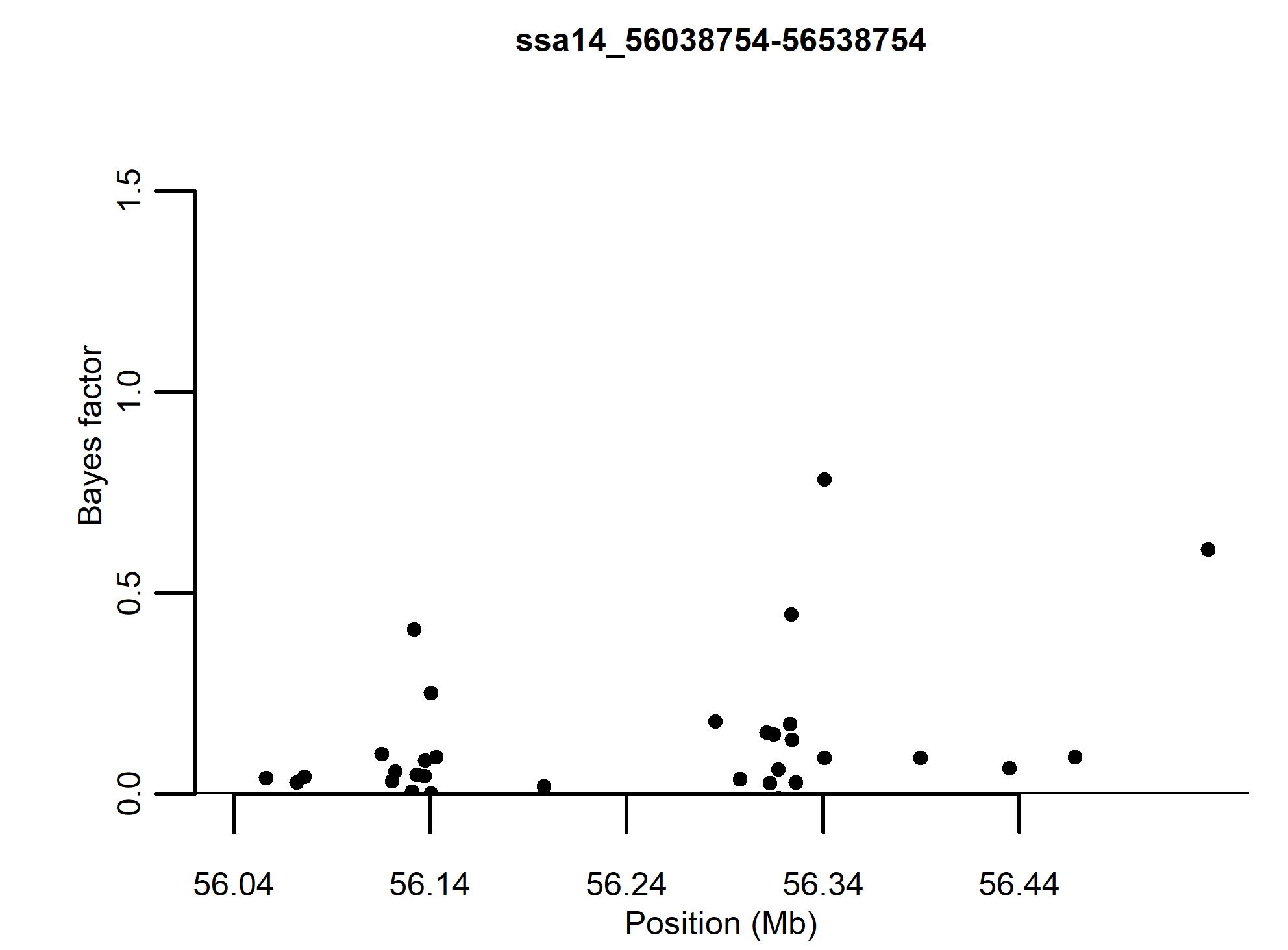

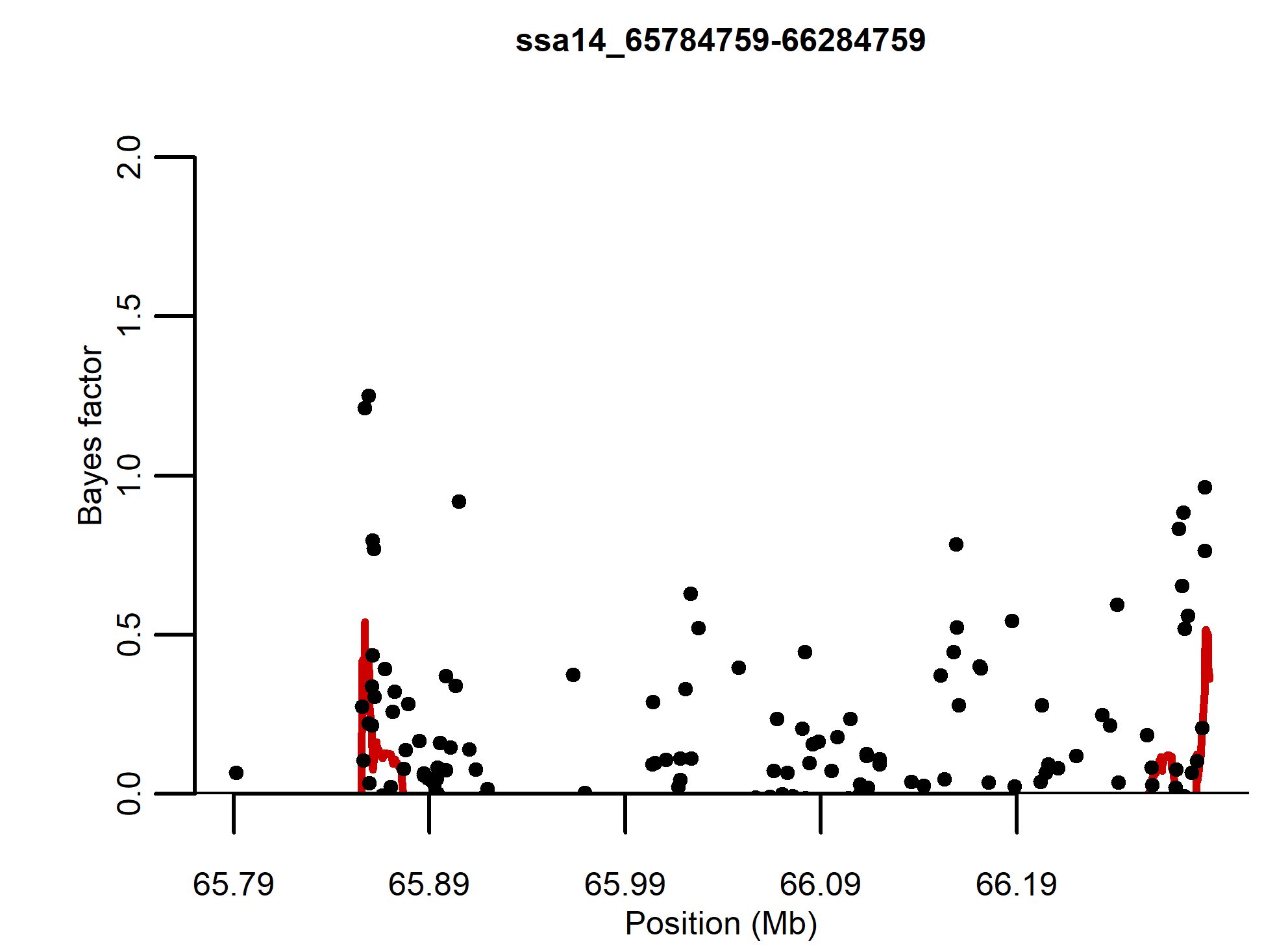

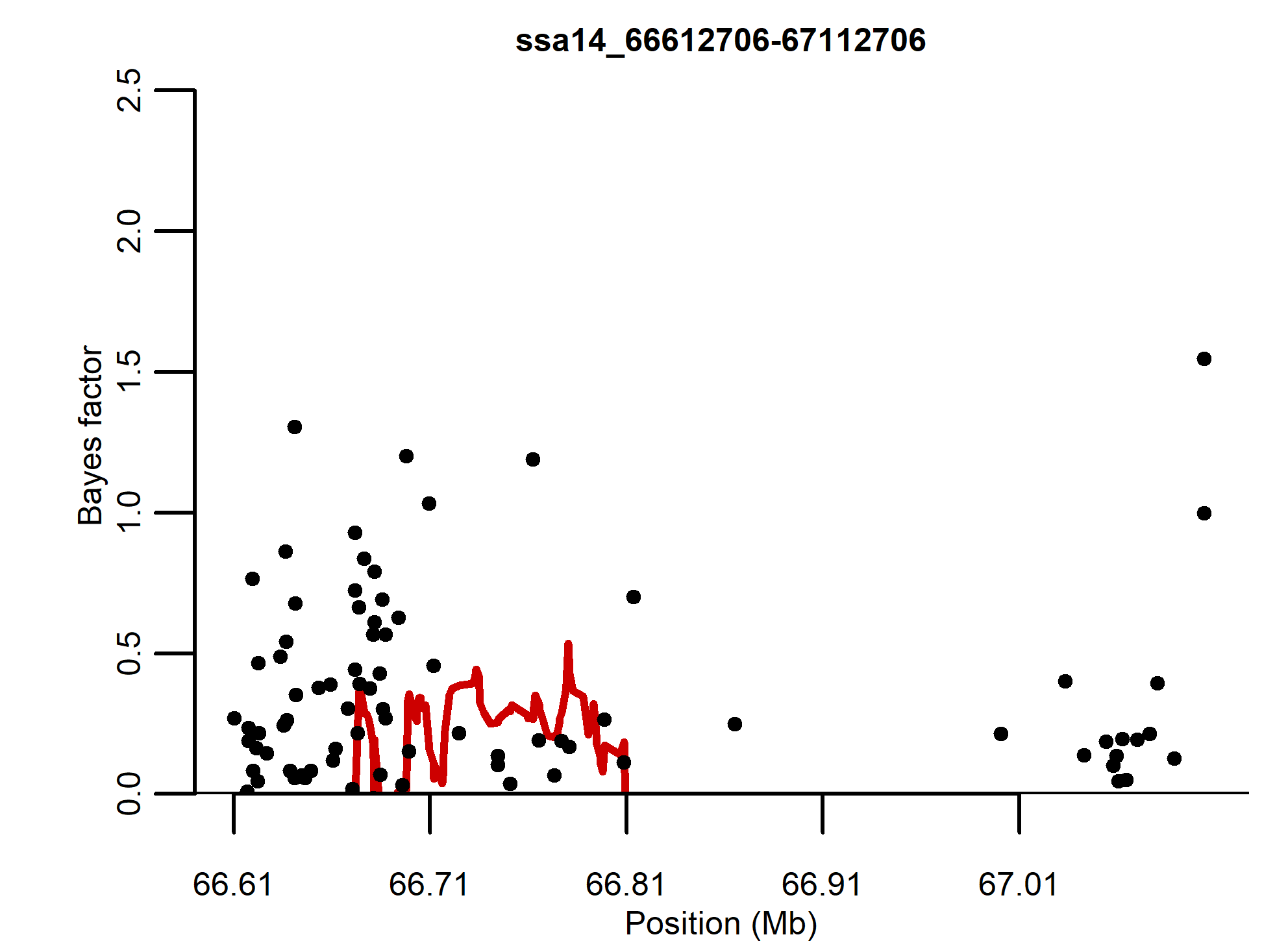

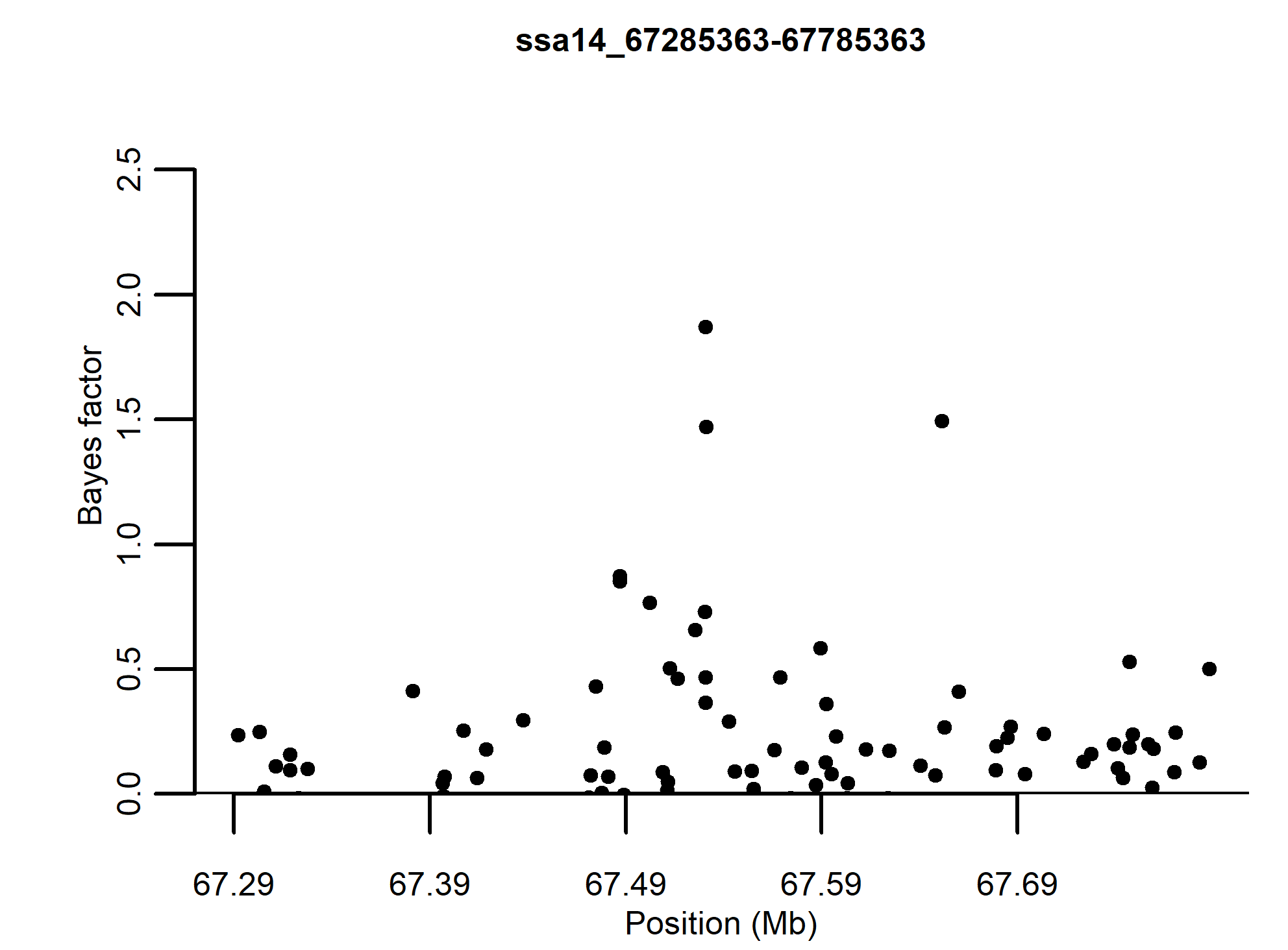

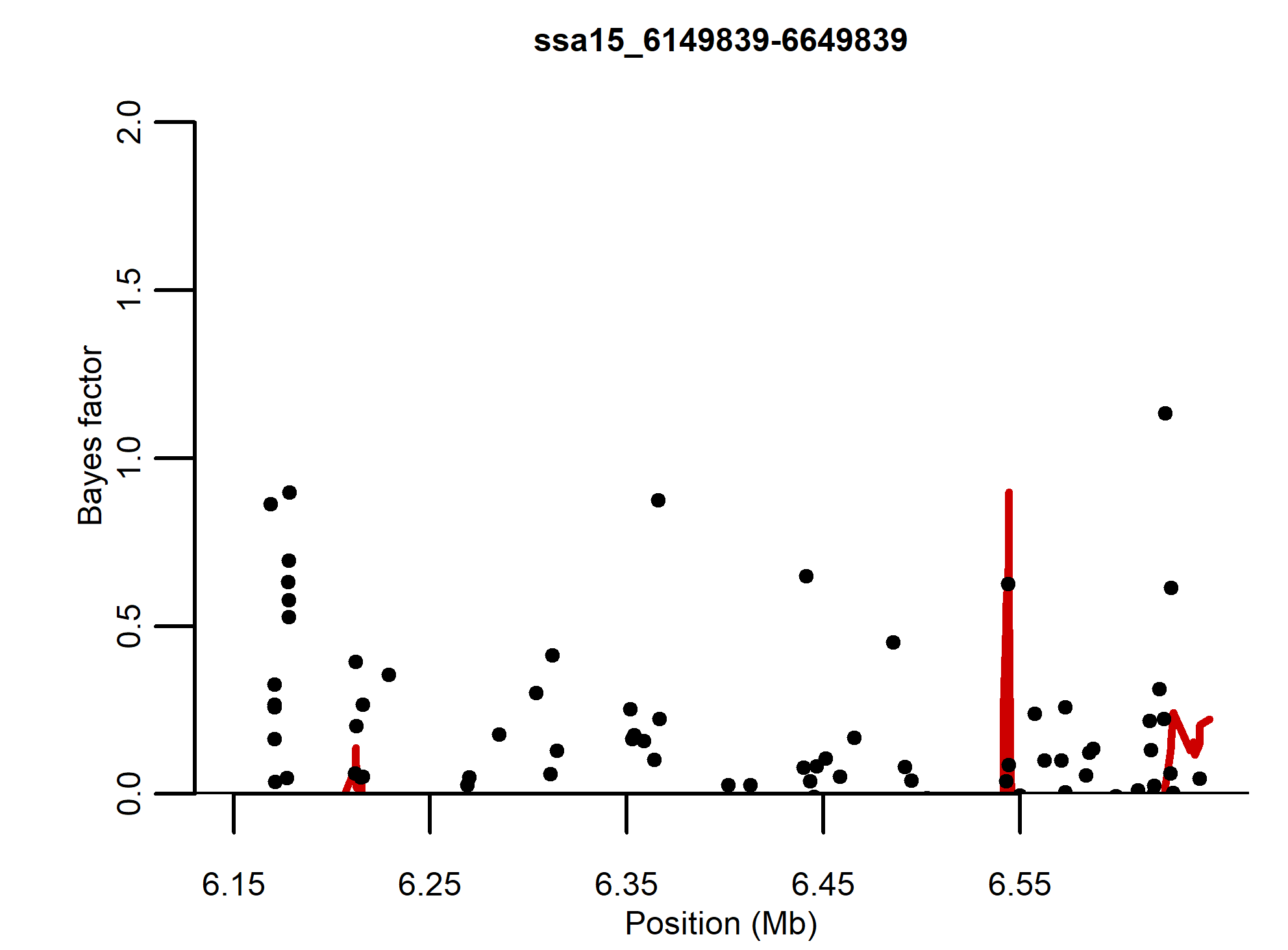

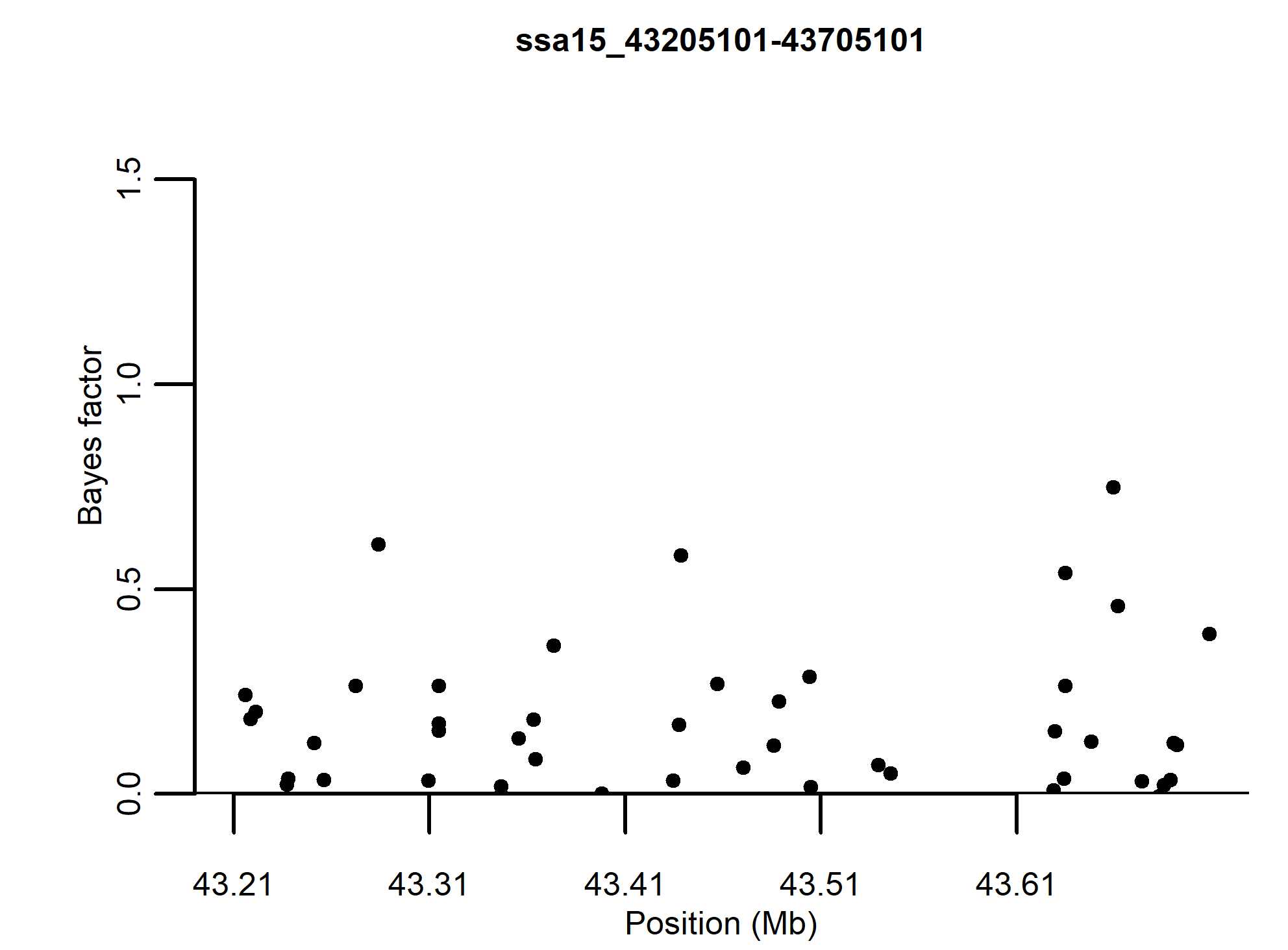

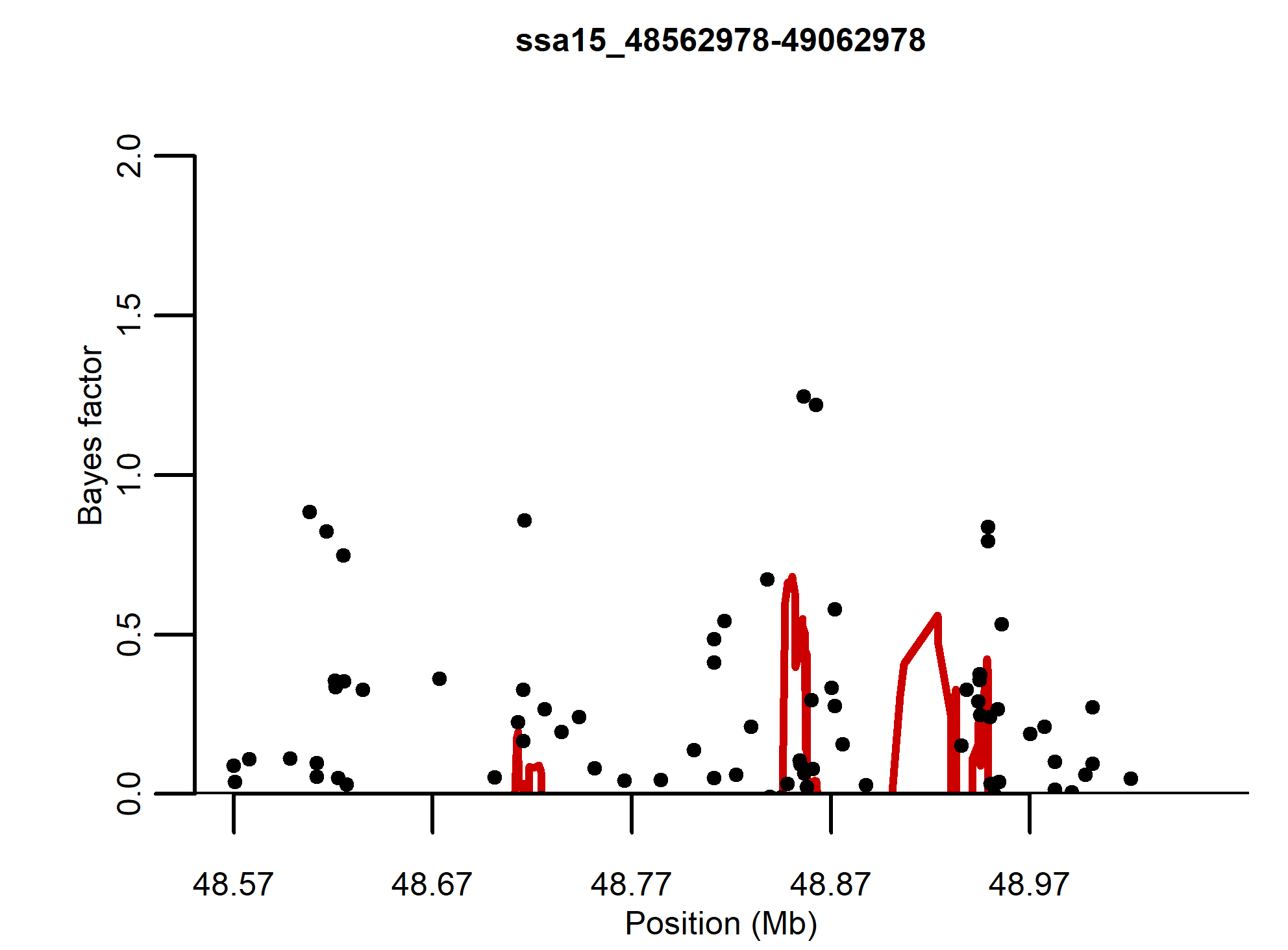

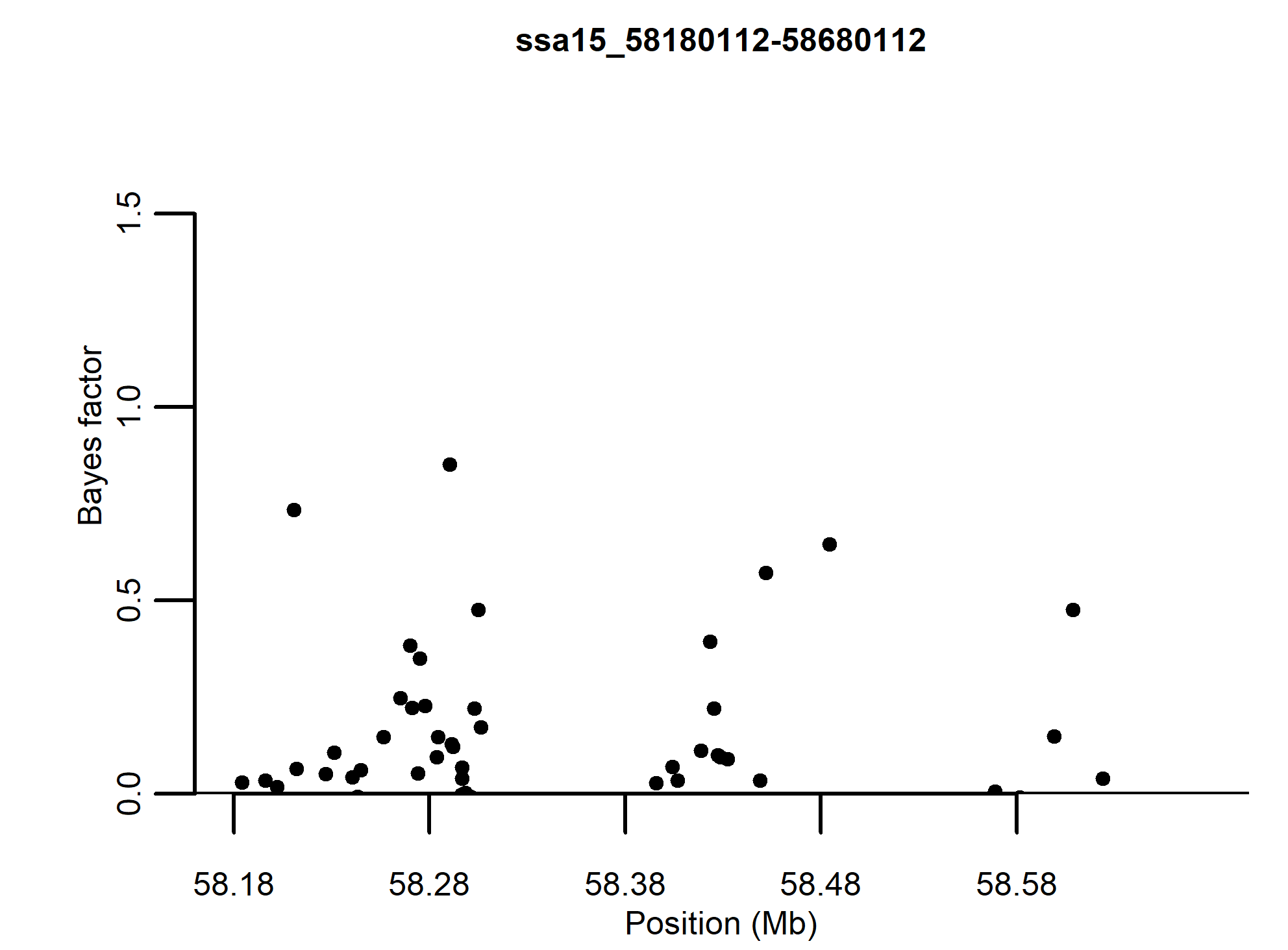

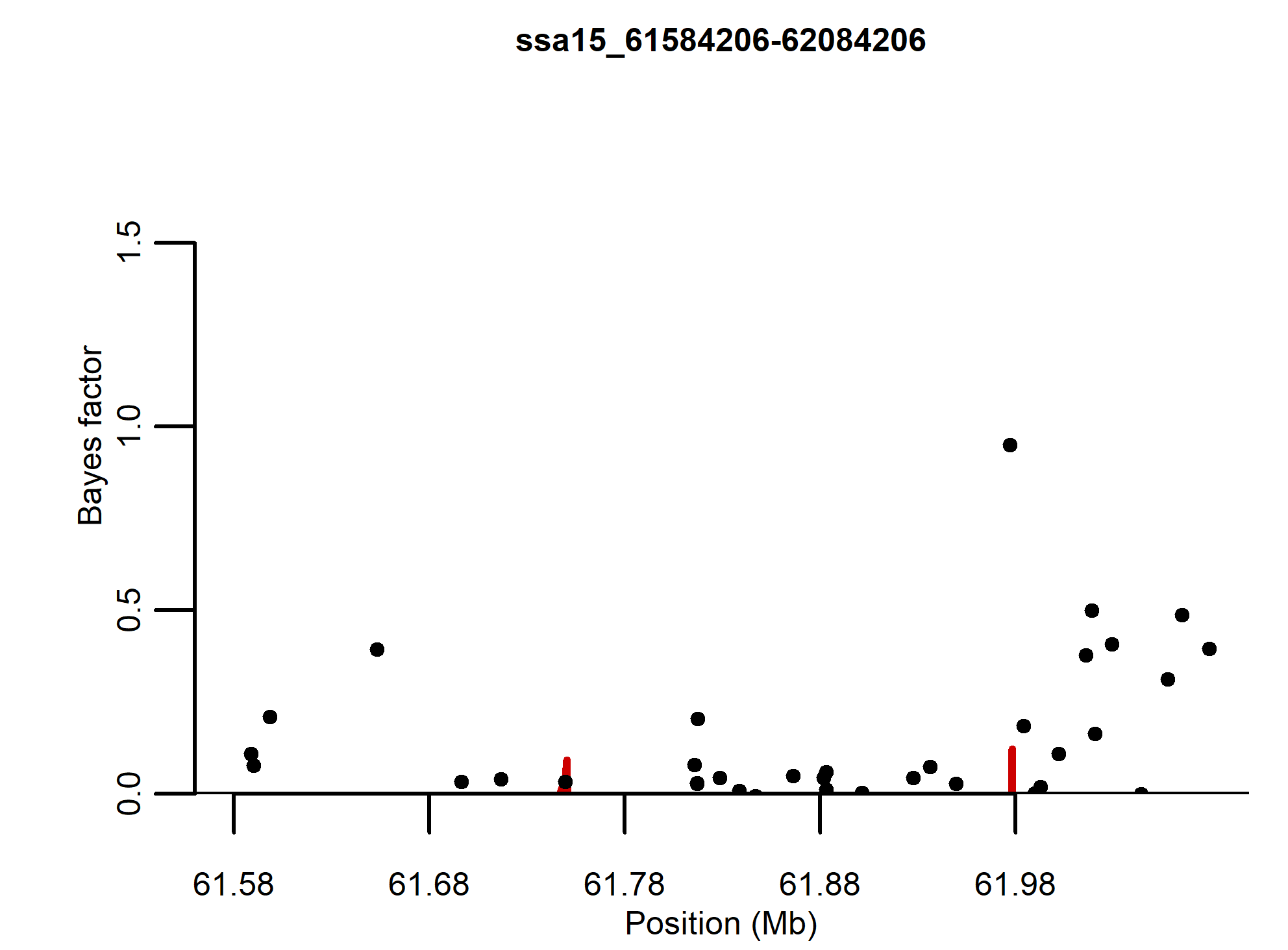

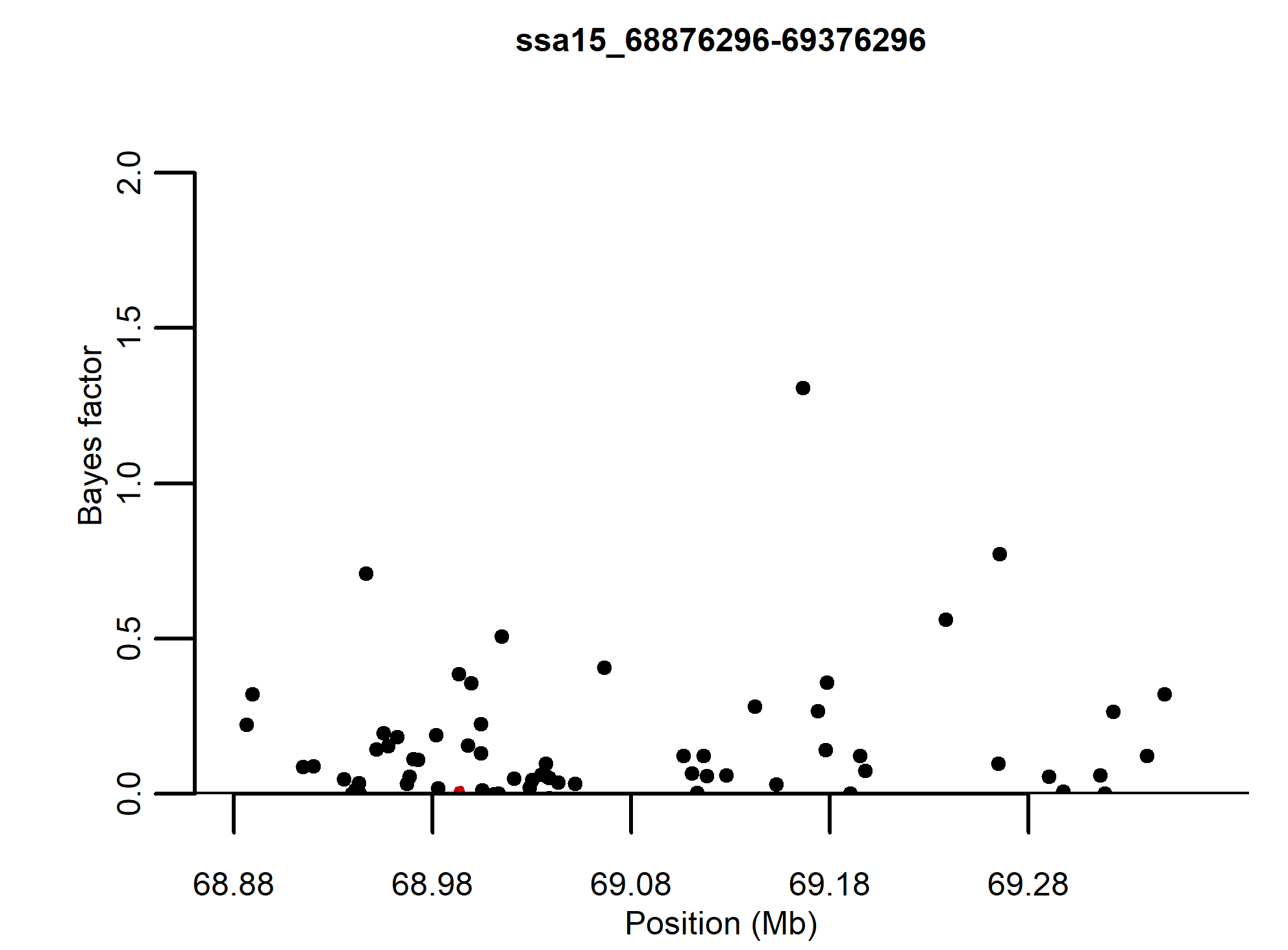

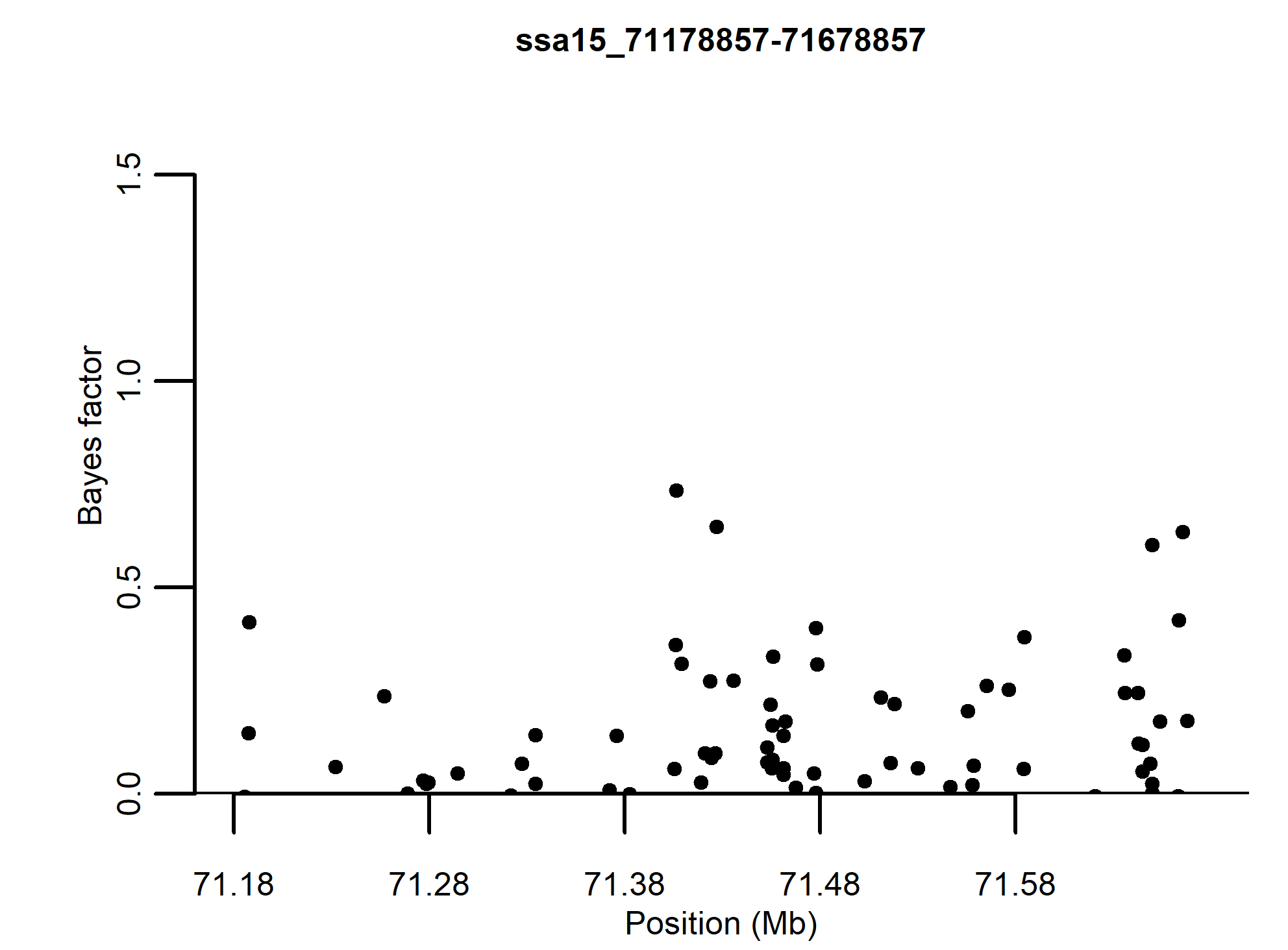

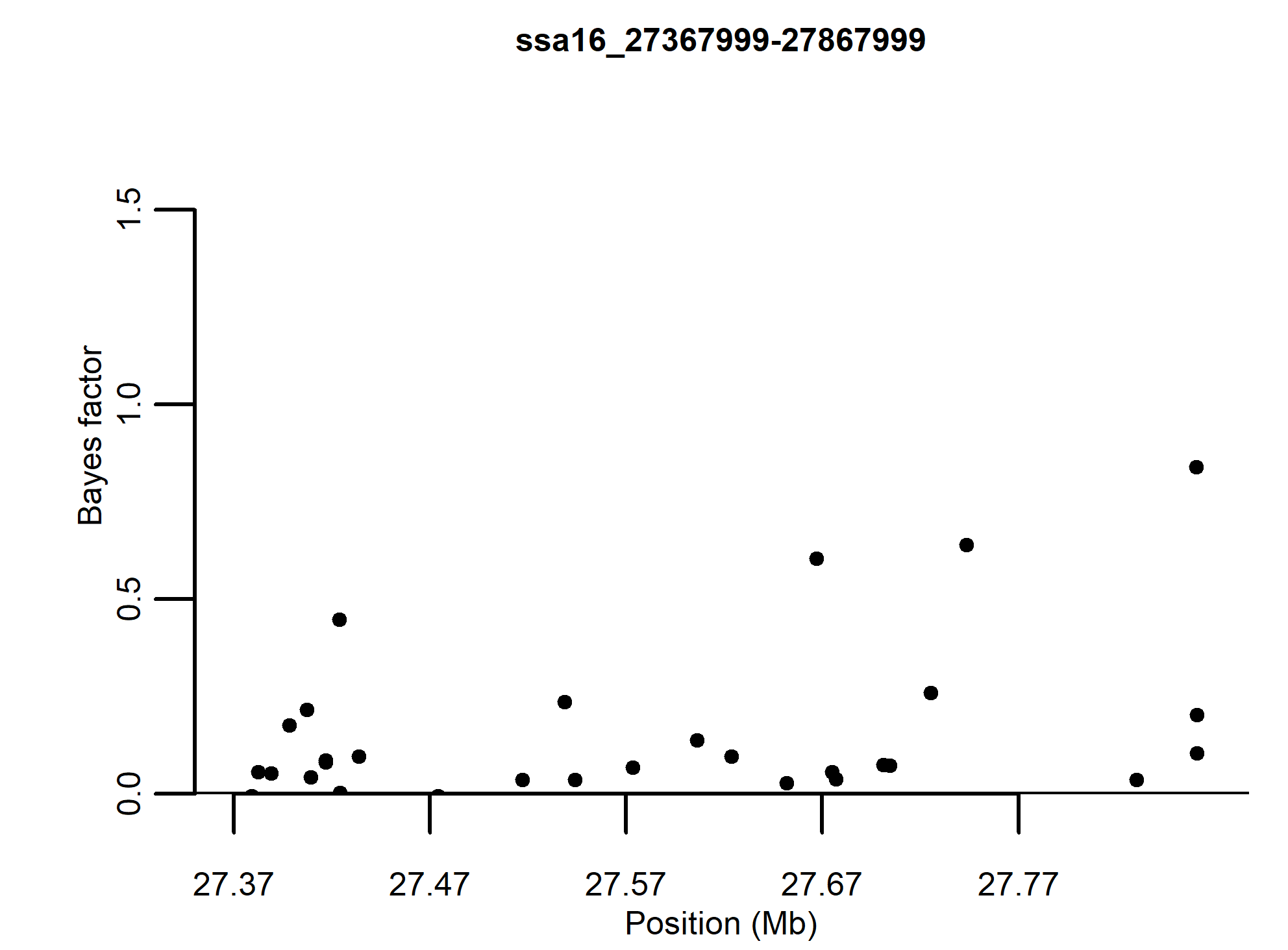

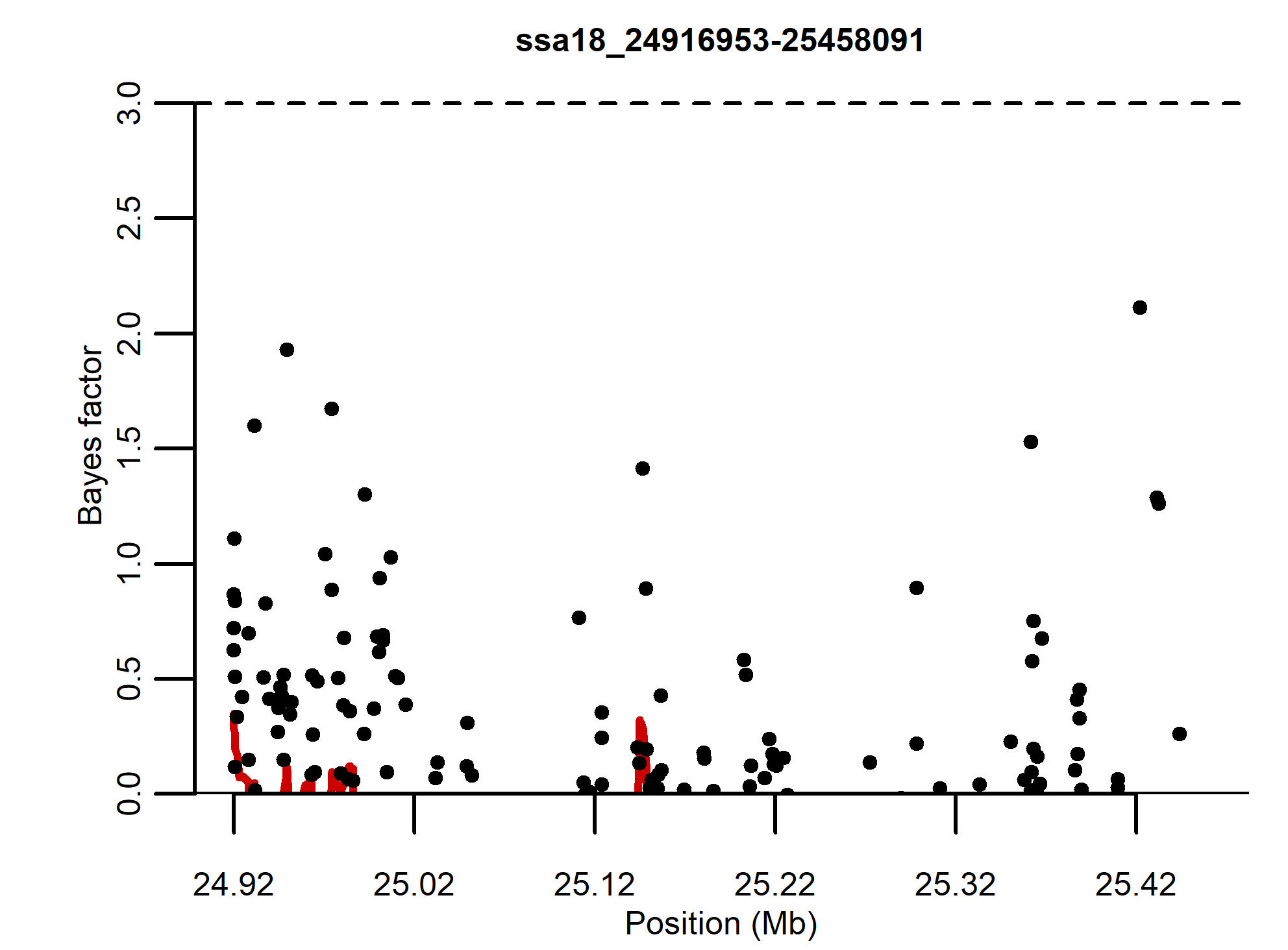

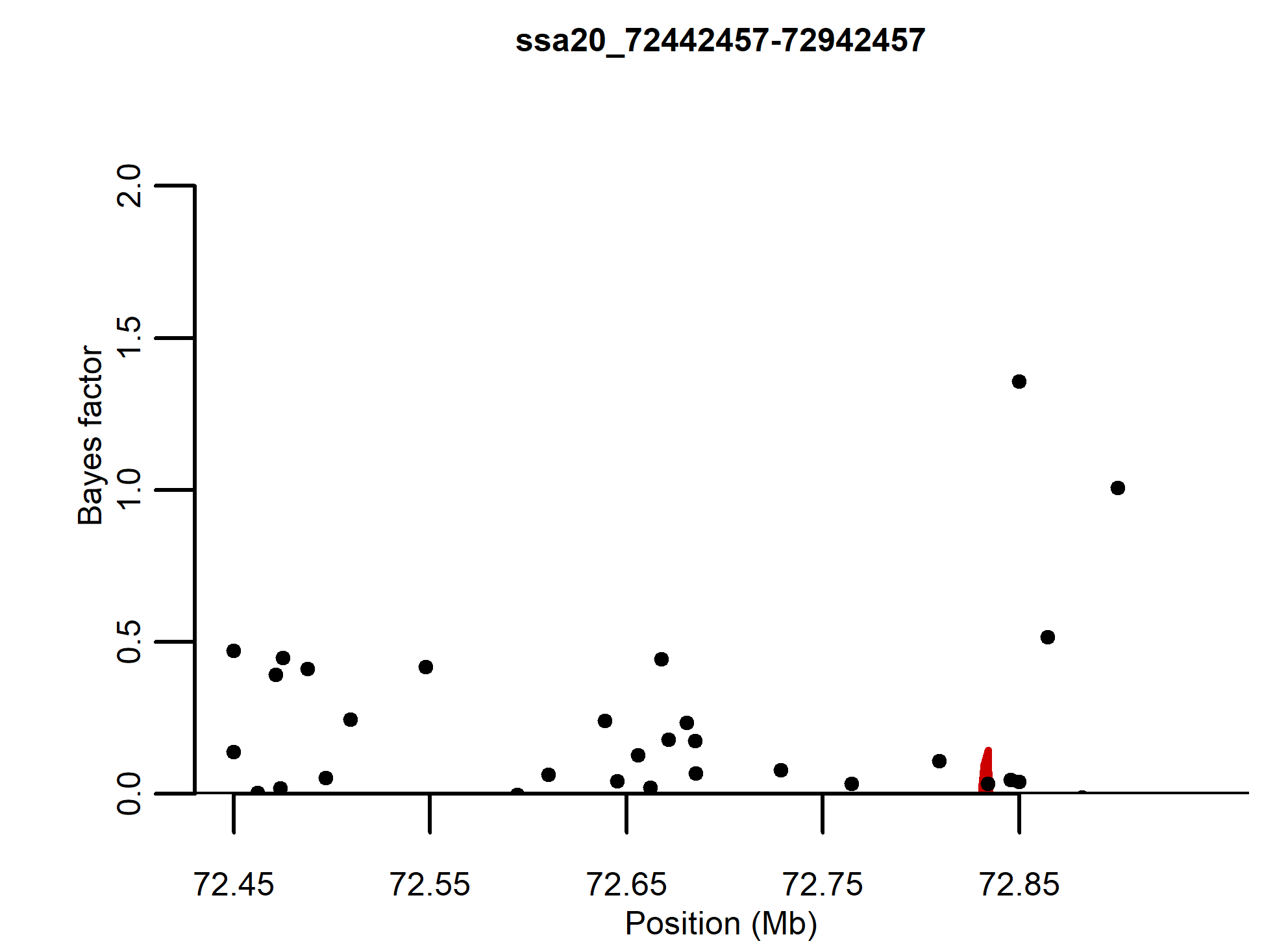

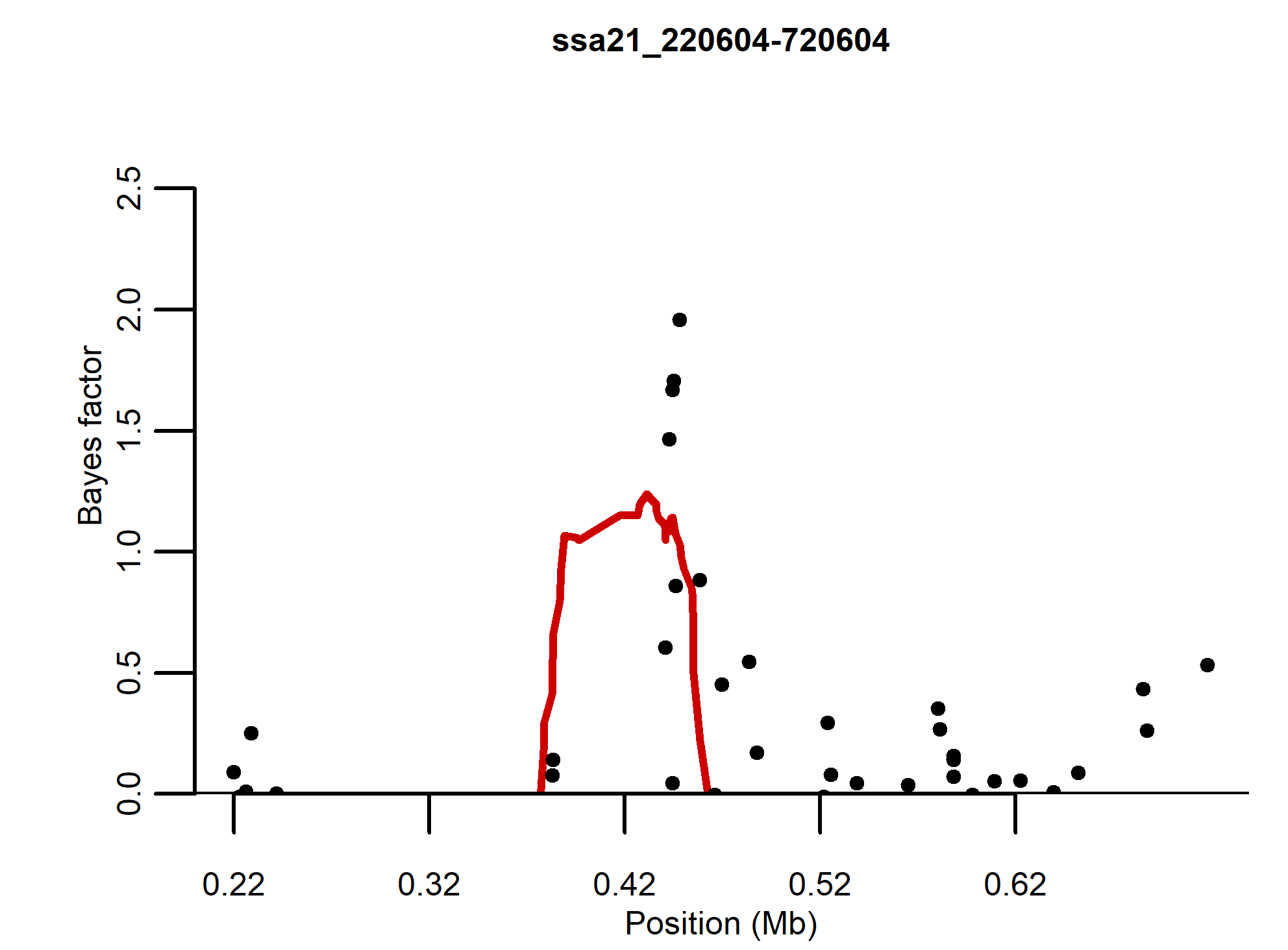

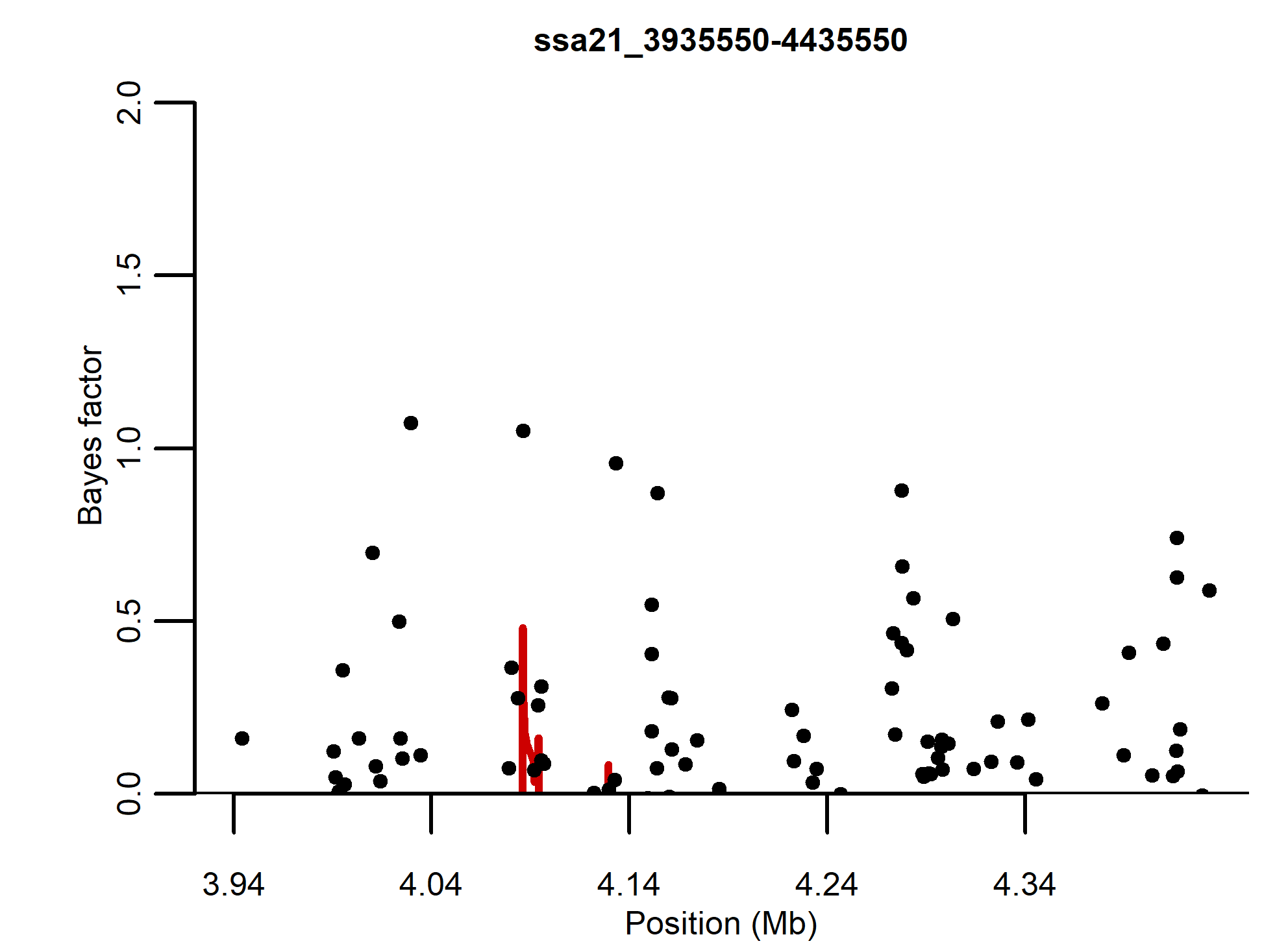

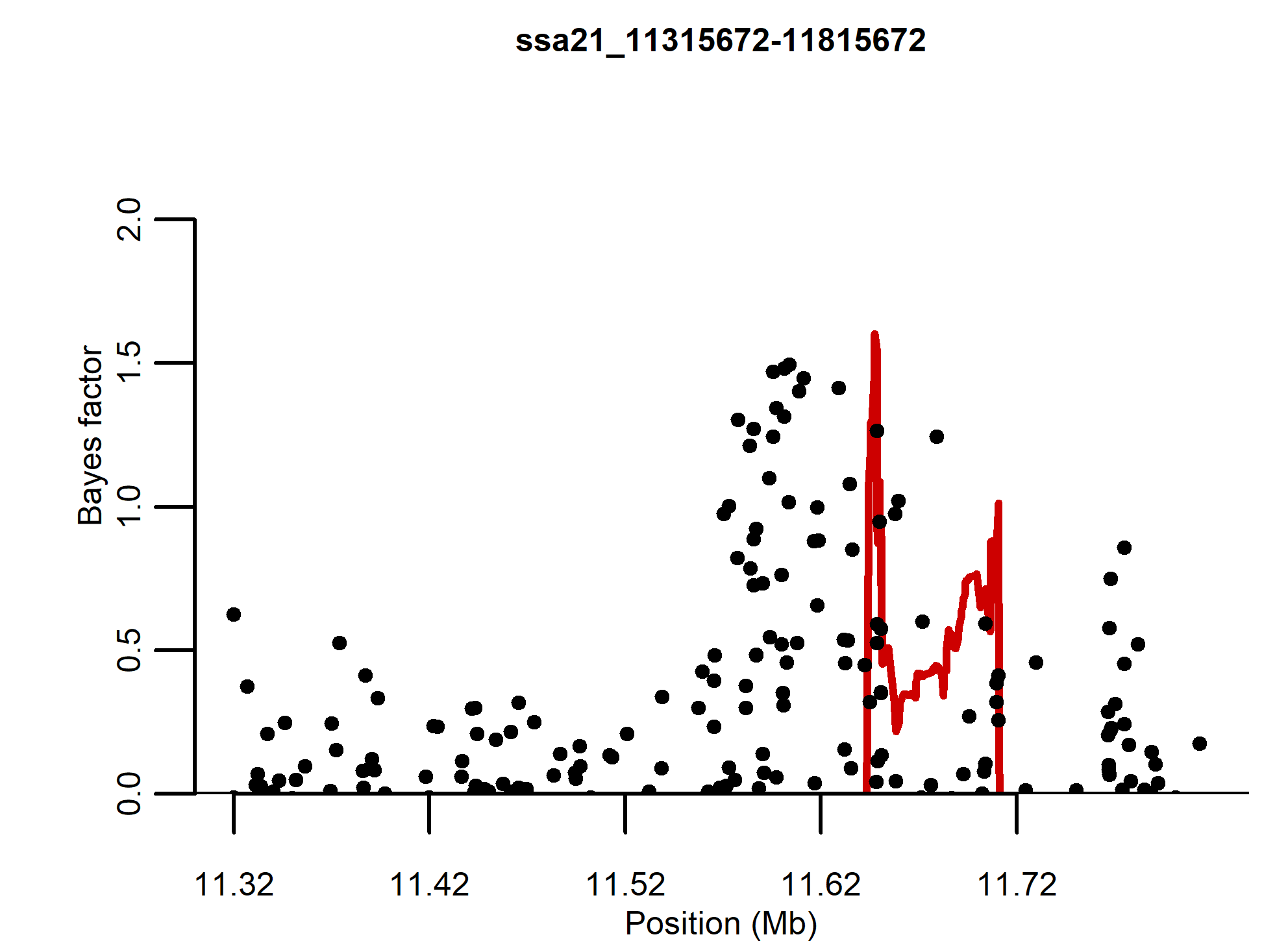

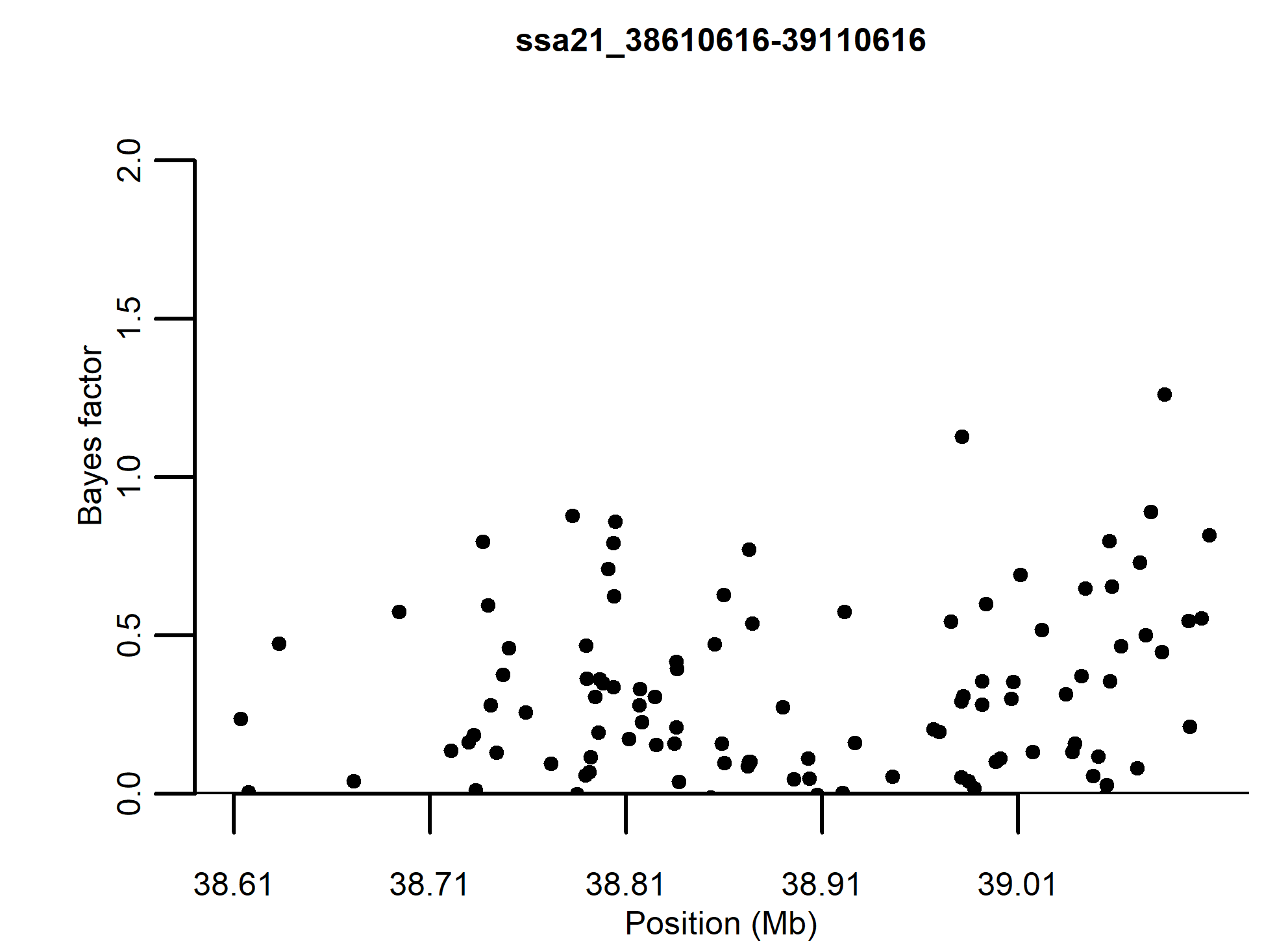

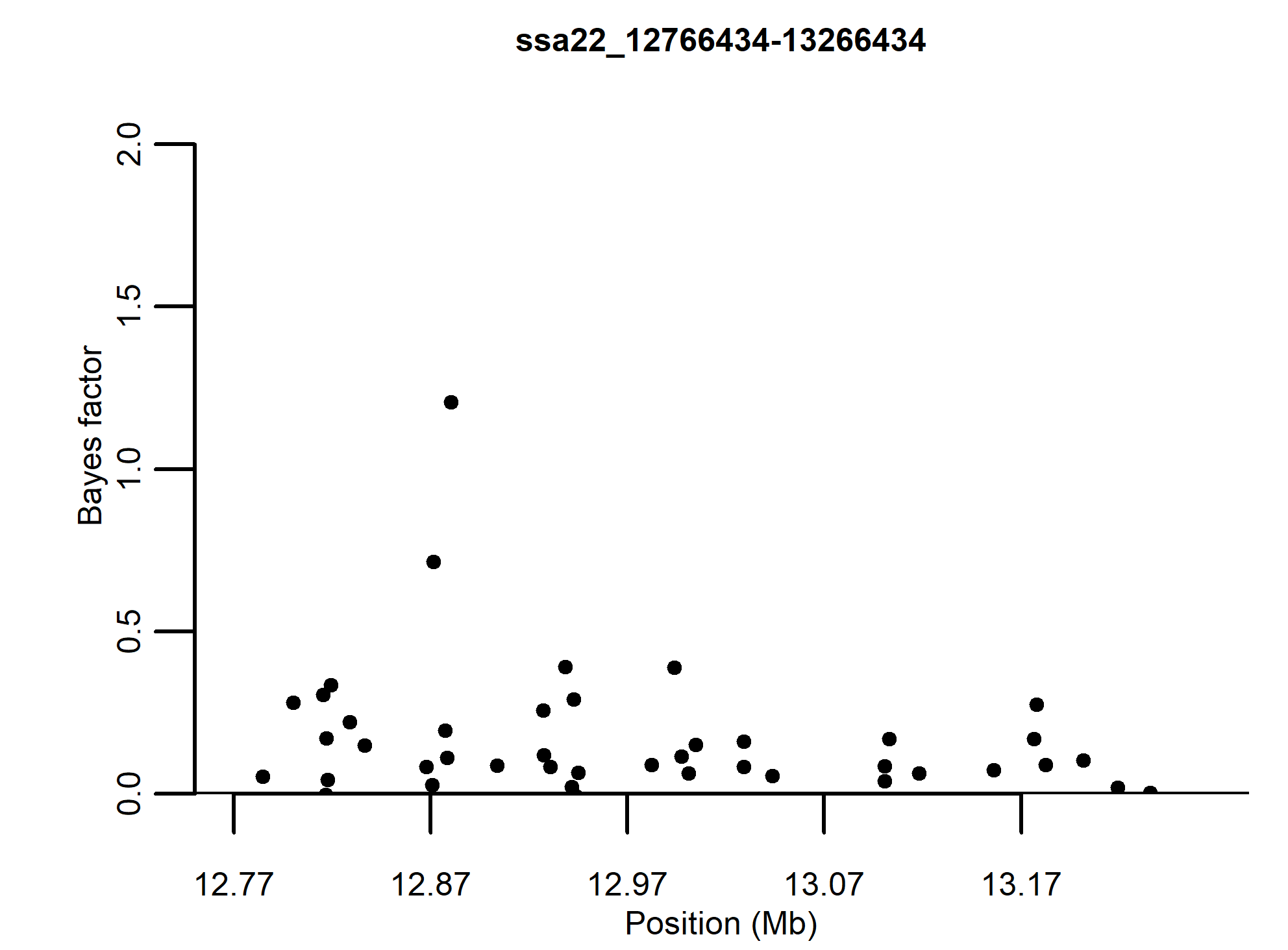

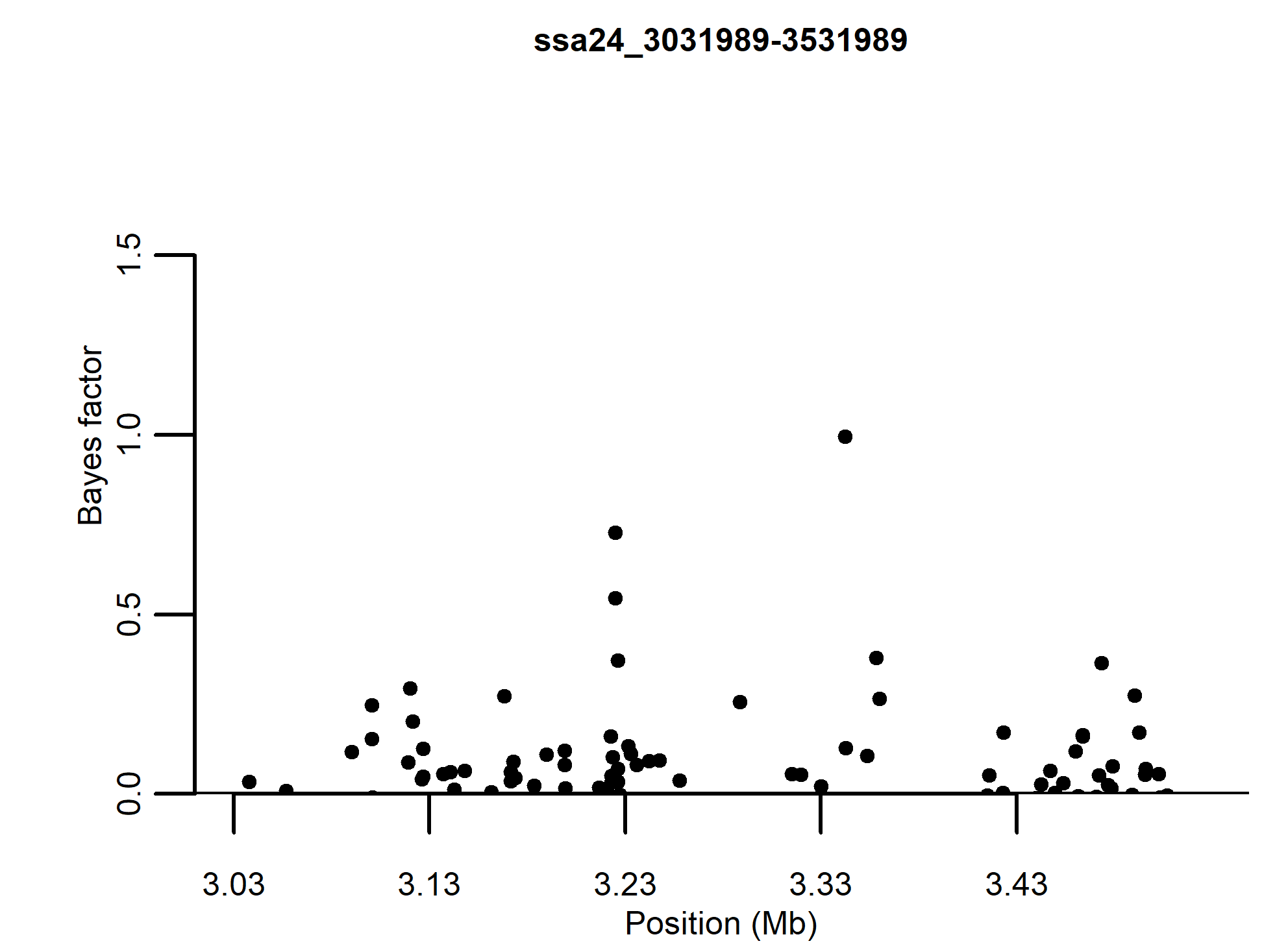

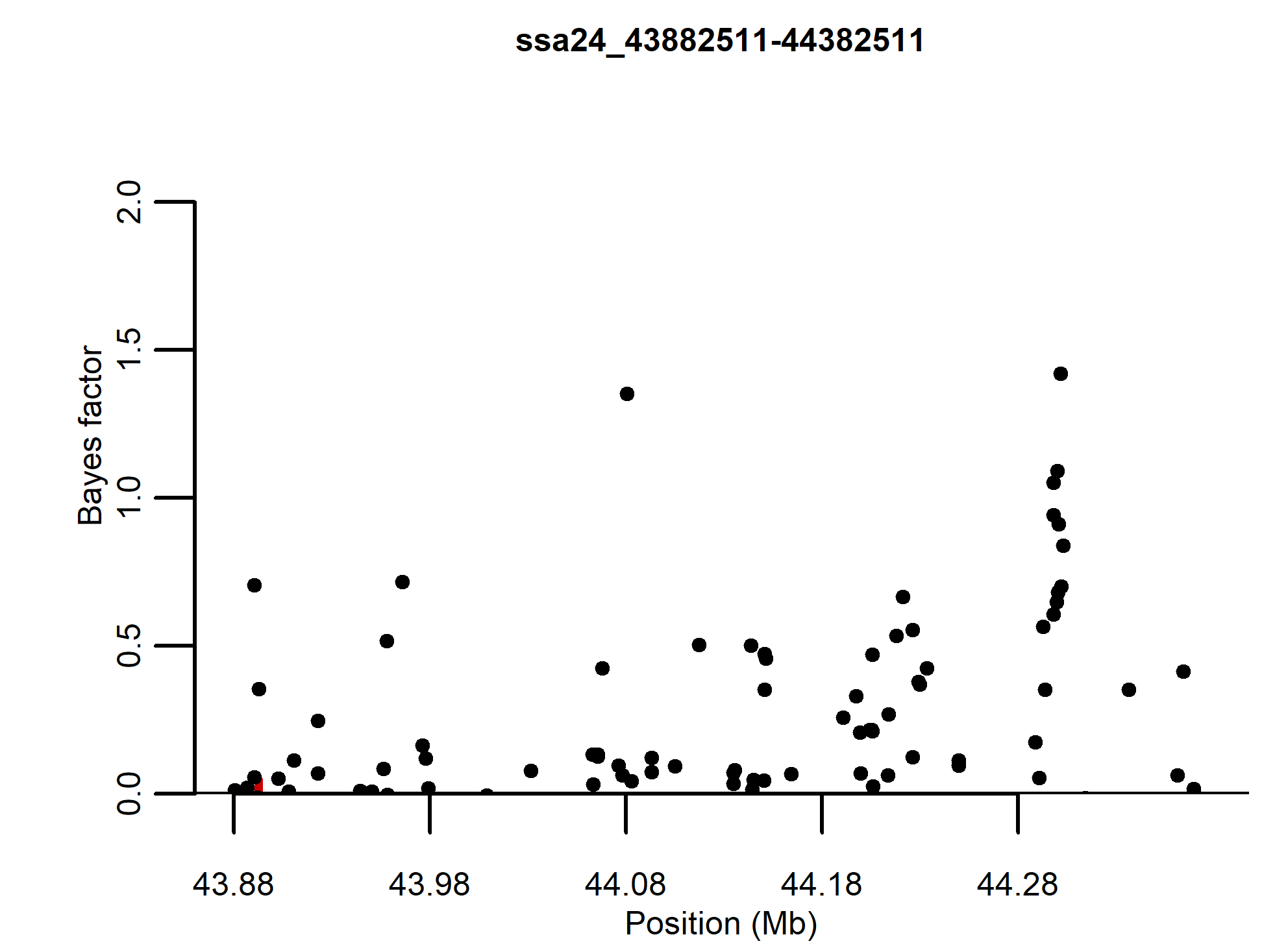

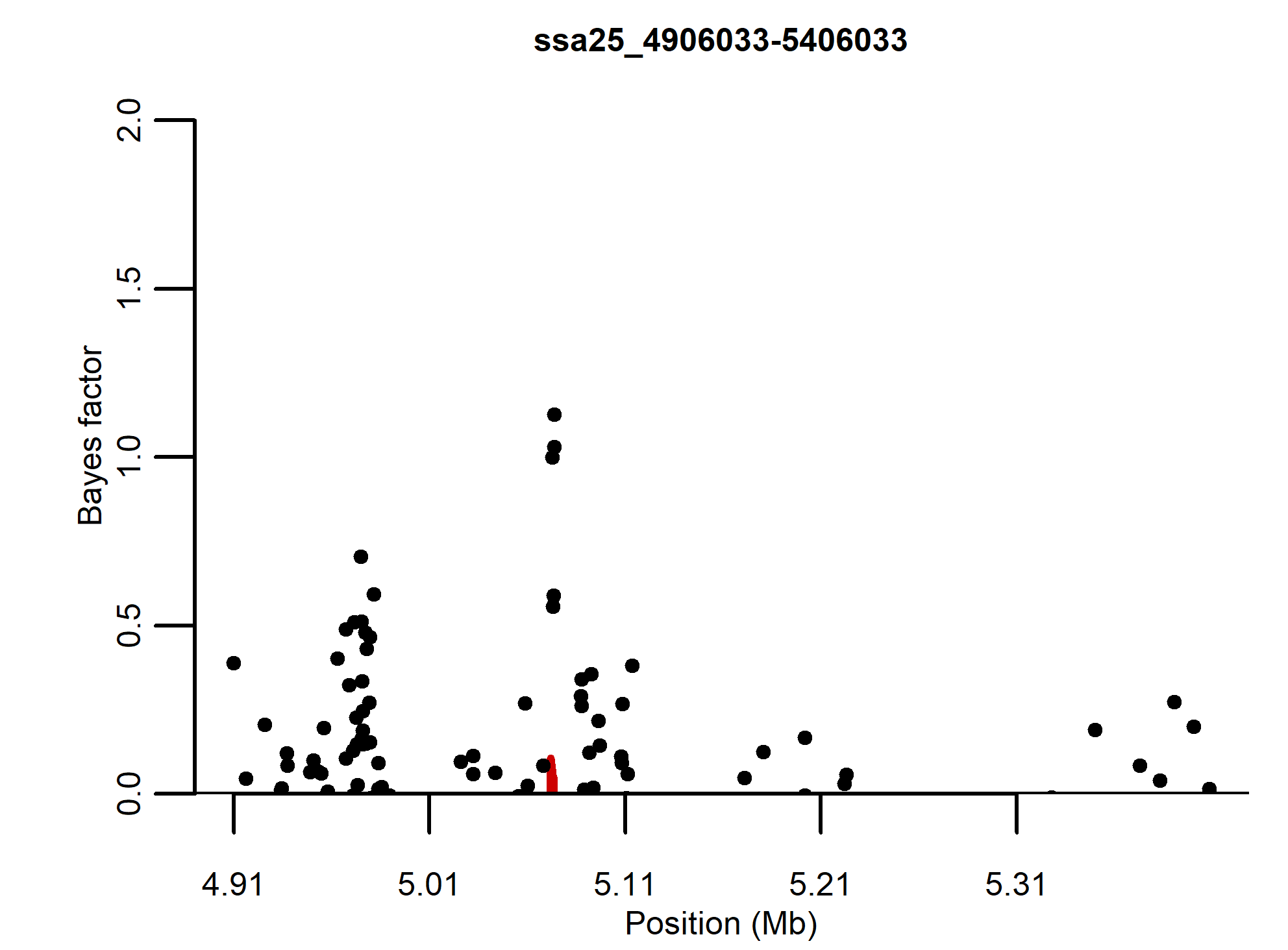

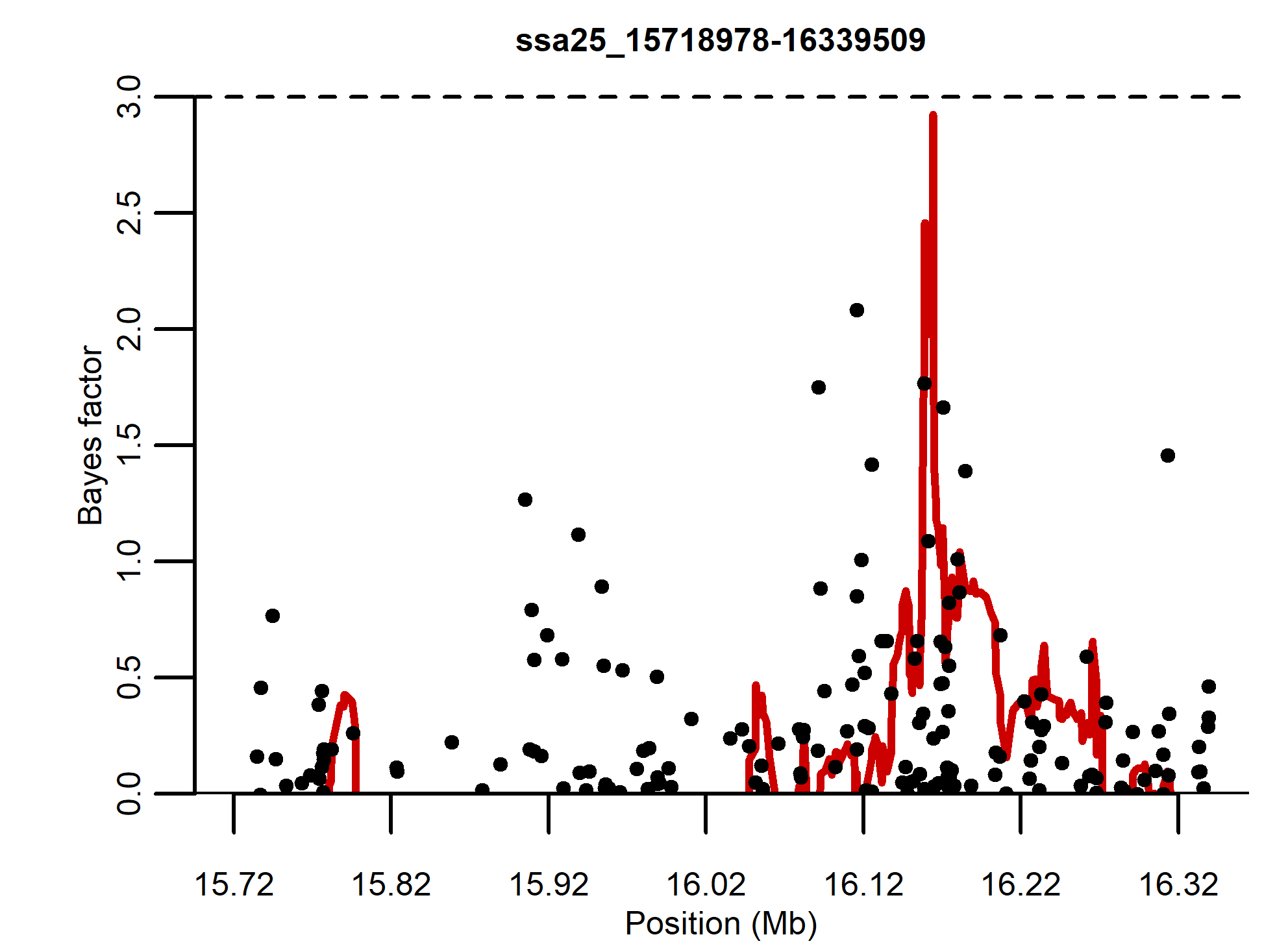

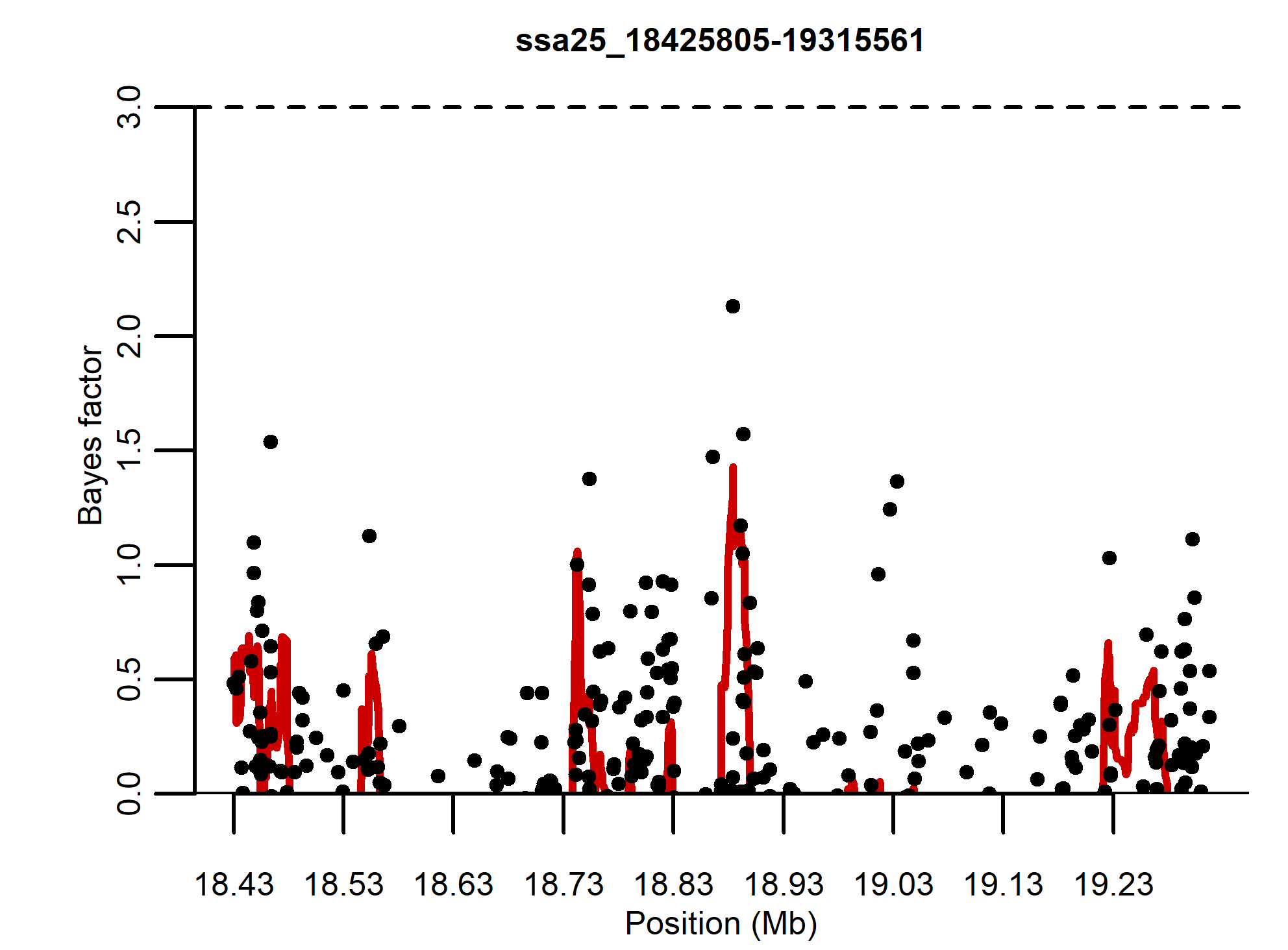

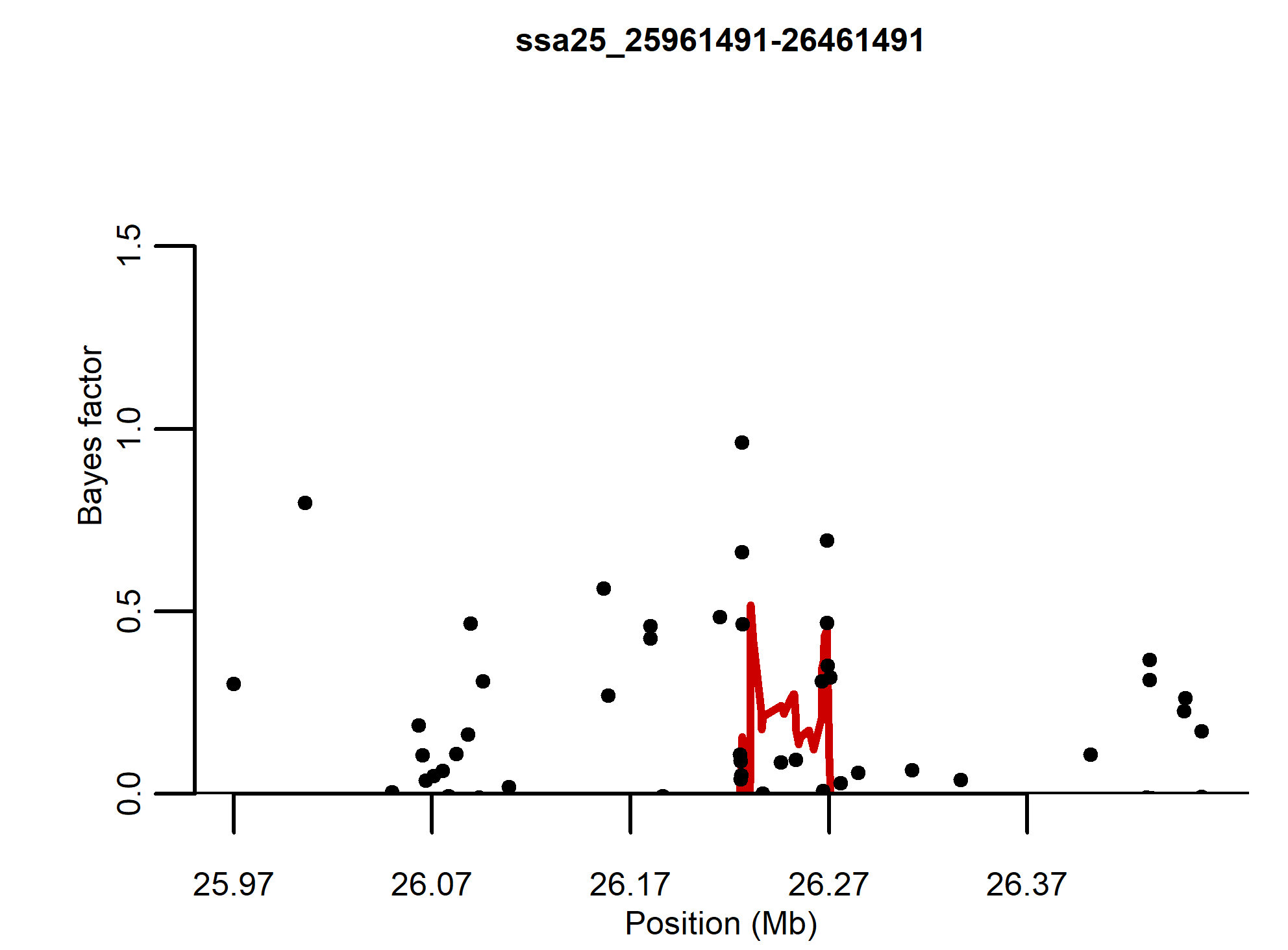

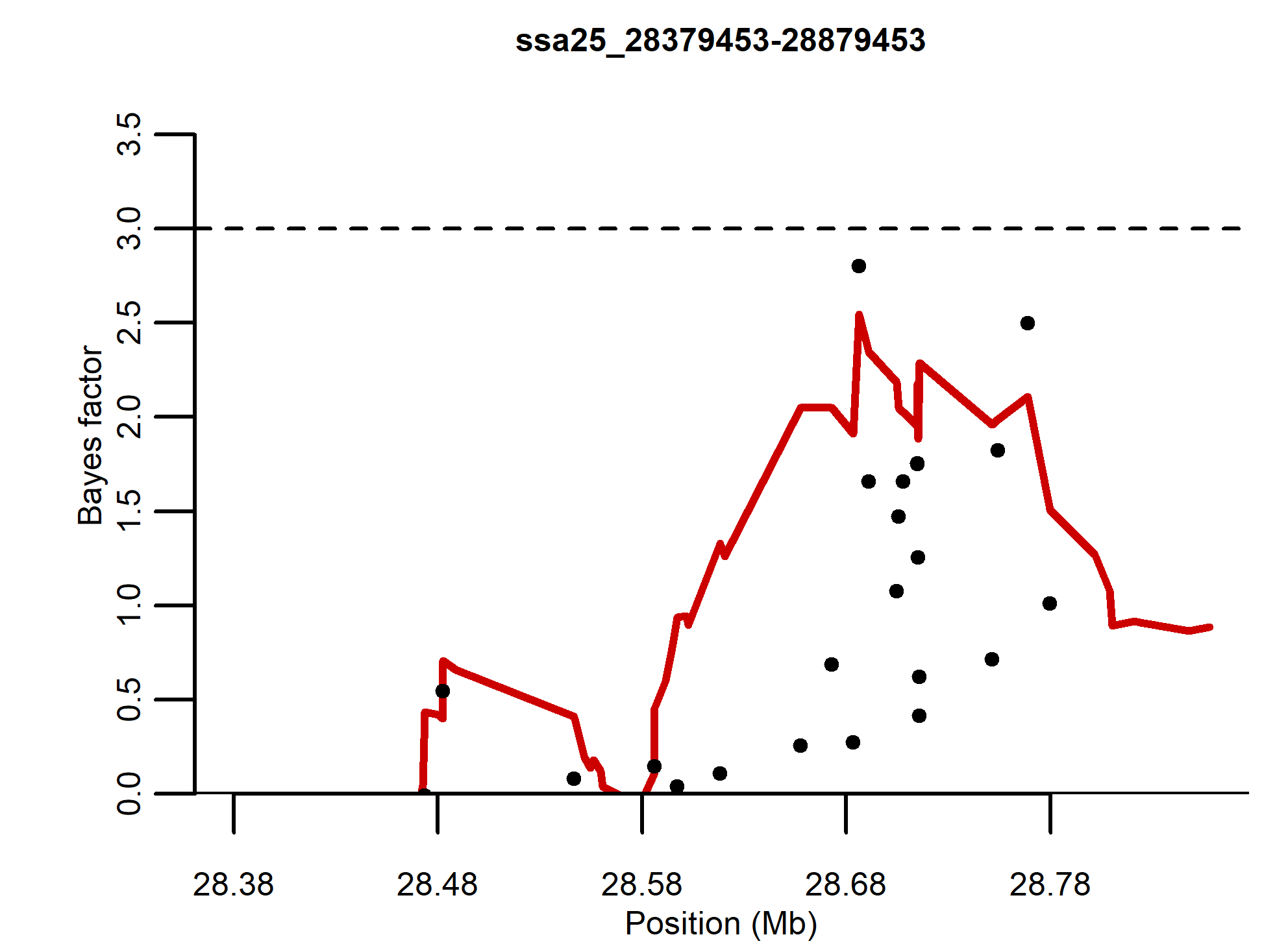

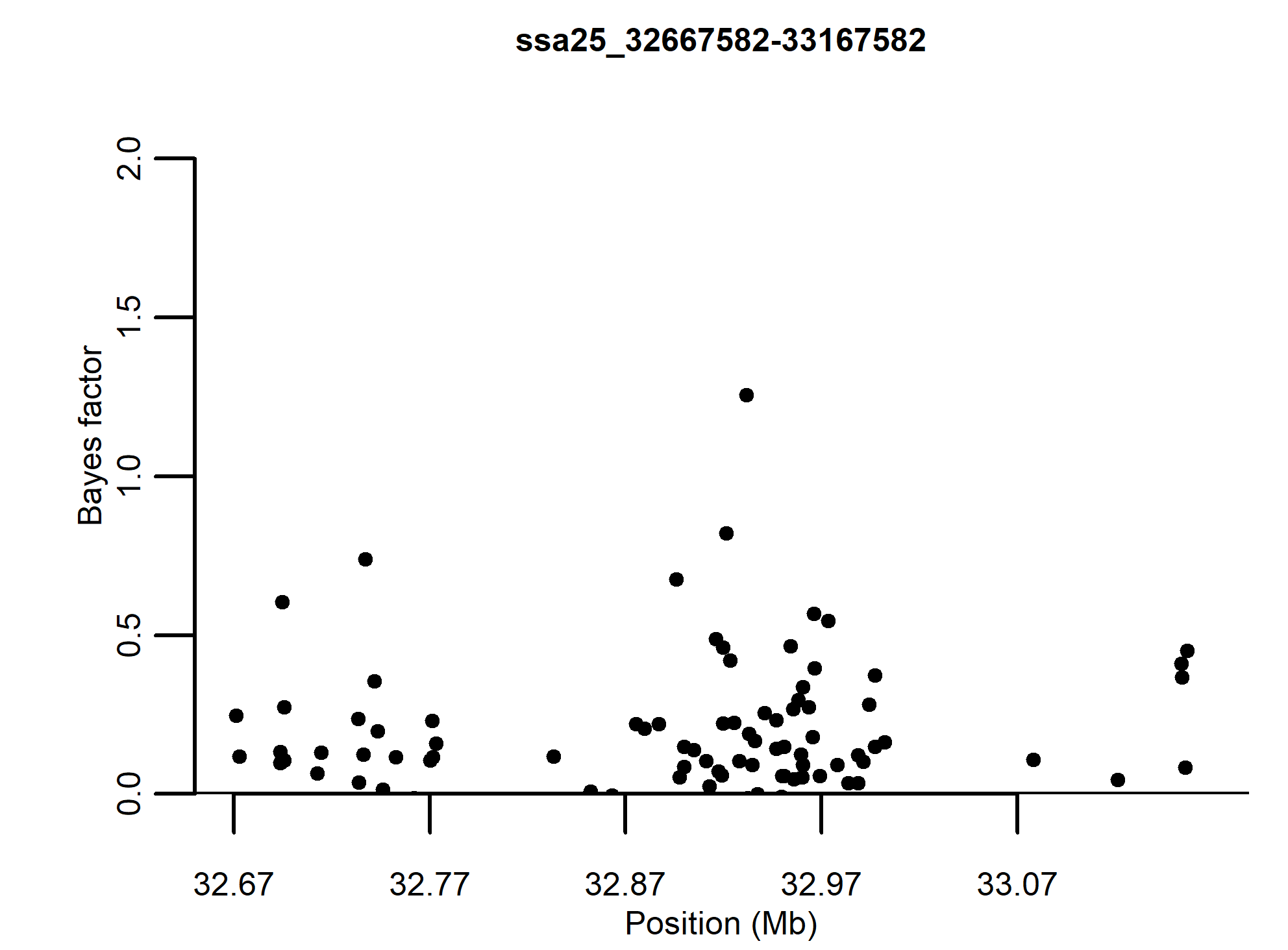

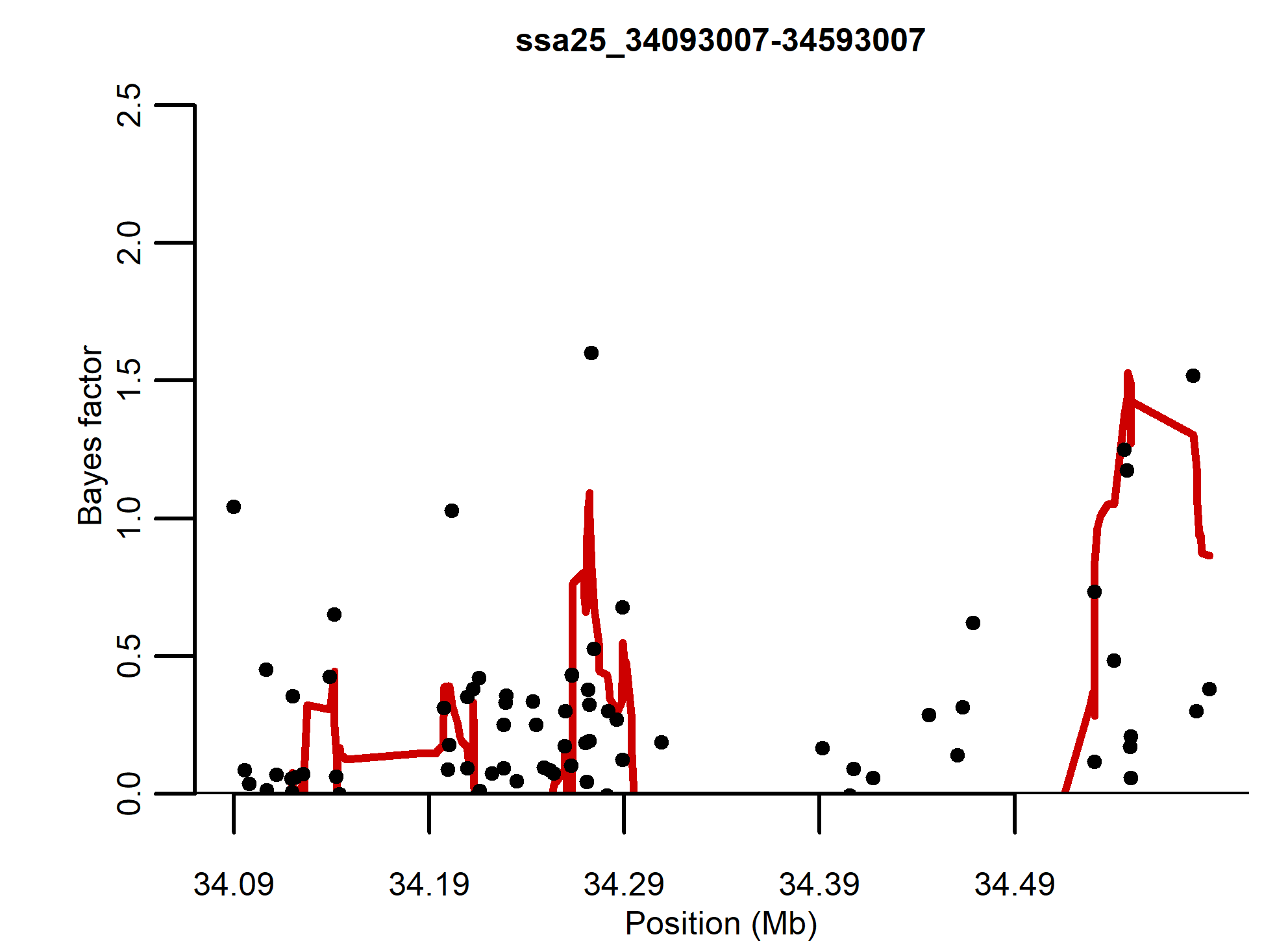

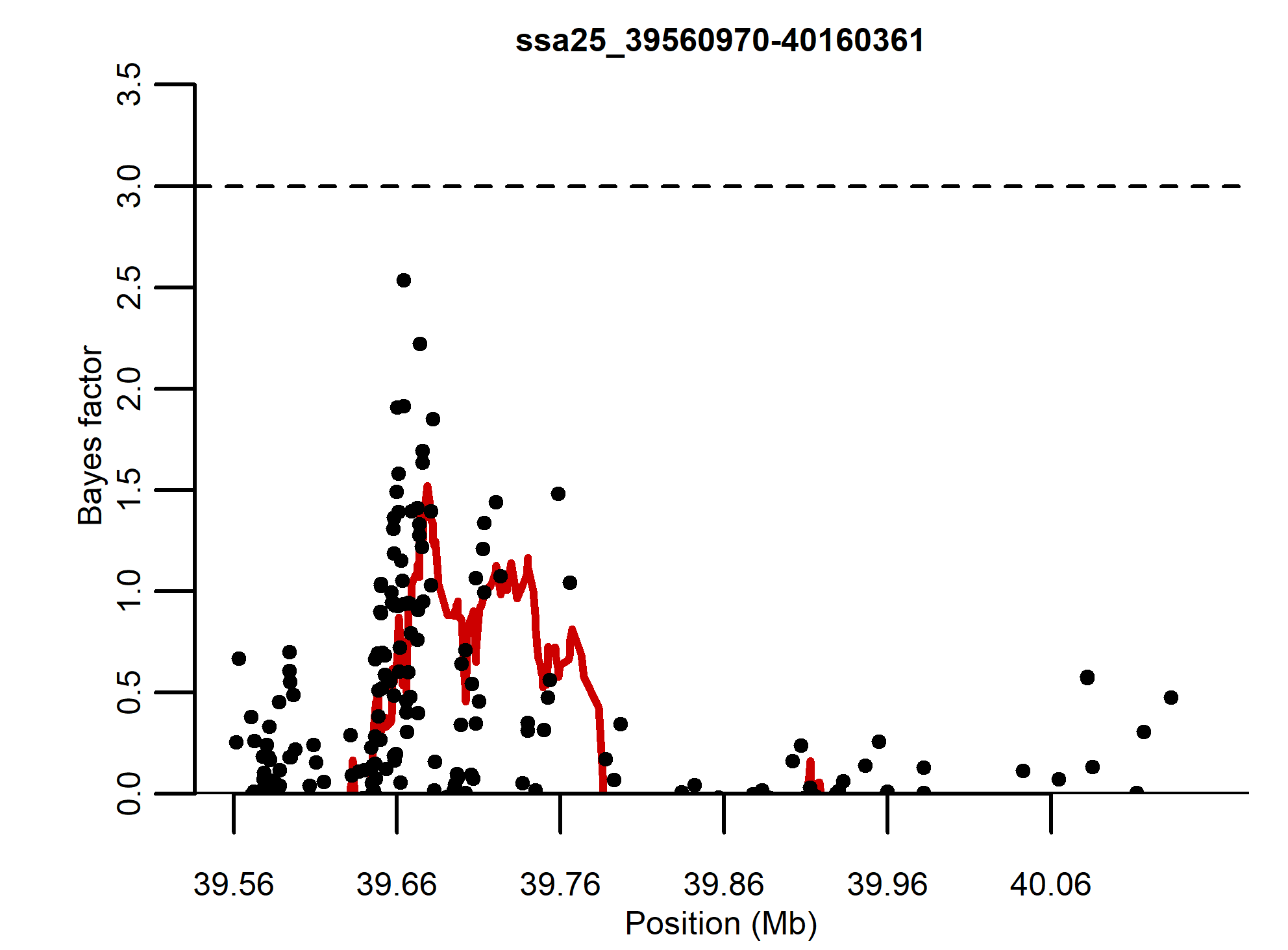

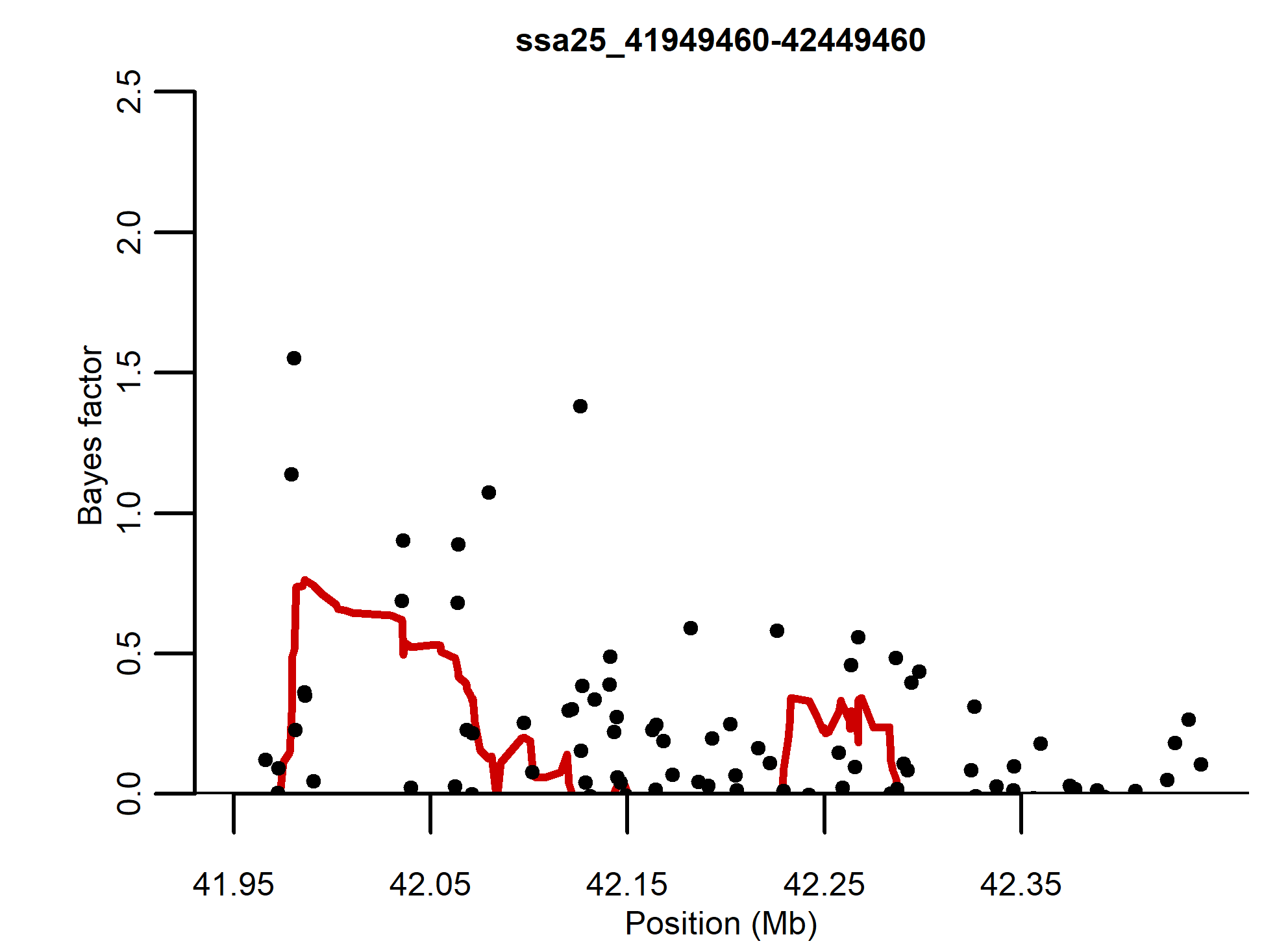

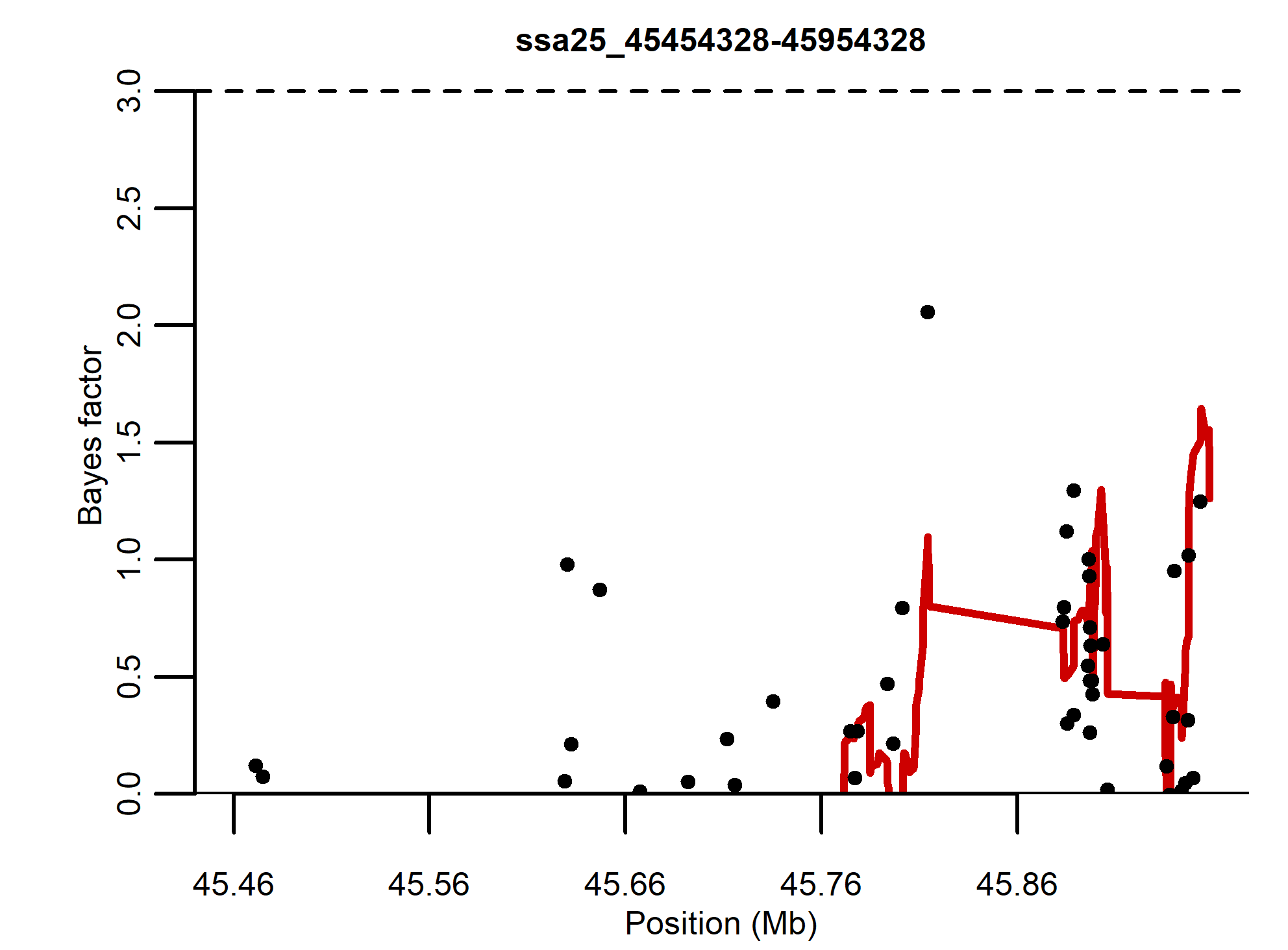

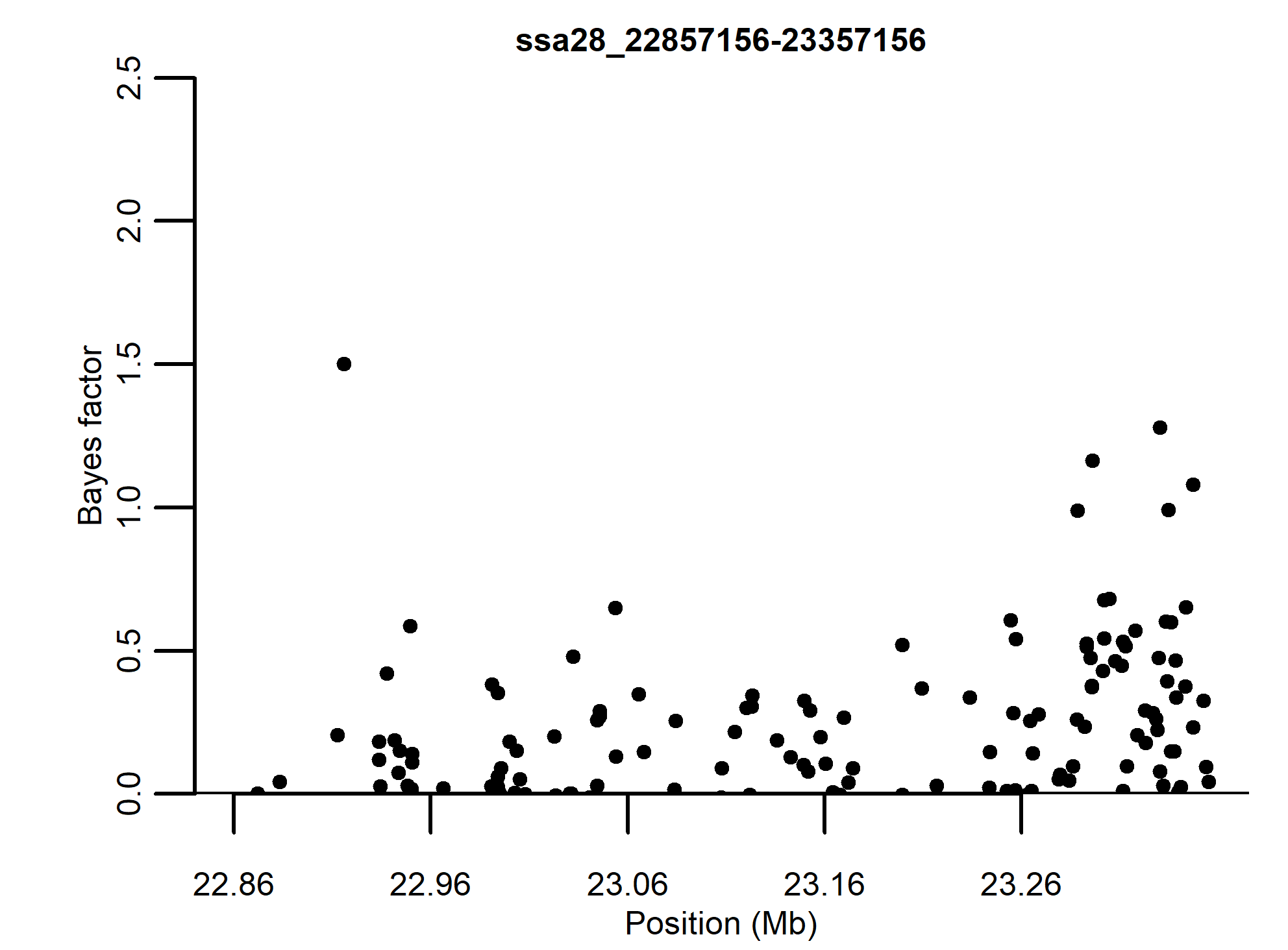


Supplementary Figure S4. Mean sea age for each genotype at the top-associated SNPs per candidate region identified using *PiMASS*. Grey circles are proportional to the number of individuals for each genotype and sea age class (1 year, 2 years, 3+ years).

Supplementary Figure S5. *PiMASS* results after regression of top-associated SNP identified in the initial *PiMASS* analysis. Each candidate region is shown as follows: A. ssa06:27541960-28218141, B. ssa09:10915066-11415066 C. ssa09:24636574-25136574, D. ssa21:49390687-49890687, and E. ssa25:28389273-28889273. Plots display the following results for each candidate region: i) posterior inclusion probability (PIP) indicating the probability of a SNP being included in a model explaining sea age at maturity variation, ii) truncated distribution of the number of SNPs included in a model explaining sea age at maturity variation per recorded iteration (2500) and iii) distribution of proportion of variance explained per recorded iteration (2500) where the red line indicates the median proportion of variance explained.

Supplementary Figure 6. LD plots for each candidate region examined using *PiMASS.* The colour gradient indicates the *R*^2^ value between SNPs where red illustrates a *R*^2^ value of 1 and white illustrates an *R*^2^ value of 0.

Supplementary Figure S7. Path of parameter values from *PiMASS* analyses for each candidate region: A. ssa06:27541960-28218141, B. ssa09:10915066-11415066 C. ssa09:24636574-25136574, D. ssa21:49390687-49890687, and E. ssa25_28389273-28889273. Values of log10 model probability (bf), heritability estimated with SNPs included in model (h), log10 prior probability of SNP being included in model (p) and number of SNPs included in model (snp), were recorded every 1000 steps.

Supplementary Figure S8. Path of parameter values from *PiMASS* analyses for each candidate region following regression of top-associated SNP: A. ssa06:27541960-28218141, B. ssa09:10915066-11415066 C. ssa09:24636574-25136574, D. ssa21:49390687-49890687, and E. ssa25_28389273-28889273. Values of log10 model probability (bf), heritability estimated with SNPs included in model (h), log10 prior probability of SNP being included in model (p) and number of SNPs included in model (snp), were recorded every 1000 steps.
